# Blue light receptor phot2 collaborates with NRL-30 to negatively regulate immunity by reducing mitochondrial protein PRXIIF stability in potato

**DOI:** 10.1186/s43897-025-00224-5

**Published:** 2026-06-02

**Authors:** Huishan Qiu, Yanli Liu, Tianyu Lin, Dong Cheng, Qingguo Sun, Xinya Wu, Lang Liu, Jiahui Nie, Mingshuo Fan, Jing Zhou, Ruimin Yu, Meng Xu, Qin He, Hongying Du, Zhendong Tian

**Affiliations:** 1 National Key Laboratory for Germplasm Innovation & Utilization of Horticultural Crops, Huazhong Agricultural University (HZAU), Wuhan, 430070 China; 2 Hubei Hongshan Laboratory (HZAU), Wuhan, 430070 China; 3 Key Laboratory of Potato Biology and Biotechnology (HZAU), Ministry of Agriculture and Rural Affairs, Wuhan, 430070 China; 4 Potato Engineering and Technology Research Center of Hubei Province (HZAU), Wuhan, 430070 China; 5 Cash Crops Research Institute, Guangxi Academy of Agricultural Sciences (GXAAS), Nanning, 530007 Guangxi China; 6 College of Light Industry and Food Engineering, Nanjing Forestry University, Nanjing, Jiangsu China

**Keywords:** Late blight, *P. infestans*, Blue light receptor, Phototropin, NPH3/RPT2-like protein, Mitochondrial PRXIIF

## Abstract

Blue light (BL) receptor phototropins (phots) regulate numerous BL-induced physiological responses through NPH3/RPT2-like (NRL) family members. However, how phots and NRL proteins regulate plant immunity remain incompletely understood. This study demonstrates that Stphot2 negatively regulated resistance against *Phytophthora infestans*, the causal agent of late blight, in potato and *Nicotiana benthamiana*. Stphot2 interacts with an NRL protein, StNRL-30, which functions as a susceptibility factor. Homodimerization and C-terminal consensus sequence (RxSΦS) of StNRL-30 are essential for accelerating *P. infestans* infection. The study identifies StNRL-30’s interaction with mitochondrial Peroxiredoxin-IIF (StPRXIIF), which positively regulates resistance against *P. infestans*. *StPRXIIF* overexpression upregulates mitochondrial alternative oxidase genes and salicylic acid pathway genes, while downregulating jasmonic acid pathway genes. StNRL-30 initiates StPRXIIF degradation, enhanced by Stphot2. In addition, BL treatment promotes StPRXIIF turnover and induces the translocation of the StNRL-30–StPRXIIF complex from the plasma membrane to the chloroplasts. These findings demonstrate how the BL receptor Stphot2, along with StNRL-30, modulates plant immunity by affecting the stability and localization of the positive immunoregulator StPRXIIF, providing insights into BL and plant immunity crosstalk.

**Supplementary Information:**

The online version contains supplementary material available at 10.1186/s43897-025-00224-5.

## Core

Blue light receptor Stphot2 interacts with an NPH3/RPT2-like (NRL) protein, StNRL-30. Mitochondrial peroxiredoxin-IIF (StPRXIIF) positively regulates plant immunity, but Stphot2, in conjunction with StNRL-30, reduces StPRXIIF stability. Blue light treatment promotes StPRXIIF turnover and induces the translocation of the StNRL-30–StPRXIIF complex from the plasma membrane to the chloroplasts.

## Gene & Accession Numbers

Gene accessions and sequences can be found in the potato genome database (https://spuddb.uga.edu/). Accession numbers: Stphot1 (Soltu.DM.11G026060), Stphot2 (Soltu.DM.01G037020), StNRL-30 (Soltu.DM.09G023040), StPRXIIF (Soltu.DM.01G024400).

## Introduction

Light is a critical environmental factor influencing plant growth and development. Plants respond to various light wavelengths through specialized photoreceptors that direct development, morphogenesis, and physiology. Phototropins (phots) function as plant-specific blue light (BL, 390–500 nm) photoreceptors, playing essential roles in BL-mediated responses (Christie et al. 2015). Phots are plasma membrane (PM)-associated Ser/Thr protein kinases, comprising an AGCVIII (cAMP-dependent protein kinase A, cGMP-dependent protein kinase G, and phospholipid dependent protein kinase C) kinase domain at their C-terminus and two specialized light oxygen voltage (LOV) domains (LOV1 and LOV2) at their N-terminus (Christie et al. 1999; Rademacher and Offringa 2012). BL sensing induces a conformational change that activates the kinase domain and initiates receptor autophosphorylation (Harper et al. 2003; Hart and Gardner 2021; Pfeifer et al. 2010). Kinase-inactive phot mutants impair BL-mediated responses, emphasizing the significance of receptor phosphorylation in signal transduction (Inoue et al. 2008, 2011).

In *Arabidopsis thaliana*, phot1 and phot2 demonstrate both overlapping and distinct functions (Christie 2007). Phot1 and phot2 share partially redundant roles in regulating hypocotyl phototropism under high intensities of unilateral BL, while hypocotyl phototropism under low light conditions is exclusively controlled by phot1 (Sakai et al. 2000, 2001). Both phot1 and phot2 regulate chloroplast accumulation movement under low BL conditions, with phot1 exhibiting greater sensitivity than phot2 (Kagawa and Wadalf 2000; Sakai et al. 2001). Chloroplast avoidance movement in response to high BL conditions is mediated specifically by phot2 (Jarillo et al. 2001; Kagawa et al. 2001). Phot1 directly phosphorylates ATP-BINDING CASSETTE B19 (ABCB19) and PHYTOCHROME KINASE SUBSTRATE 4 (PKS4), thereby inhibiting their auxin transport activity and promoting hypocotyl phototropic responses (Christie et al. 2011; Demarsy et al. 2012). Phot1, but not phot2, modulates ABCB19 activity through phosphorylation (Christie et al. 2011). ROOT CURLING IN N-NAPHTHYLPHTHALAMIC ACID1 (RCN1), corresponding to the A1 subunit of protein phosphatase 2A (PP2A), interacts with phot2 and negatively regulates phot2-mediated phototropism (Tseng and Briggs 2010). However, RCN1 does not interact with phot1. These distinct downstream interacting proteins may account for the functional differences between phot1 and phot2.

NONPHOTOTROPIC HYPOCOTYL 3 (NPH3) and ROOT PHOTOTROPISM 2 (RPT2) are well-characterized members of the NPH3/RPT2-like (NRL) protein family, identified as essential signaling factors for phot-mediated responses (Christie et al. 2018; Liscum et al. 2014). NRL proteins typically comprise three conserved regions: an N-terminal bric-a-brac, tramtrack, and broad complex (BTB) domain, a central NPH3 domain, and a C-terminal coiled-coil domain (Liscum et al. 2014; Suetsugu et al. 2016). NPH3 and RPT2, similar to phots, associate with the PM and interact with phots (Haga et al. 2015; Inada et al. 2004). NPH3, in conjunction with RPT2, participates in phot-mediated changes in phototropism and leaf positioning and expansion (Christie et al. 2015; Harada et al. 2013). NRL PROTEIN FOR CHLOROPLAST MOVEMENT1 (NCH1) and RPT2 redundantly regulate chloroplast accumulation movement in response to low BL conditions (Suetsugu et al. 2016). Studies indicate that NPH3 and RPT2 undergo direct phosphorylation by phot1 within a conserved C-terminal consensus sequence (RxSΦS), necessary for promoting phototropism and leaf positioning in *Arabidopsis*. RxSΦS phosphorylation also triggers 14-3-3 protein binding coupled with changes in NPH3/RPT2 phosphorylation status and NPH3 localization (Reuter et al. 2021; Sullivan et al. 2021; Waksman et al. 2023).

Light and photoreceptors are crucial not only for plant growth and development but also for regulating plant resistance to pathogen attacks (Roeber et al. 2021). The ultraviolet (UV)-B light receptor UVR8 mediates UV-B-induced resistance against *Botrytis cinerea* (Demkura and Ballaré 2012), while the red light receptor PhyB inhibits the BZR1-NAC028-CAD8B pathway to negatively regulate rice resistance to sheath blight (Yuan et al. 2023). Red light induces *Phytophthora capsici* resistance in pepper through CaHY5 expression activation (Yang et al. 2023). Near-infrared light enhances plant antiviral defenses by positively regulating PIF4-activated RNA interference (RNAi) (Zhang et al. 2024), whereas supplemental far-red light increases tomato susceptibility to *B. cinerea* (Courbier et al. 2020, 2021). BL receptors phot2 and CRY2 are essential for the stability of the resistance (R) protein HRT (hypersensitive response to turnip crinkle virus) (Jeong et al. 2010a, b), and CRY1 positively regulates R protein-mediated resistance to *Pseudomonas syringae* (Wu and Yang 2010). These findings demonstrate significant crosstalk between light and plant defense responses.

Potato BL receptors, Stphot1 and Stphot2, have been demonstrated to regulate plant immunity (Naqvi et al. 2022). StNRL1, a cullin-based and BTB domain containing E3 ligase, forms a complex with effector Pi02860 from *P. infestans,* the causal agent of late blight, to facilitate proteasome-mediated degradation of guanine nucleotide exchange factor StSWAP70 (He et al. 2018; Yang et al. 2016). StNRL1 inhibits INF1-induced cell death (ICD), while StSWAP70 is essential for ICD immune response. Both Stphot1 and Stphot2 interact with StNRL1 and function as susceptible factors enhancing *P. infestans* leaf colonization (Naqvi et al. 2022). Stphot1, but not Stphot2, suppresses ICD in a BL- and NRL1-dependent manner and promotes StSWAP70 degradation when co-expressed with StNRL1 (Naqvi et al. 2022), indicating that Stphot2 promotes susceptibility through alternative mechanisms.

Mitochondria serve as cellular energy factories, and mounting evidence indicates their importance in plant–pathogen defense (Igamberdiev and Bykova 2023; Wang et al. 2022). Several mitochondria-localized proteins participate in plant–pathogen interactions. For instance, the mitochondria-associated OsCOX11 is targeted by the effector AvrPita from the fungal pathogen *Magnaporthe oryzae*, leading to the disruption of mitochondrial reactive oxygen species (mROS) metabolism (Han et al. 2021). Alternative oxidase (AOX) functions as a mitochondrial stress marker and plays a crucial role in plant immune regulation (Cvetkovska and Vanlerberghe 2012). Tobacco mosaic virus (TMV) coat protein and transcript levels were elevated in *NbAOX1a*-silenced plants (Zhu et al. 2015). Peroxiredoxins (PRXs) constitute a major peroxidase system in plant mitochondria and act as redox sensors (Liebthal et al. 2018). However, the roles of PRXs in plant immunity remain largely unexplored.

We demonstrated that the expression of *Stphot2* in potato and *Nicotiana benthamiana* enhances *P. infestans* leaf colonization. We identified that Stphot2 interacts with StNRL-30, an NRL family member. Increased expression of StNRL-30 diminishes potato and *N. benthamiana* resistance against *P. infestans*. StNRL-30 interacts with a potato mitochondrial Peroxiredoxin-IIF (StPRXIIF). StPRXIIF functions as a positive regulator that enhances plant resistance against *P. infestans* colonization. In addition, StNRL-30, in conjunction with Stphot2, facilitates proteasome-mediated StPRXIIF degradation. Notably, BL promotes StPRXIIF turnover in the presence of Stphot2 and StNRL-30. Furthermore, BL induces the translocation of the StNRL-30–StPRXIIF complex from the PM to the chloroplasts. This study advances our understanding of how BL receptor phots utilize different downstream NRL family members to modulate plant immunity.

## Results

### Stphot2 acts as a susceptibility factor in regulating late blight resistance

**Fig. 1:**
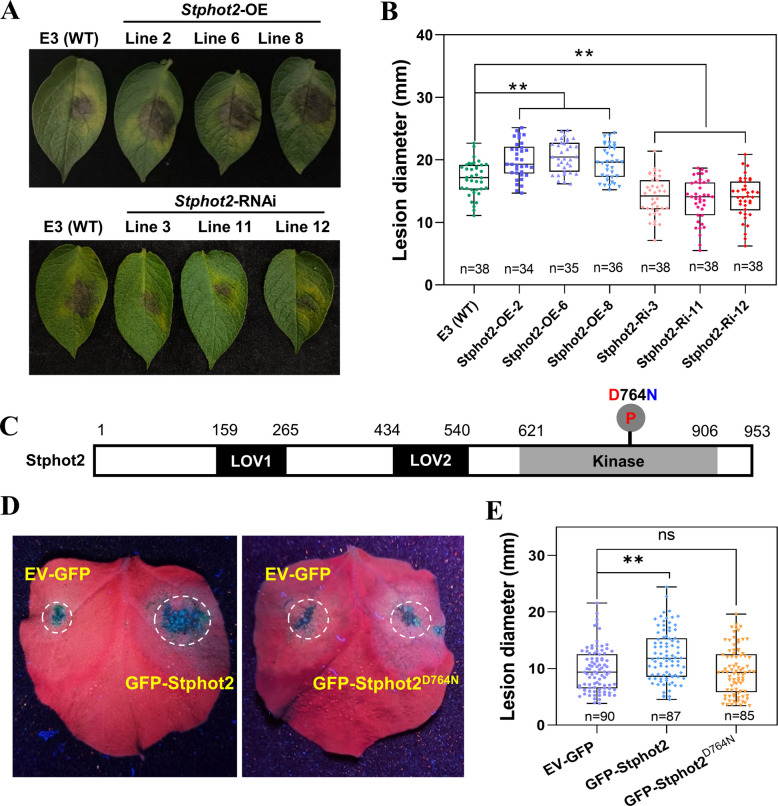
Stphot2 requires kinase activity to enhance *Phytophthora infestans* colonization. **A** and **B** Representative leaf images demonstrating lesion diameters in potato *Stphot2* overexpression (OE) and RNA interference (Ri) lines at 5 days post-inoculation (dpi) with *P. infestans* isolate HB09-14-2, with WT ‘E3’ serving as the control. **C** Schematic diagram of the Stphot2 protein structure. The conserved 764 residue Asp (D) within the Stphot2 kinase domain mutated to Asn (N). **D** and **E** Representative leaf images showing lesion diameters in *Nicotiana benthamiana* at 5 dpi with *P. infestans* isolate 88069. GFP-Stphot2, GFP-Stphot2^D764N^, and EV-GFP were agroinfiltrated into *N. benthamiana* leaves 24 h before inoculation. **B** and **E** Box plots illustrating lesion diameters (mm) on inoculated leaves. Dots represent individual data points, and horizontal lines indicate the median. One-way analysis of variance (ANOVA) was used for statistical analysis (**, *p* < 0.01; three biological replicates with precise n numbers indicated)

In *N. benthamiana*, silencing of *Nbphot1* or *Nbphot2* (alone or in combination) led to a substantial decrease in *P. infestans* leaf colonization (Naqvi et al. 2022). This finding was confirmed in *N. benthamiana* (Fig. S1). To examine the role of Stphot2 in potato late blight resistance, *Stphot2* stable transgenic overexpression (OE) and RNAi (Ri) lines were generated. Three *Stphot2*-OE lines (*Stphot2*-OE-2/6/8) and three *Stphot2*-Ri lines (*Stphot2*-Ri-3/11/12) were selected for further analysis based on the *Stphot2* expression levels (Fig. S2A). These transgenic plants exhibited no apparent phenotypic changes compared with the ‘E-potato 3’ (‘E3’) wild-type (WT) plants after seven weeks of greenhouse growth (Fig. S2B and C). *P. infestans* inoculation assays on detached potato leaves revealed that the OE lines (*Stphot2*-OE-2/6/8) developed significantly larger leaf lesions compared with the WT, while the Ri lines (*Stphot2*-Ri-3/11/12) displayed smaller leaf lesions than the WT (Fig. 1A and B). Thus, Stphot2 functions as a susceptibility factor in regulating late blight resistance in potato, consistent with its role in *N. benthamiana* (Naqvi et al. 2022) (Fig. S1). Similarly, the results indicated that Stphot1 also increased susceptibility to *P. infestans* in transgenic potato plants (Fig. S3).

To examine whether BL influences potato resistance against *P. infestans*, detached leaves of WT ‘E3’ were inoculated with *P. infestans* and placed in incubators with either a 16-h BL/8-h dark cycle or a 16-h white light (WL)/8-h dark cycle. The analysis revealed larger lesions on leaves under BL conditions compared with WL conditions, demonstrating that BL enhances potato susceptibility to *P. infestans* (Fig. S4). To determine whether BL-mediated plant susceptibility requires Stphot1 or Stphot2, *P. infestans* inoculated leaves from *Stphot2*- or *Stphot1*-Ri lines were placed in BL or WL incubators. Under BL conditions, compared with the WT, the lesions on leaves of *Stphot2*- or *Stphot1*-Ri lines were reduced, indicating that BL-mediated plant susceptibility requires Stphot1 or Stphot2 (Fig. S4). However, the lesions on leaves of *Stphot2*- or *Stphot1*-Ri lines under BL conditions remained larger than those under WL conditions (Fig. S4), suggesting that BL-mediated potato susceptibility to *P. infestans* partially required Stphot1 or Stphot2.

In *Arabidopsis*, phot1 and phot2 kinase-dead mutants, where a conserved aspartate residue was substituted with asparagine within the kinase domain, lack autophosphorylation capability (Inoue et al. 2008, 2011). To investigate the importance of Stphot2 kinase activity in enhancing *P. infestans* colonization, the conserved residue Asp764 (D764) within the Stphot2 kinase domain was substituted with Asn (N) to generate the Stphot2^D764N^ mutant (Fig. 1C). Both GFP-Stphot2 and GFP-Stphot2^D764N^ fusion proteins demonstrated stable expression (Fig. S5). GFP-Stphot2 or GFP-Stphot2^D764N^ was transiently expressed on one half of *N. benthamiana* leaves, with the empty vector EV-GFP control on the other half. Subsequently, both sides of each leaf were inoculated with *P. infestans* isolate 88069. Transient expression of GFP-Stphot2 significantly increased lesion diameters compared with the EV-GFP control, while areas expressing the Stphot2^D764N^ mutant exhibited similar lesion sizes to the EV-GFP control (Fig. 1D and E), indicating that Stphot2 kinase activity is essential for enhancing *P. infestans* colonization.

### Stphot2 interacts with StNRL-30 at the PM

The potato genome contains 35 candidate NRL family proteins, excluding those lacking the NPH3 domain (Cheng et al. 2024; Lin et al. 2024). Analysis focused on 12 NRLs (Fig. S6A) containing a C-terminal RxSΦS motif. StRPT2 and StNPH3 were excluded based on previous research showing no effect on *P. infestans* colonization in *N. benthamiana* (Naqvi et al. 2022). Split luciferase (LUC) complementation assay (LCA) was used to identify potential NRL interactions with Stphot2. Using StNRL1 as a positive control for Stphot2 interaction, strong luminescence signals were detected upon co-expression of Stphot2-nLUC and cLUC-StNRL-30 (Fig. S6B). This led to the selection of StNRL-30 as a candidate interacting protein for Stphot2.

**Fig. 2:**
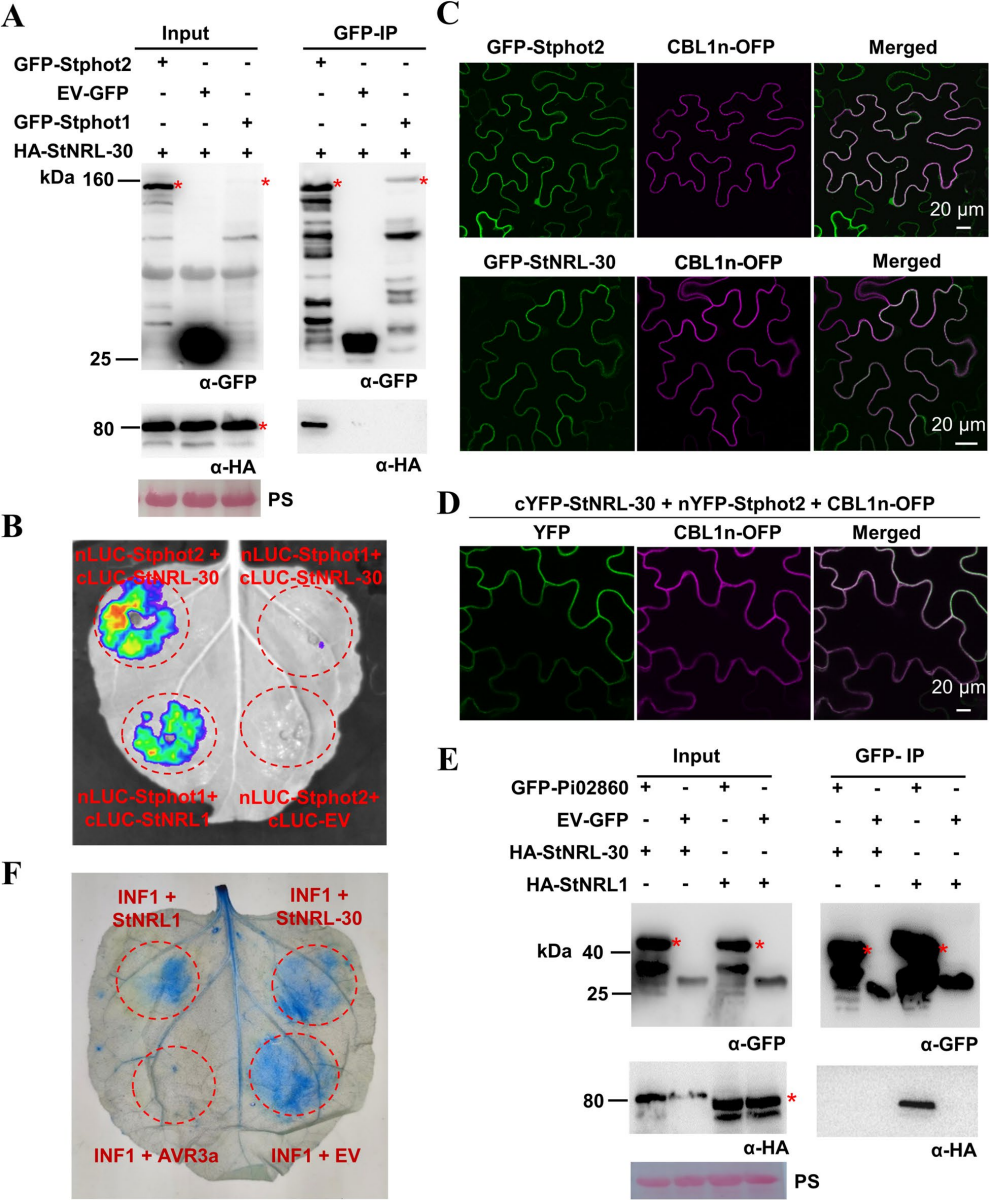
StNRL-30 interacts with Stphot2, but not with Stphot1 and Pi02860. **A** Co-immunoprecipitation (co-IP) assay demonstrated that HA-Stphot2, but not HA-Stphot1, was immunoprecipitated with GFP-StNRL-30. EV-GFP served as the negative control. GFP-agarose beads were used for immunoprecipitating leaf extract proteins. **B** LUC complementation assay (LCA) confirmed that StNRL-30 interacts with Stphot2, but not with Stphot1. The luminescence signal was detected and imaged 48 h post-agroinfiltration (hpa). **C** Confocal microscopy images demonstrating that GFP-StNRL-30 and GFP-Stphot2 localized to the plasma membrane (PM). GFP-StNRL-30 and GFP-Stphot2 were transiently co-expressed with the PM marker CBL1n-OFP in *N. benthamiana*. Images were captured at 48 hpa. **D** BiFC assay revealing that Stphot2 interacts with StNRL-30 on the PM. cYFP-StNRL-30 and nYFP-Stphot2 were transiently co-expressed with CBL1n-OFP in *N. benthamiana*. YFP fluorescence coincided with OFP fluorescence at 48 hpa. **E** Co-IP assay indicated that HA-StNRL1, but not HA-StNRL-30, was immunoprecipitated with GFP-Pi02860. EV-GFP served as the negative control. GFP-agarose beads were used for immunoprecipitating leaf extract proteins. Constructs expressed in *N. benthamiana* leaves are represented by a plus sign (+) in** A** and** E**. Protein size markers are given in kilodaltons (kDa), and protein loading is shown by Ponceau stain (PS). * indicates target protein bands. **F** Representative trypan blue-stained leaf image showing INF1-induced cell death when INF1 was co-expressed with EV-GFP, GFP-StNRL-30, GFP-StNRL1, and GFP-AVR3a, in *N. benthamiana* leaves at 48 hpa. EV-GFP served as the negative control, and GFP-AVR3a functioned as the positive control

Co-immunoprecipitation (co-IP) assay confirmed the interaction between Stphot2 and StNRL-30. HA-Stphot2 and HA-Stphot1 were transiently co-expressed with either GFP-StNRL-30 or EV-GFP in *N. benthamiana* leaves. HA-Stphot2, but not HA-Stphot1, was co-immunoprecipitated by GFP-StNRL-30 (Fig. 2A and Fig. S6C). LCA results demonstrated that regions co-expressing Stphot2-nLUC and cLUC-StNRL-30 exhibited comparable LUC activity to the positive control (Stphot1-nLUC + cLUC-StNRL1) (Fig. 2B). In contrast, areas co-expressing Stphot1-nLUC + cLUC-StNRL-30 and Stphot2-nLUC + cLUC-EV showed no LUC activity (Fig. 2B). These findings confirmed that Stphot2, but not Stphot1, interacts with StNRL-30 *in planta*.

In *Arabidopsis*, phot1, phot2, NPH3, and RPT2 are PM-associated proteins (Hart and Gardner 2021). Confocal images confirmed that Stphot2 was co-localized with the PM marker CBL1n (Qiu et al. 2023) (Fig. 2C). GFP-StNRL-30 showed significant accumulation on the PM (Fig. 2C and Fig. S7A). Bimolecular fluorescence complementation (BiFC) assay was used to examine the interaction site of Stphot2 and StNRL-30 in plant cells. Yellow fluorescent protein (YFP) fluorescence was detected in regions co-expressing nYFP-Stphot2 and cYFP-StNRL-30 (Fig. 2D), with the YFP signal coinciding with the PM marker CBL1n-OFP (orange fluorescent protein). No fluorescence was observed in areas co-expressing cYFP-StNRL-30 and nYFP-Stphot1 or cYFP-EV and nYFP-Stphot2 (Fig. S7B). These results collectively indicate that Stphot2 interacts with StNRL-30 on the PM.

StNRL1, targeted by the *P. infestans* RXLR effector Pi02860, inhibits the ICD immune response (Yang et al. 2016). To examine whether StNRL-30 exhibits similar functions as StNRL1, co-IP assays were conducted to assess the interaction between StNRL-30 and Pi02860. The results demonstrated that HA-StNRL1, but not HA-StNRL-30, was immunoprecipitated with GFP-Pi02860 (Fig. 2E). Neither HA-StNRL1 nor HA-StNRL-30 was immunoprecipitated with the EV-GFP control. To evaluate potential ICD suppression in the presence of StNRL-30, INF1 was co-expressed with GFP-StNRL-30, GFP-StNRL1, GFP-AVR3a, or EV-GFP. GFP-StNRL1 and GFP-AVR3a suppressed ICD, while GFP-StNRL-30 showed no effect on ICD (Fig. 2F). Thus, StNRL-30 neither interacts with Pi02860 or Stphot1 nor suppresses ICD, suggesting distinct functional characteristics from StNRL1.

### StNRL-30 acts as a susceptibility factor to *P. infestans* resistance

**Fig. 3:**
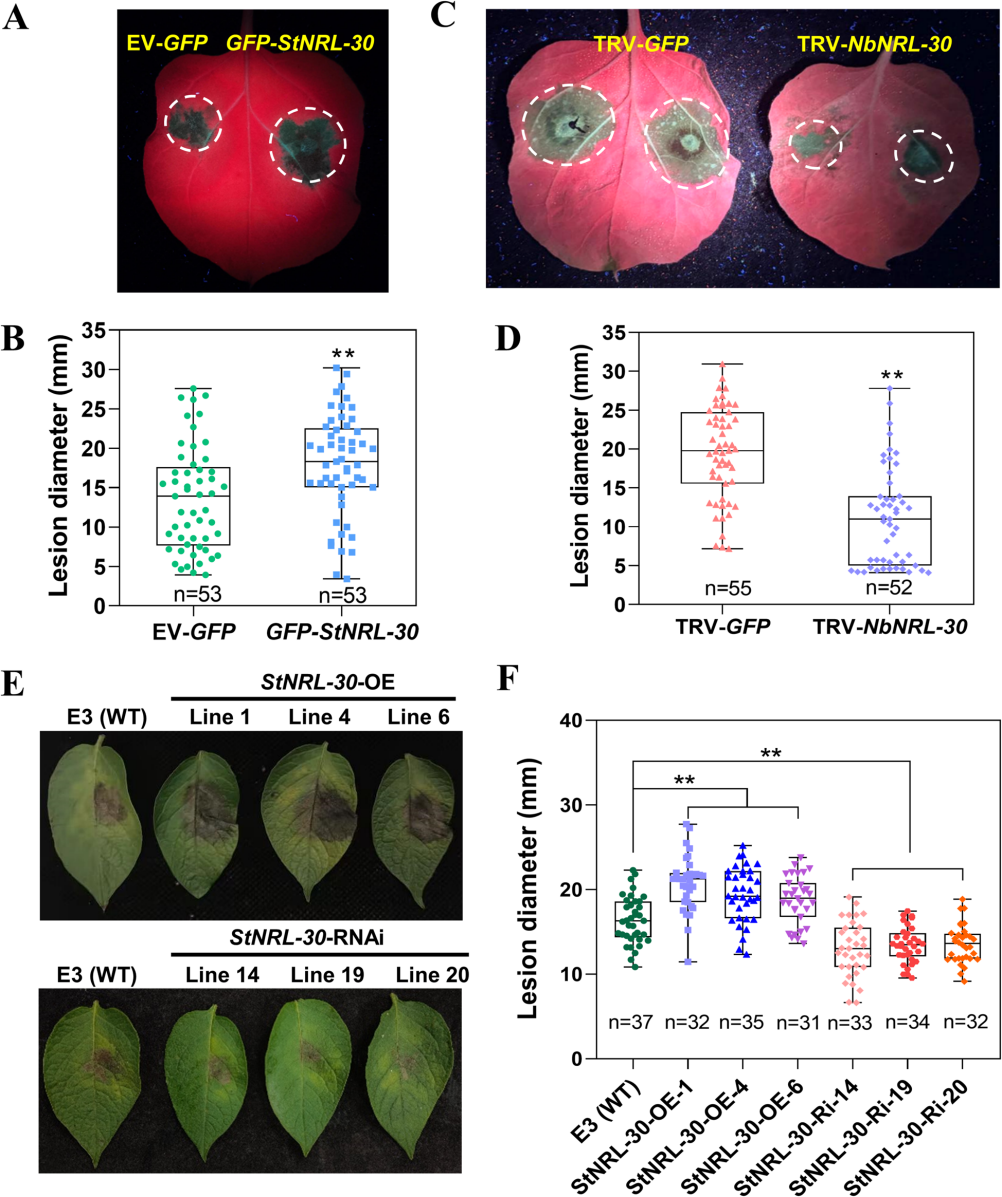
StNRL-30 enhances *P. infestans* colonization. **A** and** B** Representative images showing lesion diameters in *N. benthamiana* leaves agroinfiltrated with *GFP-StNRL-30* and EV-*GFP* at 5 dpi with *P. infestans* isolate 88069. **C** and** D** Leaves from TRV-*NbNRL-30* plants exhibited reduced lesion sizes compared with plants expressing TRV-*GFP* at 6 dpi. Leaves were inoculated with *P. infestans* isolate 88069. **E** and** F** Representative images showing lesion diameters on *StNRL-30*-OE and -Ri lines at 5 dpi with *P. infestans* isolate HB09-14-2, using WT ‘E3’ as the control. **B**, **D**, and **F** Box plots illustrating lesion diameters (mm) on inoculated leaves. Dots indicate individual data points, and horizontal lines represent the median. Statistical analysis was performed using one-way ANOVA (**, *p* < 0.01; three independent repeats with precise n numbers indicated

To examine the function of StNRL-30 in disease resistance, GFP-StNRL-30 and EV-GFP were transiently expressed in *N. benthamiana* leaves. Following inoculation, significantly larger lesions were observed in areas expressing GFP-StNRL-30 compared with areas expressing the EV-GFP control (Fig. 3A and B). Two sequences, designated *NbNRL-30a* and *NbNRL-30b*, encoding proteins with 96.4% amino acid identity to each other, were identified in the *N. benthamiana* genome. The predicted proteins of NbNRL-30a and NbNRL-30b share 93.6% and 90.5% identity with StNRL-30, respectively (Fig. S8). Subsequently, the virus-induced gene silencing (VIGS) construct tobacco rattle virus (TRV)-*NRL-30* was designed to silence both homologs simultaneously. The transcription level of *NbNRL-30* decreased by 65–90% in *N. benthamiana* plants expressing TRV-*NRL-30* compared with plants expressing the TRV-*GFP* control (Fig. S9A). TRV-*NRL-30* plants exhibited stunted developmental phenotype compared with TRV-*GFP* plants (Fig. S9B). Leaves from *N. benthamiana* plants expressing TRV-*NRL-30* displayed significantly smaller lesions compared with TRV-*GFP* (Fig. 3C and D). Transient overexpression and VIGS results indicated that NRL-30 facilitated *P. infestans* colonization.

To investigate the role of StNRL-30 in modulating potato susceptibility to *P. infestans*, transgenic potato *StNRL-30*-OE lines and *StNRL-30*-Ri lines were generated (Fig. S9). Three *StNRL-30*-OE lines (*StNRL-30*-OE-1/4/6) and three *StNRL-30*-Ri lines (*StNRL-30*-Ri-14/19/20) were selected for detailed analysis based on their *StNRL-30* expression levels as determined by quantitative real-time polymerase chain reaction (qRT-PCR) (Fig. S9C). The transgenic plants exhibited no apparent phenotypic differences compared with the WT ‘E3’ (Fig. S9D and E). When challenged with the *P. infestans* isolate HB09-14-2, the OE lines (*StNRL-30*-OE-1/4/6) developed significantly larger lesions compared with the WT, while the Ri lines (*StNRL-30*-Ri-14/19/20) showed significantly smaller lesions compared with the WT (Fig. 3E and F). These findings confirmed that StNRL-30 functions as a susceptibility factor, negatively regulating late blight resistance in potato and *N. benthamiana*.

### Homodimerization and RXSXS motif are important for StNRL-30 to enhance *P. infestans* colonization

**Fig. 4:**
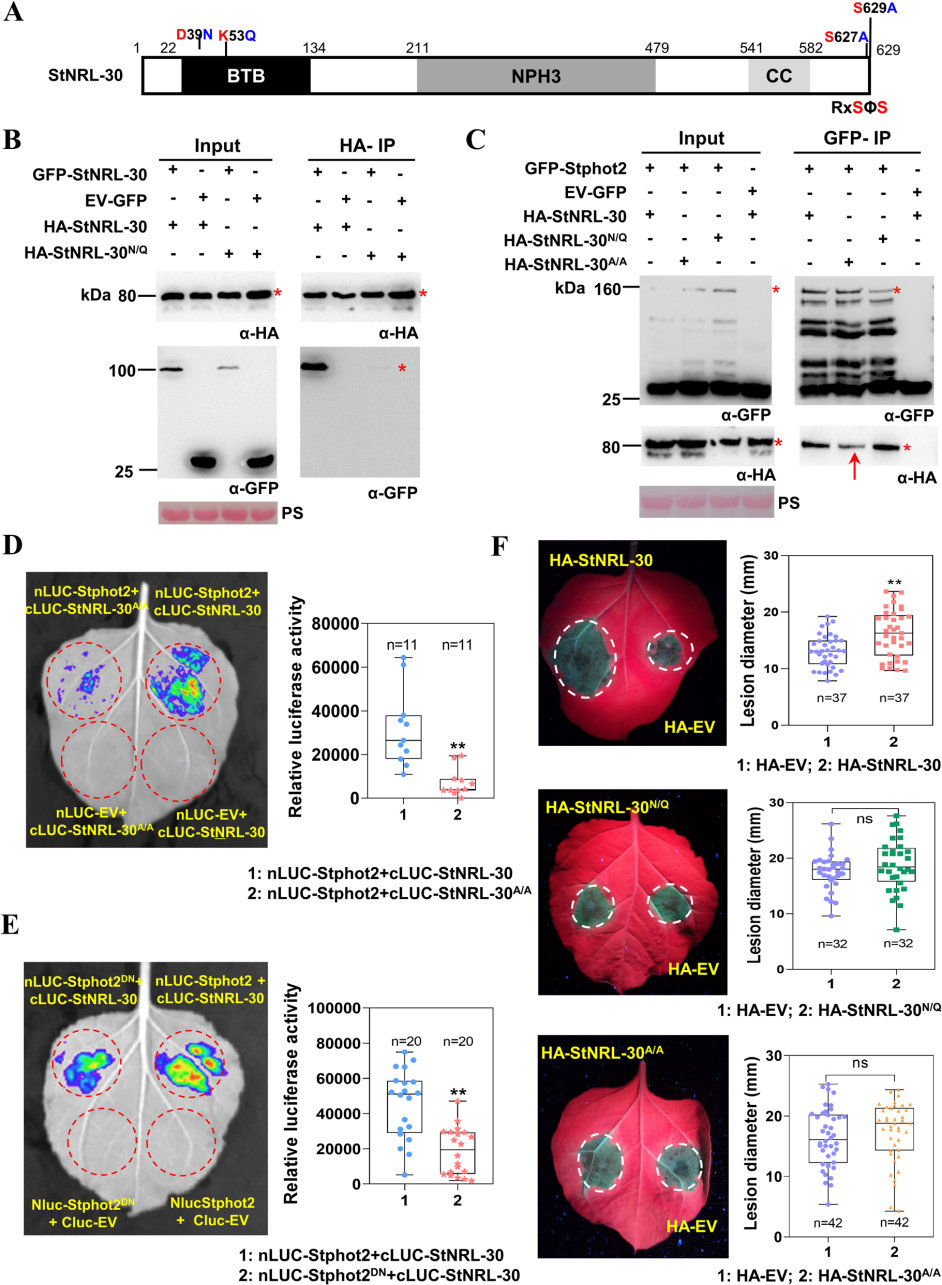
Homodimerization and RxSΦS motif are essential for StNRL-30 to promote *P. infestans* colonization. **A** Schematic diagram of the StNRL-30 protein structure. BTB: bric-a-brac, tramtrack, and broad complex domain; NPH3: NONPHOTOTROPIC HYPOCOTYL 3 domain; CC: coiled-coil domain. The conserved residues in StNRL-30 (Asp39 and Lys53) were mutated to Asn (N) and Gln (Q) to generate the StNRL-30^N/Q^ mutant. Ser627 and Ser629 in the C-terminal RxSΦS motif were mutated to Ala (A) to generate the StNRL-30^A/A^ mutant. **B** Co-IP assay demonstrating weak association between HA-StNRL-30^N/Q^ and GFP-StNRL-30, and strong association between HA-StNRL-30 and GFP-StNRL-30. HA-agarose beads were used for immunoprecipitating leaf extract proteins. **C** Co-IP assay revealing the interaction between GFP-Stphot2 and HA-StNRL-30 mutants. GFP-agarose beads were used for immunoprecipitating leaf extract proteins. Constructs were transiently expressed in *N. benthamiana* leaves through agroinfiltration. Constructs expressed in *N. benthamiana* leaves are indicated by a plus sign (+) in **B** and **C**. Protein size markers are presented in kDa, and protein loading is indicated by PS. * indicates target protein bands. Red arrows indicate weakened target bands. **D** and** E** LCA demonstrating significantly weaker interactions between cLUC-StNRL-30^A/A^ and nLUC-Stphot2, and between cLUC-StNRL-30 and nLUC-Stphot2^D764N^, compared with cLUC-StNRL-30 and nLUC-Stphot2. Box plots illustrate the relative LUC activity obtained by the IndiGo analysis. Dots represent individual data points, and horizontal lines indicate the median. **F** Representative leaf images and box plots showing lesion diameters in HA-StNRL-30, HA-StNRL-30^N/Q^, HA-StNRL-30^A/A^, and HA-EV agroinfiltrated *N. benthamiana* leaves. Lesion diameters were measured at 5 dpi with *P. infestans* isolate 88069. Dots represent individual data points, and horizontal lines indicate the median. Student’s *t*-test was used for statistical analysis (**, *p* < 0.01; three independent repeats with precise n numbers indicated)

StNRL1 forms a homodimer, dependent on the conserved pocket residues within the BTB domain (He et al. 2018). Co-IP assay revealed that HA-StNRL-30 was immunoprecipitated with GFP-StNRL-30, but not with GFP-StNRL1 (Fig. S10A), indicating StNRL-30 forms a homodimer. Subsequently, the conserved residues in StNRL-30 (Asp39 and Lys53) were mutated to Asn (39N) and Gln (53Q) to generate the StNRL-30^D39N/K53Q^ (StNRL-30^N/Q^) mutant (Fig. 4A). Co-IP assay was performed to examine its effect on StNRL-30 dimerization. The results demonstrated that GFP-StNRL-30 was strongly co-immunoprecipitated by HA-StNRL-30, while HA-StNRL-30^N/Q^ displayed markedly weakened associations with GFP-StNRL-30 (Fig. 4B), suggesting that the two conserved residues of the BTB domain are essential for StNRL-30 dimerization.

In *Arabidopsis*, phot1 directly phosphorylates the conserved C-terminal consensus sequence (RxSΦS) of NPH3 and RPT2, accompanied by 14-3-3 binding, which is necessary to promote phototropism and leaf positioning (Reuter et al. 2021; Sullivan et al. 2021; Waksman et al. 2023). The C-terminal RxSΦS motif for phosphorylation is conserved in StNRL-30 (Fig. S8), suggesting that phot-mediated phosphorylation and 14-3-3 binding play important functions in regulating *P. infestans* resistance. Co-IP results demonstrated that both HA-Stphot2 and HA-StNRL-30 interacted with GFP-St14-3-3, but not with the EV-GFP control (Fig. S10B and C). The two serine residues (Ser627 and Ser629) within the RxSΦS motif were mutated to Ala (A) to generate the StNRL-30^S627A/S629A^ (StNRL-30^A/A^) mutant (Fig. 4A). Co-IP assay revealed that mutant StNRL-30^A/A^ maintained weak interaction with St14-3-3 (Fig. S10C).

Further investigation examined whether the S627A/S629A and D39N/K53Q mutants affect the StNRL-30 interaction with Stphot2 through co-IP assays. The results indicated that the StNRL-30^A/A^ mutant exhibited attenuated interaction with Stphot2, while the StNRL-30^N/Q^ mutant maintained normal interaction with Stphot2 (Fig. 4C). LCA results demonstrated that the interactions between StNRL-30^A/A^ and Stphot2 or StNRL-30 and Stphot2^D764N^ were reduced compared with the interactions between StNRL-30 and Stphot2 (Fig. 4D and E).

Ser627 and Ser629 of StNRL-30 were identified as potential phosphorylation sites (Fig. S11A) and 14-3-3 binding sites (Fig. S11B). In addition, AlphaFold3 analysis indicated that these serine residues serve as interaction sites for Stphot2, with Ser627 specifically identified as a potential phosphorylation site (Fig. S11C). This finding aligns with previous research demonstrating that NRLs undergo direct phosphorylation by phots within the conserved sequence (RxSΦS), which subsequently facilitates 14-3-3 binding (Sullivan et al. 2021).

Late blight resistance was also investigated using StNRL-30 mutants. *P. infestans* inoculation assays demonstrated that the transient expression of HA-StNRL-30 significantly enhanced *P. infestans* colonization compared with the HA-EV control, while the expression of mutants HA-StNRL-30^N/Q^ and StNRL-30^A/A^ showed no such enhancement (Fig. 4F). Thus, both homodimerization and the RxSΦS motif of StNRL-30 are essential for conferring susceptibility to *P. infestans*.

### StNRL-30 interacts with the positive regulator StPRXIIF

**Fig. 5:**
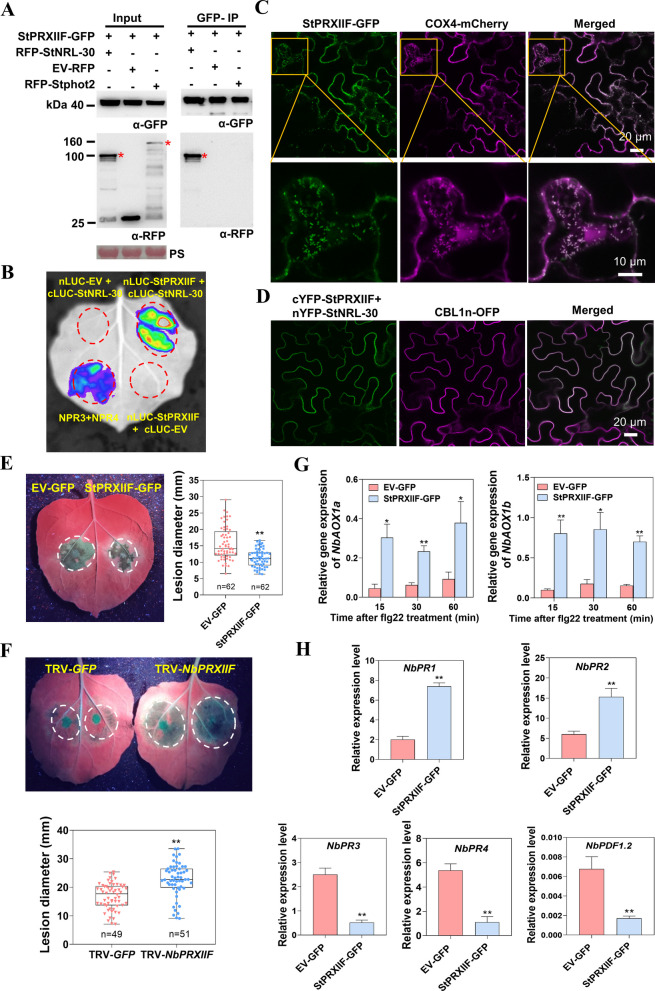
StNRL-30 associates with the positive immunoregulator StPRXIIF.** A** Co-IP assay demonstrating that StPRXIIF-GFP interacts with RFP-StNRL-30. GFP-agarose beads were used for immunoprecipitating leaf extract proteins. Constructs were transiently expressed in *N. benthamiana* leaves through agroinfiltration. Constructs expressed in *N. benthamiana* leaves are indicated by a plus sign (+). Protein size markers are indicated in kDa, and protein loading was visualized by PS. * indicates target protein band. **B** LCA validating the interaction between StPRXIIF and StNRL-30. The luminescence signal was detected and imaged at 48 hpa. **C** Confocal images revealing that StPRXIIF-GFP co-localized with a mitochondrial marker COX4-mCherry in *N. benthamiana* cells. Images were captured at 48 hpa. **D** BiFC assay demonstrating that StPRXIIF interacts with StNRL-30 at the PM. cYFP-StPRXIIF, along with nYFP-StNRL-30, was transiently co-expressed with CBL1n-OFP in *N. benthamiana* leaves. Images were captured at 48 hpa. **E** Representative images and box plots illustrating lesion diameters in *N. benthamiana* leaves at 5 dpi with *P. infestans* isolate 88069. Dots represent individual data points, and horizontal lines indicate the median. **F** Leaves from TRV-*NbPRXIIF* virus-induced gene silencing (VIGS) plants were infected with *P. infestans.* A reduction in lesion sizes was observed in TRV-*NbPRXIIF* VIGS plants compared with TRV-*GFP* plants at 6 dpi. Student’s *t*-test was used for statistical analysis (**, *p* < 0.01; three independent repeats with precise n numbers indicated). **G** and** H** RNA isolation and qRT-PCR results showing the expression levels of alternative oxidase (AOX) genes (**G**) and hormone-related marker genes (**H**) after flg22 treatment. *NbEF1α* was used as the internal control. Results are presented as the mean ± standard error of the mean (SEM) of four repeats (three leaves from different plants were collected together as one repeat). Student’s *t*-test was used for statistical analysis (**, *p* < 0.01; *n* = 4)

To investigate the molecular mechanism by which StNRL-30 suppresses plant defense, a yeast two-hybrid (Y2H) assay was conducted to identify candidate targets of StNRL-30. Several potential StNRL-30 interacting proteins were identified (Table S1). StPRXIIF (Soltu.DM.01G024400.1), which exhibits high similarity with *Arabidopsis* PRXIIF (Fig. S12A), was selected as a candidate target for further investigation. AtPRXIIF is a mitochondria-localized protein that plays a crucial role in preventing oxidative damage and maintaining redox homeostasis (Klupczyńska et al. 2022). To confirm the interaction between StNRL-30 and StPRXIIF, co-IP assays were performed. The results demonstrated that red fluorescent protein (RFP)-StNRL-30 was immunoprecipitated with StPRXIIF-GFP, while RFP-Stphot2 and the EV-RFP control were not immunoprecipitated (Fig. 5A and Fig. S12). LCA confirmed that the combination of StPRXIIF-nLUC with cLUC-StNRL-30 exhibited strong LUC activity (Fig. 5B).

To examine the subcellular localization of StPRXIIF, StPRXIIF-GFP was transiently co-expressed with the mitochondrial marker COX4-mCherry (Xu et al. 2020) in *N. benthamiana* leaves. The results demonstrated that StPRXIIF-GFP co-localized with COX4-mCherry, confirming the mitochondrial localization of StPRXIIF (Fig. 5C). StPRXIIF-GFP showed no colocalization with the PM marker CBL1n-OFP (Fig. S13A and B). Membrane protein fractionation revealed that StNRL-30-GFP was detected in the PM fraction (M), while StPRXIIF-GFP was present in the cytoplasmic fraction (S) (Fig. S13C). BiFC assays were conducted to further determine the interaction site between StNRL-30 and StPRXIIF. YFP fluorescence was detected in regions co-expressing nYFP-StNRL-30 and cYFP-StPRXIIF. The YFP signal showed strong overlap with the PM marker CBL1n and was also observed in cytoplasmic aggregates (Fig. 5D and Fig. S13D). Collectively, these observations established that StNRL-30 interacts with StPRXIIF at both PM and cytoplasmic aggregates.

Transient expression and VIGS were used to investigate the role of StPRXIIF in disease resistance in *N. benthamiana*. Transient expression of StPRXIIF-GFP led to significantly reduced lesions compared with the EV-GFP control (Fig. 5E). The *N. benthamiana* ortholog *NbPRXIIF* exhibits 93.7% identity with *StPRXIIF* (Fig. S12A). TRV-*NbPRXIIF* plants displayed stunted developmental phenotype (Fig. S14A). The transcription level of *NbPRXIIF* decreased by 60–80% in plants expressing TRV-*NbPRXIIF* compared with the TRV-*GFP* control (Fig. S14B). Upon inoculation with *P. infestans*, leaves from the TRV-*NbPRXIIF N. benthamiana* plants exhibited enhanced *P. infestans* colonization compared with the TRV-*GF*P control (Fig. 5F). Thus, StPRXIIF functions as a positive regulator of late blight resistance in *N. benthamiana*.

AOX (alternative oxidase), a mitochondrial stress marker, reduces damage caused by excessive mROS and initiates plant defense responses (Navrot et al. 2007; Wang et al. 2022). The effect of StPRXIIF on the transcript levels of *AOX* genes in *N. benthamiana* after flg22 treatment was examined. The expression levels of *NbAOX1a* and *NbAOX1b* were elevated in leaves transiently expressing StPRXIIF-GFP, compared with leaves expressing EV-GFP control (Fig. 5G). Analysis of pathogen-associated molecular pattern-triggered immunity (PTI) responses and the salicylic acid (SA) and jasmonic acid (JA) signaling pathways revealed that the SA marker genes *NbPR1* and *NbPR2* were upregulated, while the JA marker genes *NbPR3*, *NbPR4*, and *NbPDF1.2* were downregulated in leaves transiently expressing StPRXIIF-GFP (Fig. 5H). No differences were observed in the transcript levels of PTI marker genes *NbACRE31*, *NbWRKY8*, or *NbPti5* (Fig. S14C). Therefore, StPRXIIF enhances plant immunity by upregulating *AOX* gene expression and activating SA signaling pathways, while suppressing JA signaling pathways.

### StNRL-30 mediates StPRXIIF degradation

**Fig. 6:**
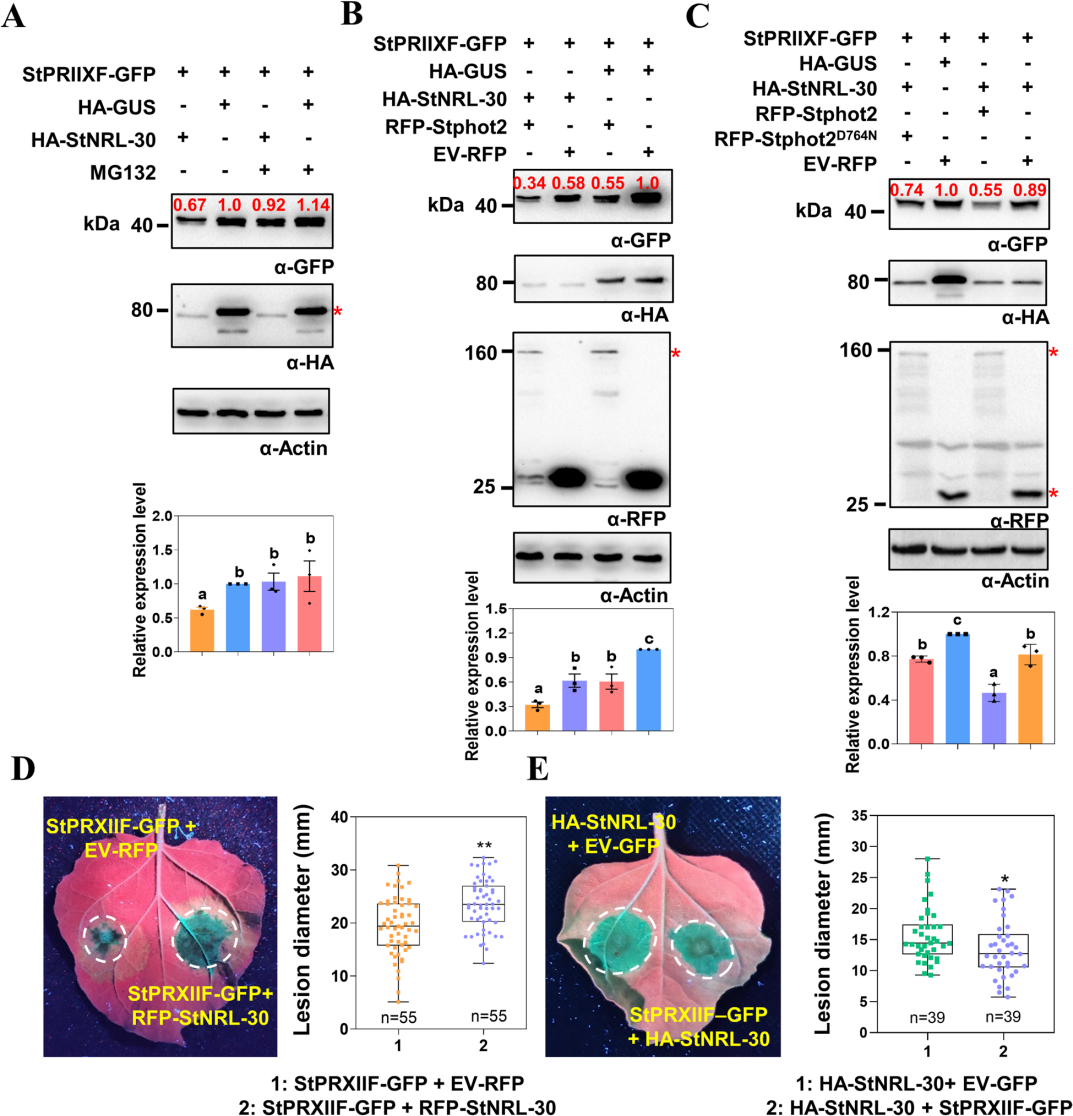
StNRL-30 mediates StPRXIIF degradation. **A** Immunoblot analysis demonstrating that StPRXIIF-GFP protein abundance was significantly reduced in the presence of HA-StNRL-30, with partial recovery following MG132 treatment. Bar graph depicting the relative mean StPRXIIF-GFP band intensities normalized to the control (co-expressed with HA-StNRL-30 without MG132 treatment, assigned a value of 1) from three independent immunoblots. Two additional replicates are shown in Fig. S15A. **B** Immunoblot analysis revealing that the reduction in StPRXIIF-GFP abundance by HA-StNRL-30 was enhanced through co-expression with RFP-Stphot2. Bar graph illustrating the relative mean StPRXIIF-GFP band intensities normalized to the control (co-expressed with EV-RFP and HA-GUS, assigned a value of 1) from three independent immunoblots. Two additional replicates are shown in Fig. S15B. **C** Immunoblot analysis indicating that the reduction in StPRXIIF-GFP abundance was not enhanced by co-expression with RFP-Stphot2^D764N^. Bar graph presenting the relative mean StPRXIIF-GFP band intensities normalized to the control (co-expressed with EV-RFP and HA-GUS, assigned a value of 1) from three independent immunoblots. Two additional replicates are shown in Fig. S15C. Constructs expressed in *N. benthamiana* leaves are indicated by a plus sign (+). Protein sizes are represented in kDa. * indicates target protein bands. In bar graphs of **A**, **B**, and **C**, data are presented as mean ± SEM and one-way ANOVA was used for statistical analysis (different letters indicate significant differences, *p* < 0.05; *n* = 3). **D** and** E** Representative images demonstrating that StNRL-30 suppresses the positive function of StPRXIIF. Box plot displays lesion diameters in *N. benthamiana* leaves at 5 dpi. Dots represent individual data points, and horizontal lines indicate the median. Constructs were agroinfiltrated into *N. benthamiana* leaves. Student’s *t*-test was used for statistical analysis (*, *p* < 0.05; **, *p* < 0.01; three independent repeats with precise n numbers indicated)

The BTB domain containing StNRL-30 functions as a putative ubiquitin E3 ligase, suggesting that StPRXIIF is a substrate for StNRL-30. To test this hypothesis, the StPRXIIF protein levels were examined in *N. benthamiana* when co-expressed with StNRL-30 and with or without treatment of the 26S proteasome inhibitor MG132. The immunoblot analysis revealed that StPRXIIF-GFP abundance significantly decreased in the presence of HA-StNRL-30, compared with the HA-GUS control (Fig. 6A and Fig. S15A). However, MG132 treatment largely prevented StPRXIIF degradation.

Because phots typically function upstream of the NRL family proteins and Stphot2 interacts with StNRL-30 (Fig. 2), the effect of Stphot2 on StPRXIIF protein abundance was investigated. StPRXIIF-GFP was co-expressed with several combinations: EV-RFP + HA-GUS; RFP-Stphot2 + HA-StNRL-30; RFP-Stphot2 + HA-GUS; EV-RFP + HA-StNRL-30. StPRXIIF-GFP abundance decreased when co-expressed with HA-StNRL-30 + EV-RFP, compared with the control HA-GUS + EV-RFP. Similarly, co-expression with RFP-Stphot2 + HA-GUS reduced StPRXIIF-GFP abundance. Moreover, StPRXIIF-GFP abundance significantly decreased when co-expressed with HA-StNRL-30 + RFP-Stphot2 (Fig. 6B and Fig. S15B). These findings indicate that StNRL-30, in conjunction with Stphot2, mediates proteasome-dependent StPRXIIF degradation.

The kinase activity of phots is essential for phot-mediated physiological responses (Inoue et al. 2008, 2011). An examination was conducted to assess the effect of the Stphot2^D764N^ mutant on StPRXIIF degradation. StPRXIIF-GFP was co-expressed with several combinations: EV-RFP + HA-GUS; RFP-Stphot2 + HA-StNRL-30; RFP-Stphot2^D764N^ + HA-StNRL-30; EV-RFP + HA-StNRL-30. The StPRXIIF-GFP protein level decreased when co-expressed with EV-RFP + HA-StNRL-30, and the combination RFP-Stphot2 + HA-StNRL-30 induced a significant reduction in the StPRXIIF-GFP protein level. However, RFP-Stphot2^D764N^ + HA-StNRL-30 did not substantially reduce StPRXIIF-GFP abundance compared with RFP-Stphot2 + HA-StNRL-30 (Fig. 6C and Fig. S15C).

To investigate whether StNRL-30 influences StPRXIIF-mediated resistance to *P. infestans*, StPRXIIF-GFP was transiently co-expressed with RFP-StNRL-30 or EV-RFP in *N. benthamiana* leaves. The results demonstrated that StNRL-30 suppressed the positive function of StPRXIIF, as evidenced by significantly increased lesion diameters in the presence of RFP-StNRL-30 compared with the EV-RFP control (Fig. 6D). In addition, when HA-StNRL-30 was transiently co-expressed with StPRXIIF-GFP or EV-RFP, the results indicated that the expression of StPRXIIF-GFP counteracted HA-StNRL-30-mediated susceptibility to *P. infestans* compared with the EV-GFP control (Fig. 6E). These findings reveal that StNRL-30 suppresses resistance triggered by StPRXIIF.

### BL promotes StPRXIIF degradation and re-localizes the StNRL-30–StPRXIIF complex from the PM to the chloroplasts

**Fig. 7:**
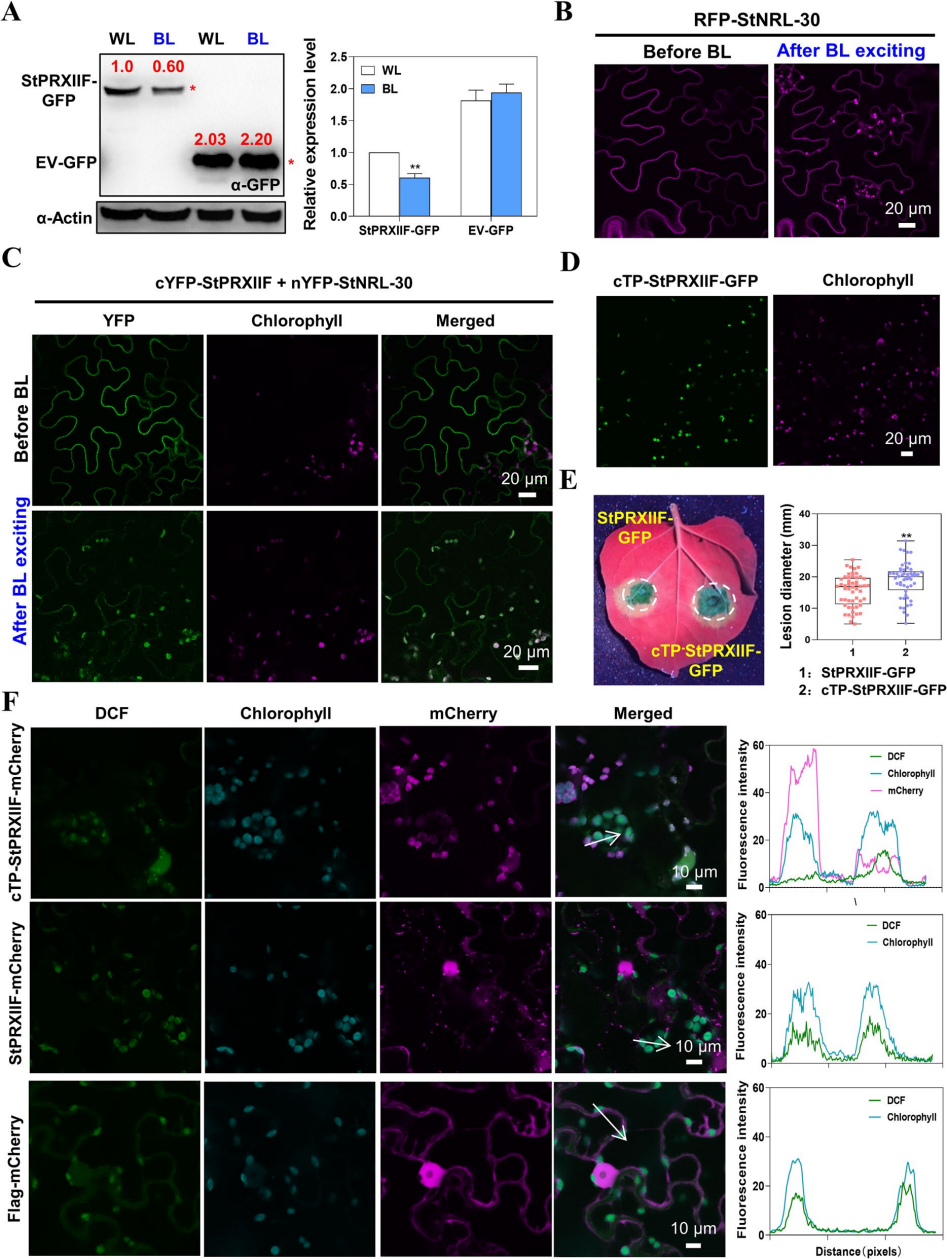
Blue light (BL) re-localizes the StNRL-30–StPRXIIF complex and promotes StPRXIIF turnover. **A** Immunoblot analysis demonstrating that StPRXIIF-GFP abundance decreased after BL treatment. Agroinfiltrated *N. benthamiana* plants were grown under BL or white light (WL) conditions. * indicates target protein band. Bar graph illustrates the relative StPRXIIF-GFP abundance from three biological replicates (each containing four leaves). Student’s *t*-test was used for statistical analysis (**, *p* < 0.01; *n* = 3). **B** Confocal images demonstrate the localization of RFP-StNRL-30 under BL. Freshly injected plants were maintained under constant light for 30 h, then transferred to darkness for 18 h (dark adaptation). After initial scanning (before BL), samples were exposed to 488 nm excitation for 6 min (after BL). **C** Confocal images reveal the localization of nYFP-StNRL-30 and cYFP-StPRXIIF under BL. **D** Subcellular localization of cTP-StPRXIIF-GFP in *N. benthamiana*. Confocal images were acquired at 48 hpa. **E** Representative leaf images and box plots indicating lesion diameters in *N. benthamiana* leaves infiltrated with cTP-StPRXIIF-GFP or StPRXIIF-GFP at 5 dpi. Dots represent individual data points, and horizontal lines indicate the median. Student’s *t*-test was used for statistical analysis (**, *p* < 0.01; *n* = 52). **F** Expression of StPRXIIF-GFP in chloroplasts suppresses ROS accumulation activated by flg22. At 48 hpa, leaves were infiltrated with DCF, placed in darkness for 30 min, treated with flg22 to activate ROS burst, and observed 10 min later. DCF (green fluorescence) indicates ROS signal. Arrows indicate paths used for generating the fluorescence intensity profiles. Plots on the right show the intensity of green fluorescence and chlorophyll emission fluorescence

To examine whether BL triggers StPRXIIF degradation, StPRXIIF-GFP and EV-GFP were transiently expressed in *N. benthamiana* under BL and WL conditions, respectively. StPRXIIF-GFP protein abundance decreased under BL compared with WL conditions, while EV-GFP abundance remained unchanged (Fig. 7A and Fig. S16A), indicating BL induces StPRXIIF degradation.

BL triggers NPH3 release from the PM into the cytoplasmic condensate, a process considered fundamental for its normal function (Reuter et al. 2021; Sullivan et al. 2021; Waksman et al. 2023). StNRL-30 initially localized predominantly to the PM, but substantially re-localized to the chloroplasts upon BL (6 min 488 nm laser) treatment (Fig. 7B). StPRXIIF-GFP did not re-localize to the chloroplasts upon BL irradiation (Fig. S16B). BiFC assay was conducted to investigate whether BL triggers the re-localization of the StNRL-30–StPRXIIF complex. Confocal microscopy revealed that the StNRL-30–StPRXIIF complex transferred from the PM to the chloroplasts after BL (6 min 488 nm laser) excitation (Fig. 7C and Fig. S16C). Some of the complex was also observed in the cytoplasm (Fig. S16C). These findings demonstrate that BL triggers the re-localization of StNRL-30 and the StNRL-30–StPRXIIF complex from the PM to the chloroplasts.

To examine whether the re-localization of StPRXIIF to the chloroplasts affects its ability to suppress *P. infestans* colonization in *N. benthamiana* leaves, a chloroplast transit peptide (cTP)-fusion mutant cTP-StPRXIIF-GFP was generated. Confocal microscopy revealed that cTP-StPRXIIF-GFP was exclusively localized to the chloroplasts (Fig. 7D and Fig. S17A). Immunoblotting confirmed that cTP-StPRXIIF-GFP was stably expressed in *N. benthamiana* (Fig. S17B). Transient expression of cTP-StPRXIIF-GFP in *N. benthamiana* resulted in larger late blight lesions compared with expression of StPRXIIF-GFP (Fig. 7E), indicating that the chloroplast-localized StPRXIIF lost its suppressive function against *P. infestans*. ROS serves as a key immune signal of chloroplasts. To investigate whether chloroplast-localized StPRXIIF interferes with chloroplast ROS (cROS) accumulation, cTP-StPRXIIF-mCherry, StPRXIIF-mCherry, and Flag-mCherry were transiently expressed in *N. benthamiana* leaves, after which ROS accumulation was monitored using a fluorescent indicator, 2’7’-dichlorodihydrofluorescein diacetate (DCF-DA). The cROS signal was reduced in cells expressing cTP-StPRXIIF-mCherry compared with cells expressing StPRXIIF-mCherry or the control Flag-mCherry (Fig. 7F), indicating that cROS production was disrupted when StPRXIIF was translocated to the chloroplasts. Immunoblotting confirmed that cTP-StPRXIIF-mCherry, StPRXIIF-mCherry, and Flag-mCherry were stably expressed in *N. benthamiana* (Fig. S17C). Collectively, these results suggest that BL and StNRL-30 induce re-localization and StPRXIIF degradation, thereby subverting its positive regulatory effects in plant immunity.

## Discussion

Both Stphot1 and Stphot2 negatively regulate late blight resistance, and Stphot1 suppresses the ICD immune response through a NRL protein StNRL1, while Stphot2 does not (Naqvi et al. 2022), suggesting Stphot1 and Stphot2 enhance plant susceptibility through distinct mechanisms. This study reveals that Stphot2 interacted with another NRL protein StNRL-30, which also functions as a susceptibility factor. Consistent with Stphot2, StNRL-30 did not suppress the ICD immune response (Fig. 2). Thus, Stphot2 and StNRL-30 may suppress alternative immune responses in *N. benthamiana*, such as cell death elicited by various microbe-associated molecular patterns or different RESISTANCE (R)/AVIRULENCE (AVR) combinations. Plant chloroplasts and stomata serve as crucial immune organelles coordinating plant defense responses and are frequently targeted by pathogens (Caplan et al. 2015; Hou et al. 2024; Liu et al. 2024). Notably, phot2 regulates BL-induced chloroplast movement and stomatal opening in *Arabidopsis*. Further investigation is warranted to determine whether Stphot2 regulates chloroplast movement and thereby interferes with immune signal activation, whether Stphot2 promotes stomatal opening contributing to *P. infestans* colonization, and whether StNRL-30 regulates physiological processes controlled by Stphot2 during infection.

Phots directly phosphorylate NPH3 and RPT2 at the conserved C-terminal consensus sequences (RxSΦS), which function as 14-3-3 protein-binding motifs (Reuter et al. 2021; Sullivan et al. 2021; Waksman et al. 2023). Both phosphorylation of Ser residues in the RxSΦS motif and 14-3-3 binding are essential for NPH3 and RPT2 biological functions. This study revealed that StNRL-30 contains the conserved RxSΦS motif at the C-terminus (Fig. S8), and both Stphot2 and StNRL-30 interacted with St14-3-3 (Fig. S10). Mutation of potential phosphorylation sites in the RxSΦS motif of StNRL-30 diminished its interaction with Stphot2 and St14-3-3, significantly reducing its capacity to enhance *P. infestans* colonization in *N. benthamiana* (Fig. 4 and Fig. S10C). In addition, the interaction between Stphot2 and StNRL-30 weakened when the Stphot2 kinase domain was mutated (Fig. 4). AlphaFold3 predictions indicate that Ser627 and Ser629 of StNRL-30 serve as interaction sites with 14-3-3 protein, with Ser627 functioning as the phosphorylation site (Fig. S11). These observations support the hypothesis that phosphorylation and subsequent 14-3-3 binding on the C-terminal RxSΦS motif of NRL proteins constitute conserved regulation mechanisms exerted by phots. Future research will investigate whether Stphot2 directly phosphorylates StNRL-30 at the C-terminal RxSΦS motif, and whether BL and Stphot2 kinase activity are fundamental for StNRL-30 activation.

BTB domain-containing proteins function as substrate-specific adapters of CULLIN3 (CUL3)-based E3 ubiquitin ligase complexes (CRL3s) to recruit target proteins (Genschik et al. 2013; Krek 2003; Willems et al. 2004). *Arabidopsis* NPH3 acts as a substrate adapter in the CRL3^NPH3^ complex, targeting phot1 for ubiquitination (Roberts et al. 2011). AtSR1IP1 (also known as AtNCH1) functions as a substrate adapter in the CRL3 complex, mediating degradation and ubiquitination of the CaM-binding transcription factor AtSR1 by the 26S proteasome system (Zhang et al. 2014). This study demonstrates that StNRL-30 forms a homodimer, requiring conserved pocket residues in the BTB domain. StNRL-30^N/Q^, a conserved pocket residue mutant, failed to enhance *P. infestans* infection (Fig. 4). StNRL-30 interacted with StPRXIIF (Fig. 5) and promoted its degradation (Fig. 6). However, the precise mechanism by which StNRL-30 mediates StPRXIIF degradation remains unclear. StNRL-30 potentially forms a ubiquitin E3 ligase complex with CUL3 to recruit StPRXIIF for ubiquitination and subsequent degradation by the 26S proteasome system. Further investigation will determine whether StNRL-30 interacts with CUL3, and whether the observed connection between StNRL-30 and StPRXIIF depends on the BTB domain or CUL3.

Plant PRXs participate in numerous redox regulatory pathways in eukaryotes and influence cellular signal transduction (Liebthal et al. 2018). However, PRXs’ role in pathogen response remains largely unexplored. This study revealed that StPRXIIF positively regulated late blight resistance in *N. benthamiana* (Fig. 5). StPRXIIF overexpression enhanced the expression of mitochondrial *AOX* genes. In poplar plants, elevated *AOX* levels enhance resistance against *Marssonina brunnea* (Liao et al. 2020). AOX also participates in hormone signaling pathways. High SA concentration enhances plant resistance and upregulates *AOX1a* expression (Nie et al. 2015). Exogenous JA and ethylene treatment enhanced the mitochondrial AOX pathway, inducing poplar resistance to *M. brunnea* (Liao et al. 2020). In this study, we found that *StPRXIIF* overexpression upregulated SA pathway marker genes while downregulating JA pathway genes (Fig. 5), suggesting that StPRXIIF-triggered resistance is related to hormone signaling pathways. Future research will explore whether StPRXIIF-induced changes in *AOX* expression associate with hormone signaling pathways. Mitochondrial retrograde signaling facilitates communication from the mitochondria to the nucleus, altering nuclear gene expression (Wang et al. 2022). Notably, *AOX* serves as a marker gene for mitochondrial retrograde signaling activation (Ng et al. 2013). Future studies will elucidate whether StPRXIIF transmits immune signals to the nucleus by mitochondrial retrograde signaling to activate defense-related genes.

BL induces the re-localization of NPH3 from the PM into cytosolic aggregates, accompanied by changes in NPH3 phosphorylation status (Reuter et al. 2021; Sullivan et al. 2021). Recently, NRL5 has been reported to be a trafficking-associated GTPase, co-localizing with RABE1c GTPases and vesicle-associated membrane protein 721 (Upadhyay-Tiwari et al. 2024; Verslues and Upadhyay-Tiwari 2024). In this study, StNRL-30 was found to localize in the PM during initial 488 nm scanning (which excites GFP and activates phots), before translocating from the PM to chloroplasts after 6 min of 488 nm excitation (Fig. 7). Furthermore, BL irradiation induced substantial re-localization of the StNRL-30–StPRXIIF complex from the PM to chloroplasts (Fig. 7). BL not only triggered StPRXIIF degradation but also induced the re-localization of the StNRL-30–StPRXIIF complex (Fig. 7). StNRL-30-mediated 26S proteasome degradation of StPRXIIF may occur during this cytoplasmic re-localization process.

Chloroplasts serve as crucial mediators in activating defensive responses during plant–pathogen interactions (Littlejohn et al. 2021). The re-localization of defense-related proteins commonly occurs during such interactions. For instance, *Arabidopsis* calcium protein kinase 16 relocates from the PM to chloroplasts upon flg22 treatment to enhance chloroplast-mediated defenses (Medina-Puche et al. 2020). The C4 protein from tomato yellow leaf curl virus alters its localization from the PM to chloroplasts upon defense activation and suppresses chloroplast-mediated defenses (Rosas-Diaz et al. 2018; Medina-Puche et al. 2020). Glycerate kinase (GLYK) produces two distinct transcripts in response to light changes, with full-length StGLYK^FL^ and shorter StGLYK^cyt^ accumulating in chloroplasts and the cytoplasm, respectively (Gao et al. 2020). GLYK functions as a positive immunoregulator in a full length-dependent manner. This study demonstrates that chloroplast transit peptide (cTP) in cTP-StPRXIIF undermines its role as a positive immunoregulator in chloroplasts (Fig. 7). cROS plays an essential role in establishing plant immunity (Littlejohn et al. 2021). The results indicated that the presence of cTP-StPRXIIF suppressed ROS production in chloroplasts (Fig. 7). Thus, the re-localization of StPRXIIF to chloroplasts potentially reduces cROS and alters the chloroplast redox state, thereby affecting immune signaling.

Both StPRXIIF translocation and degradation contribute to compromising late blight resistance. The chloroplast re-localization of StPRXIIF directed by cTP increased susceptibility and reduced cROS accumulation (Fig. 7). BL promotes both StPRXIIF degradation and re-localization (Fig. 7). Under natural light conditions, while StPRXIIF re-localization rarely occurs, StNRL-30-mediated StPRXIIF degradation persists. Given StPRXIIF’s crucial role in mitochondria, its degradation likely occurs during cytoplasmic translocation, playing a primary role in compromising late blight resistance.

**Fig. 8:**
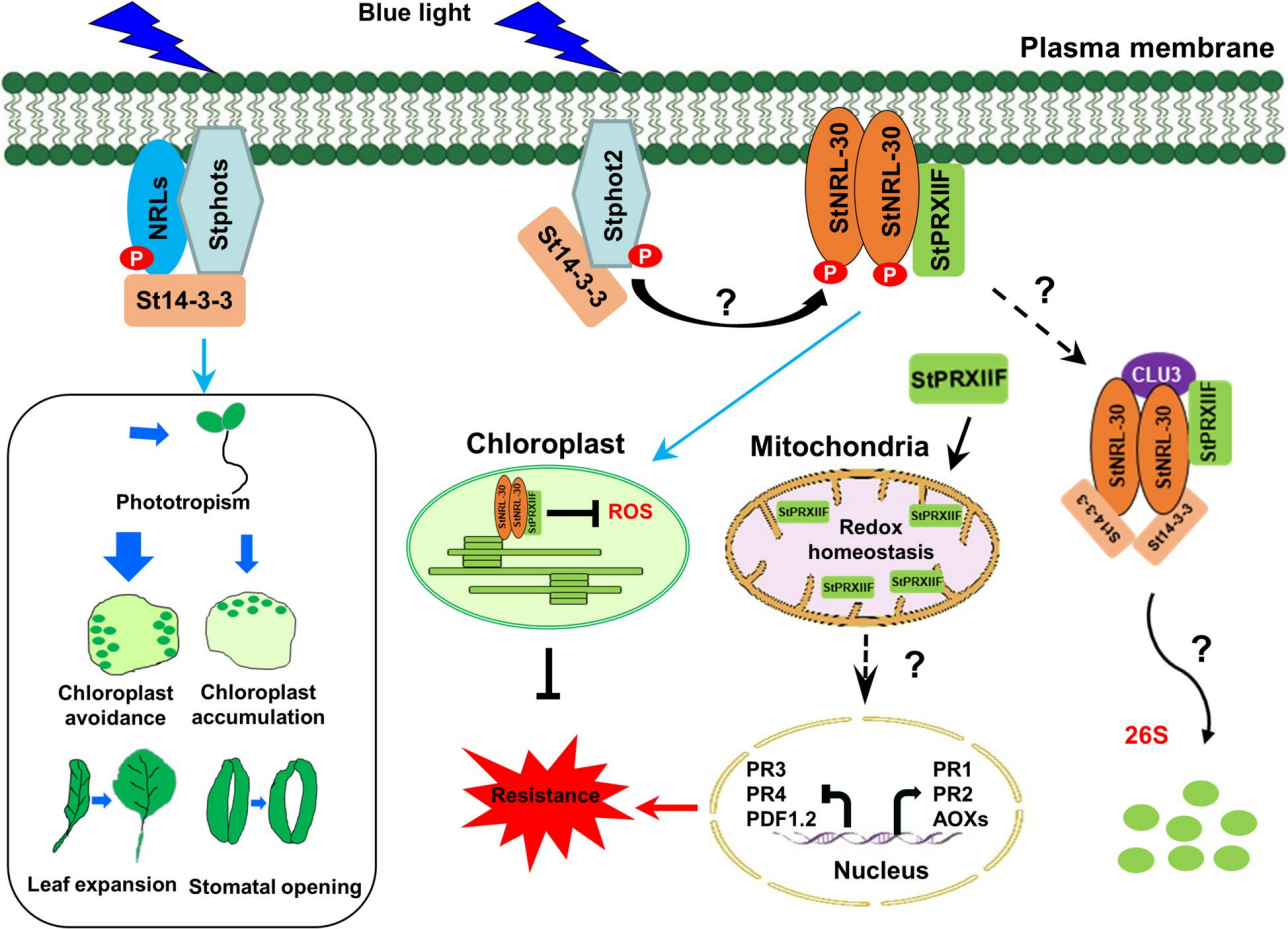
Proposed model of Stphot2 collaborating with StNRL-30 to negatively regulate immunity. Upon BL excitation, Stphot2 undergoes rapid auto-phosphorylation and subsequently activates downstream NRL proteins, including StNRL-30, through phosphorylation. Certain NRLs may participate in chloroplast avoidance and accumulation processes (Christie 2007). StNRL-30 potentially functions as a substrate adapter of the Cullin-RING ligase complex (Roberts et al. 2011), recruiting StPRXIIF for degradation in the cytoplasm. In addition, BL initiates the re-localization of the StNRL-30–StPRXIIF complex from the PM to the chloroplasts. StPRXIIF maintains redox homeostasis in mitochondria and the hormone signaling pathway, contributing positively to plant resistance. The degradation and re-localization of StPRXIIF diminish its positive role in plant immunity

A proposed model suggests the following mechanism (Fig. 8). BL perception triggers Stphot2 autophosphorylation, which directly activates NRL family members, potentially regulating various physiological responses including phototropism, chloroplast movement, stomatal opening, and leaf expansion (Christie 2007; Christie et al. 2018). Simultaneously, auto-phosphorylated Stphot2 directly phosphorylates and activates StNRL-30, leading to StPRXIIF recruitment and subsequent degradation by the 26S proteasome system. In addition, BL induces the re-localization of the StNRL-30–StPRXIIF complex to chloroplasts, disrupting StPRXIIF’s positive role in immunity regulation.

This research elucidates how the BL receptor Stphot2 regulates late blight resistance through downstream NRL proteins. The study provides evidence connecting BL signaling with plant immunity, advancing understanding of the interaction between BL perception through photoreceptors and plant immunity, while suggesting new approaches for enhancing disease resistance through light spectrum management.

## Materials and methods

### Plant materials and growth conditions

Potato (*Solanum tuberosum* L.) plants were cultivated in a glasshouse under natural environmental conditions. Leaves from six- to eight-week-old potato plants were used for *P. infestans* infection assays. *N. benthamiana* plants were grown in a growth chamber at 22 °C under 16 h/8 h (light/dark) photoperiod with 70% relative humidity. Three- to four-week-old *N. benthamiana* plants were used for transient expression assays.

### Plasmid constructs

*Stphot2*, *Stphot1*, *Stphot2*^*D764N*^, *StNRL-30*, *StNRL-30*^*A/A*^, *StNRL-30*^*N/Q*^, and *StNRL1* were inserted into pH7lic-NGFP (N-terminal GFP tag) and pH7lic-NHA (N-terminal 3 × HA tag). *Stphot2*, *Stphot2*^*D764N*^, and *StNRL-30* were inserted into pK7WGR2 (N-terminal RFP tag). *StPRXIIF* was inserted into pH7lic-CGFP (C-terminal GFP tag) and pH7lic-CHA (C-terminal 3 × HA tag). Specific fragments from *Stphot2*, *Stphot1*, and *StNRL-30* (Table S2) were inserted into pHELLSGATE8 to generate RNAi constructs. For VIGS assay, specific fragments from *Nbphot2*, *Nbphot1*, *NbNRL-30*, and *NbPRXIIF* (Table S2) were inserted into TRV2. For split LCA, *Stphot2* and *StPRXIIF* were inserted into pCAMBIA1300-nLUC with a 3 × HA or GFP tag, while StNRLs and NRL-30 mutants were inserted into pCAMBIA1300-cLUC with a 3 × HA or GFP tag. For split-YFP constructs, *Stphot2*, *StNRL-30*, *Stphot1*, and *StPRXIIF* were inserted into the N-terminal of YFP (nYFP) with a 3 × Myc tag and C terminal cYFP with a 3 × HA tag. All constructs were generated using the ClonExpress^®^ II One Step Cloning Kit (Cat no. C112-02; Vazyme, Nanjing, China). For Y2H assay, the coding sequence of *StNRL-30* was inserted into pDONR221 with gene-specific primers modified to contain the Gateway^®^ attB recombination sites. It was recombined with pDEST32 (prey vector) to generate pDEST32-StNRL-30 with LR clonase (Cat no. 2273391; Invitrogen, Carlsbad, CA, USA). All vectors are listed in Table S3. All primers used in this study are listed in Table S4.

### *Agrobacterium*-mediated transient expression assays

*Agrobacterium tumefaciens* strain GV3101 containing the corresponding constructs were cultured overnight in yeast extract broth with appropriate antibiotics at 28 °C. *Agrobacterium* cells were centrifuged and resuspended in MMA buffer (10 mM MES, 10 mM MgCl_2_, and 200 mM acetosyringone; pH = 5.6), and adjusted to the required OD_600_ value prior to infiltration into *N. benthamiana* leaves (0.5 for Western blot, LCA, cell death assay; 0.05–0.1 for confocal imaging; 0.1 for *P. infestans* infection assay; and 0.3 for BiFC assay).

### TRV-based VIGS in *N. benthamiana*

VIGS constructs were generated by cloning an approximately 300-bp PCR fragment of the target genes for silencing. A tobacco rattle virus (TRV) construct expressing *GFP* served as a control. *Agrobacterium* strains containing the TRV1 construct were resuspended in MMA buffer at a final concentration of OD_600_ = 0.4, with TRV2 constructs at a final concentration of OD_600_ = 0.5. The two largest leaves of four-leaf-stage *N. benthamiana* plants were fully infiltrated with the *Agrobacterium* mixture. The treated plants were used for assays 2–3 weeks later.

### Potato transformation

OE constructs (35S::*GFP-Stphot2*, 35S::*GFP-StNRL-30*, and 35S::*GFP-Stphot1*) and RNAi constructs (*Stphot2*-Ri, *StNRL-30*-Ri, and *Stphot1*-Ri) were introduced into *A. tumefaciens* strain GV3101. Subsequently, they were transformed into potato variety ‘E-potato 3’ (‘E3’) as described previously (Guo et al. 2022).

### Confocal microscopy

For subcellular localization observation, confocal images of leaf cells expressing the protein fusions at 48 h post-agroinfiltration (hpa) were captured using a confocal laser scanning microscope (Leica SP8, Wetzlar, Germany). A wavelength of 488 nm was used for GFP, YFP, or DCF excitation, and the emission signal of the wavelength (496–533 nm) was obtained for the GFP, YFP, or DCF channel. A wavelength of 552 nm was used for RFP, mCherry, or OFP, and the emission signal of the wavelength (568–610 nm) was obtained for the RFP, mCherry, or OFP channel. Chloroplast autofluorescence was captured between 650 and 690 nm in emission and 630 nm in excitation.

### *P. infestans* inoculation assay

*P. infestans* isolates 88069 and HB09-14-2 were cultured in Rye-Suc-Agar medium at 19 °C for two weeks under dark conditions. The sporangia were collected from medium plates and suspended in double distilled water, stored at 4 °C for 2–8 h, and used for inoculation. For *N. benthamiana* leaf inoculation, the sporangia concentration of isolate 88069 was adjusted to 15,000 sporangia∙mL^−1^. For potato leaf inoculation, the sporangia concentration of isolate HB09-14-2 was adjusted to 12,000 sporangia∙mL^−1^. Subsequently, 10-μL droplets were inoculated onto the abaxial surface of detached leaves of *N. benthamiana* or potato. Lesion diameters were measured at 5–7 days post-inoculation.

### Trypan blue staining assay

For trypan blue staining, the detached leaves were immersed in 1 mg∙mL^−1^ trypan blue buffer at room temperature overnight, then cleared with 95% ethanol, and boiled until complete decolorization.

### Membrane protein fractionation

PM proteins of *N. benthamiana* leaves were extracted using the Minute^TM^ Plasma Membrane Protein Isolation Kit (Cat no. SM-005; Invent Biotechnologies, Beijing, China).

### cROS detection

2’ 7’-dichlorodihydrofluorescein (DCF-DA) was used to measure ROS production levels in chloroplasts. DCF oxidation generates green fluorescence in chloroplasts. Following 48-h transient expression of cTP-StPRXIIF-mCherry, StPRXIIF-mCherry, and Flag-mCherry in *N. benthamiana*, the leaves underwent infiltration with 10 μM DCF and exposure to darkness for 30 min. Subsequently, these leaves received treatment with 10 μM flg22. A confocal microscope measured fluorescence intensity as described by Qi et al. (2024).

### Split LCA

The corresponding combination of cLUC/nLUC constructs were co-expressed in *N. benthamiana*. nLUC-EV (Myc-nLUC) and cLUC-EV (cLUC-HA or cLUC-GFP) served as control vectors containing initiation and stop codons. Two days after infiltration, 15 mM of LUC substrate luciferin was applied to the detached leaves. Fluorescence detection occurred 10 min after luciferin treatment, with images captured using the NightShade LB 985 In Vivo Plant Imaging System (Germany).

### BiFC assay

The corresponding combination of nYFP/cYFP constructs were co-expressed in *N. benthamiana* leaves. At 48 hpa, fluorescence signals were observed and images were captured using a confocal laser scanning microscope (Leica SP8).

### Western blot analysis

Leaf samples were collected 48 hpa, and proteins were extracted using GTEN [10% (v/v) glycerol, 25 mM Tris·HCl (pH 7.5), 1 mM EDTA, 150 mM NaCl] buffer with 10 mM dithiothreitol (DTT), 1 × protease inhibitor cocktail (Roche), 1 mM phenylmethyl sulphonyl fluoride (PMSF), and 0.2% Nonidet P-40 (NP40). The samples were mixed with 2 × sodium dodecyl sulfate (SDS) sample buffer (100 mM Tris-HCl, 4% SDS, 20% glycerol, 0.2% bromophenol blue, 200 mM DTT) and heated at 95 °C for 10 min. Samples underwent separation on 10% SDS-PAGE gels and were transferred onto polyvinylidene fluoride membranes for confirmation by Ponceau staining (PS). Membranes were blocked in 5% milk in 1 × phosphate-buffered saline (with 0.1% Tween) with shaking for 1–2 h at room temperature, then incubated with corresponding antibodies (polyclonal anti-GFP and anti-HA; Bioyears, Wuhan, China) at 1:4,000. Anti-mouse polyclonal HRP-conjugated antibody (Immunoway, RS0001) was applied at 1:4,000 as a secondary antibody for GFP and HA. Protein sizes were indicated by 180 kDa Prestained Protein Marker (Cat no. 20350; Yeasen, Shanghai, China). The immunoblots were visualized using the ChemiDoc XRS+ Gel Imaging System (Bio-Rad, Hercules, CA, USA) after treatment with chemiluminescent reagents (Servicebio, Wuhan, China).

### Co-IP assay

Leaf samples were collected 48 hpa, and proteins were extracted using GTEN buffer with 10 mM DTT, 1 × protease inhibitor cocktail, 1 mM PMSF, and 0.2% NP40. Twenty microliters of prewashed anti-GFP or anti-HA agarose beads (Cat no. KTSM1301, KTSM1306; AlpalifeBio, Shenzhen, China) were added to 400 μL protein extracts and incubated for 2 h at 4 °C on a rocking platform at low speed. Following incubation, the beads underwent four washes with 800-μL wash buffer (GTEN buffer with 1 mM PMSF and 1 × protease inhibitor cocktail) and were heated at 95 °C for 10 min in 2 × SDS loading buffer supplemented with 200 mM DTT.

### RNA isolation and qRT-PCR

Total RNA was extracted using the Plant Total RNA Kit (Cat no. ZP405-1; Zomanbio, Beijing, China) following the manufacturer’s protocol. RNA (2 µg) was used for first-strand cDNA synthesis using the All-In-One 5 × RT MasterMix Kit (Cat no. G592; Applied Biological Materials, Richmond, BC, Canada) according to the manufacturer’s specifications. qRT-PCR was performed using EvaGreen 2 × qPCR MasterMix (Cat no. G891; Applied Biological Materials) on an ABI7300 PCR machine (Applied Biosystems, Foster, CA, USA). Gene expression levels were determined using the 2^−ΔΔCT^ method (Livak and Schmittgen 2001) or comparative Ct method (Čikoš et al. 2007). qRT-PCR primers are listed in Table S4.

### Y2H assay

Y2H screening with pDEST32-StNRL-30 was performed in *Saccharomyces cerevisiae* strain MaV203 as described (McLellan et al. 2013) using the Invitrogen ProQuest system.

### Statistical analyses

Statistical analyses were performed using one-way analysis of variance or Student’s *t*-test and pairwise or multiple comparisons with GraphPad Prism 8.0 software. All values and error bars represent mean ± standard error of the mean of three or more replicates. Quantitative analysis of proteins was performed using ImageJ software.

## Supplementary Information

Supplementary Material 1: Fig. S1. Silencing of either *Nbphot1* or *Nbphot2* resulted in a significant reduction in *Phytophthora* *infestans* colonization. Fig. S2. Gene expression levels and plant phenotype of representative *Stphot2*-overexpression (OE) and -RNAi (Ri) transgenic potato lines. Fig. S3. *Stphot1* negatively regulates potato late blight resistance. Fig. S4. Blue light negatively regulates potato late blight resistance. Fig. S5. Immunoblots showing that GFP-Stphot2 and GFP-Stphot2^D764N^ are stably expressed in *Nicotiana benthamiana* leaves. Fig. S6. Split luciferase complementation assay (LCA) identified NRLs interacting with Stphot2. Fig. S7. Subcellular localization of StNRL-30. Fig. S8. Alignment of NRL-30 sequences. Fig. S9. *StNRL-30* expression levels and plant growth phenotypes of virus-induced *NbNRL-30*-silenced* N. benthamiana *plants and *StNRL-30*-OE and -Ri potato lines. Fig. S10. St14-3-3 interacts with both StNRL-30 and Stphot2. Fig. S11. Predicted phosphorylation and 14-3-3 binding sites on StNRL-30. Fig. S12. StNRL-30 interacts with StPRXIIF. Fig. S13. StPRXIIF is located in the cytoplasm. Fig. S14. Silencing levels and plant phenotypes of virus-induced *PRXIIF*-silenced* N. benthamiana* plants. Fig. S15. Degradation of StPRXIIF mediated by StNRL-30 and Stphot2. Fig. S16. Blue light affects stability of StPRXIIF and localization of the *StNRL-*30–StPRXIIF complex. Fig. S17. cTP-StPRXIIF-GFP is stably expressed in *N. benthamiana*.

Supplementary Material 2: Table S1. Putative interaction proteins identified by Y2H library screening. Table S2. Sequence of gene fragments used in silencing constructs. Table S3. Vectors used in this study. Table S4. Primers used in this study.

## Data Availability

All data supporting the findings of this study are available in the main text and its Supporting Information files.

## Abbreviations

BL: Blue light
WL: White light
NRL: NPH3/RPT2-like
Phots: Phototropins
SA: Salicylic acid
JA: Jasmonic acid
ICD: INF1 induced cell death
AOX: Alternative oxidase
PRXs: Peroxiredoxins
LCA: Luciferase complementation assay
Co-IP: Co-immunoprecipitation
qRT-PCR: Quantitative real-time PCR
Y2H: Yeast-two-hybrid
BiFC: Bimolecular fluorescence complementation

## References

[CR1] Caplan JL, Kumar AS, Park E, Padmanabhan MS, Hoban K, Modla S, et al. Chloroplast stromules function during innate immunity. Dev Cell. 2015;34:45–57. 26120031 10.1016/j.devcel.2015.05.011 PMC4596411

[CR2] Cheng D, Qiu H, Zhou D, Lin T, Liu L, Nie J, et al. Genome-wide identification and characterization of potato NRL gene family and functional analysis of StNRL-6 in response to *Phytophthora infestans*. Physiol Plantarum. 2024;176:e14650. 10.1111/ppl.14650 39632458

[CR3] Christie JM. Phototropin blue-light receptors. Ann Rev Plant Biol. 2007;58:21–45. 17067285 10.1146/annurev.arplant.58.032806.103951

[CR4] Christie JM, Salomon M, Nozue K, Wada M, Briggs WR. LOV (light, oxygen, or voltage) domains of the blue-light photoreceptor phototropin (nph1): Binding sites for the chromophore flavin mononucleotide. Proc Natl Acad Sci USA. 1999;96:8779–83. 10411952 10.1073/pnas.96.15.8779 PMC17593

[CR5] Christie JM, Yang H, Richter GL, Sullivan S, Thomson CE, Lin J, et al. Phot1 inhibition of ABCB19 primes lateral auxin fluxes in the shoot apex required for phototropism. PLoS Biol. 2011;9:e1001076. 21666806 10.1371/journal.pbio.1001076 PMC3110179

[CR6] Christie JM, Blackwood L, Petersen J, Sullivan S. Plant flavoprotein photoreceptors. Plant Cell Physiol. 2015;56:401–13. 25516569 10.1093/pcp/pcu196 PMC4357641

[CR7] Christie JM, Suetsugu N, Sullivan S, Wada M. Shining light on the function of NPH3/RPT2-like proteins in phototropin signaling. Plant Physiol. 2018;176:1015–24. 28720608 10.1104/pp.17.00835 PMC5813532

[CR8] Čikoš Š, Bukovská A, Koppel J. Relative quantification of mRNA: comparison of methods currently used for real-time PCR data analysis. BMC Molecular Biology. 2007;8:113. 18093344 10.1186/1471-2199-8-113 PMC2235892

[CR9] Courbier S, Grevink S, Sluijs E, Bonhomme PO, Kajala K, Van Wees SCM, et al. Far-red light promotes *Botrytis cinerea* disease development in tomato leaves via jasmonate-dependent modulation of soluble sugars. Plant Cell Environ. 2020;43:2769–81. 32833234 10.1111/pce.13870 PMC7693051

[CR10] Courbier S, Snoek BL, Kajala K, Li L, Van Wees SCM, Pierik R. Mechanisms of far-red light-mediated dampening of defense against *Botrytis cinerea* in tomato leaves. Plant Physiol. 2021;187:1250–66. 34618050 10.1093/plphys/kiab354 PMC8566310

[CR11] Cvetkovska M, Vanlerberghe GC. Alternative oxidase impacts the plant response to biotic stress by influencing the mitochondrial generation of reactive oxygen species. Plant Cell Environ. 2012;36:721–32. 22978428 10.1111/pce.12009

[CR12] Demarsy E, Schepens I, Okajima K, Hersch M, Bergmann S, Christie J, et al. Phytochrome kinase substrate 4 is phosphorylated by the phototropin 1 photoreceptor. EMBO J. 2012;31:3457–67. 22781128 10.1038/emboj.2012.186 PMC3419926

[CR13] Demkura PV, Ballare CL. UVR8 mediates UV-B-induced Arabidopsis defense responses against *Botrytis cinerea* by controlling sinapate accumulation. Mol Plant. 2012;5:642–52. 22447155 10.1093/mp/sss025

[CR14] Gao C, Xua H, Huang J, Sun B, Zhang F, Savage Z, et al. Pathogen manipulation of chloroplast function triggers a light-dependent immune recognition. Proc Natl Acad Sci. 2020;117:9613–20. 32284406 10.1073/pnas.2002759117 PMC7196767

[CR15] Genschik P, Sumara I, Lechner E. The emerging family of CULLIN3-RING ubiquitin ligases (CRL3s): cellular functions and disease implications. EMBO J. 2013;32:2307–20. 23912815 10.1038/emboj.2013.173 PMC3770339

[CR16] Guo L, Qi Y, Mu Y, Zhou J, Lu W, Tian Z. Potato StLecRK-IV.1 negatively regulates late blight resistance by affecting the stability of a positive regulator StTET8. Hortic Res. 2022;9:uhac010. 35147183 10.1093/hr/uhac010 PMC9016858

[CR17] Haga K, Tsuchida-Mayama T, Yamada M, Sakai T. *Arabidopsis* ROOT PHOTOTROPISM2 contributes to the adaptation to high-intensity light in phototropic responses. Plant Cell. 2015;27:1098–112. 25873385 10.1105/tpc.15.00178 PMC4558708

[CR18] Han J, Wang X, Wang F, Zhao Z, Li G, Zhu X, et al. The fungal effector Avr-Pita suppresses innate immunity by increasing COX activity in rice mitochondria. Rice. 2021;14:12. 33443630 10.1186/s12284-021-00453-4 PMC7809080

[CR19] Harada A, Takemiya A, Inoue SI, Sakai T, Shimazaki KI. Role of RPT2 in leaf positioning and flattening and a possible inhibition of phot2 signaling by phot1. Plant Cell Physiol. 2013;54:36–47. 22739508 10.1093/pcp/pcs094

[CR20] Harper SM, Neil LC, Gardner KH. Structural basis of a phototropin light switch. Science. 2003;301:1541–4. 12970567 10.1126/science.1086810

[CR21] Hart JE, Gardner KH. Lighting the way: recent insights into the structure and regulation of phototropin blue light receptors. J Biol Chem. 2021;296:100594. 33781746 10.1016/j.jbc.2021.100594 PMC8086140

[CR22] He Q, Naqvi S, McLellan H, Boevink PC, Champouret N, Hein I, et al. Plant pathogen effector utilizes host susceptibility factor NRL1 to degrade the immune regulator SWAP70. Proc Natl Acad Sci U S A. 2018;115:7834–43. 10.1073/pnas.1808585115 PMC6099861 30049706

[CR23] Hou S, Rodrigues O, Liu Z, Shan L, He P. Small holes, big impact: stomata in plant-pathogen-climate epic trifecta. Mol Plant. 2024;17:26–49. 38041402 10.1016/j.molp.2023.11.011 PMC10872522

[CR24] Igamberdiev AU, Bykova NV. Mitochondria in photosynthetic cells: coordinating redox control and energy balance. Plant Physiol. 2023;191:2104–19. 36440979 10.1093/plphys/kiac541 PMC10069911

[CR25] Inada S, Ohgishi M, Mayama T, Okada K, Sakai T. RPT2 is a signal transducer involved in phototropic response and stomatal opening by association with phototropin 1 in *Arabidopsis thaliana*. Plant Cell. 2004;16:887–96. 15031408 10.1105/tpc.019901 PMC412863

[CR26] Inoue SI, Kinoshita T, Matsumoto M, Nakayama KI, Doi M, Shimazaki KI. Blue light-induced autophosphorylation of phototropin is a primary step for signaling. Proc Natl Acad Sci U S A. 2008;105:5626–31. 18378899 10.1073/pnas.0709189105 PMC2291087

[CR27] Inoue SI, Matsushita T, Tomokiyo Y, Matsumoto M, Nakayama KI, Kinoshita T, et al. Functional analyses of the activation loop of Phototropin 2 in *Arabidopsis*. Plant Physiol. 2011;156:117–28. 21427282 10.1104/pp.111.175943 PMC3091063

[CR28] Jarillo JA, Gabrys H, Capel J, Alonso JM, Ecker JR, Cashmore AR. Phototropin-related NPL1 controls chloroplast relocation induced by biue light. Nature. 2001;410:952–4. 11309623 10.1038/35073622

[CR29] Jeong RD, Chandra-Shekara AC, Barman SR, Navarre D, Klessig DF, Kachroo A, et al. Cryptochrome 2 and phototropin 2 regulate resistance protein-mediated viral defense by negatively regulating an E3 ubiquitin ligase. Proc Natl Acad Sci. 2010;107:13538–43. 20624951 10.1073/pnas.1004529107 PMC2922132

[CR30] Jeong RD, Kachroo A, Kachroo P. Blue light photoreceptors are required for the stability and function of a resistance protein mediating viral defense in *Arabidopsis*. Plant Signal Behav. 2010;5:1504–9. 21057210 10.4161/psb.5.11.13705 PMC3115268

[CR31] Kagawa T, Wadalf M. Blue light-induced chloroplast relocation in *Arabidopsis thaliana* as analyzed by microbeam irradiation. Plant Cell Physiol. 2000;41:84–93. 10750712 10.1093/pcp/41.1.84

[CR32] Kagawa T, Sakai T, Suetsugu N, Oikawa K, Ishiguro S, Kato T, et al. *Arabidopsis* NPL1: a phototropin homolog controlling the chloroplast high-light avoidance response. Science. 2001;291:2138–41. 11251116 10.1126/science.291.5511.2138

[CR33] Klupczyńska EA, Dietz KJ, Małecka A, Ratajczak E. Mitochondrial peroxiredoxin-IIF (PRXIIF) activity and function during seed aging. Antioxidants. 2022;11:1126. 35883717 10.3390/antiox11071226 PMC9311518

[CR34] Krek W. BTB proteins as henchmen of CUL3-based ubiquitin ligases. Nat Cell Biol. 2003;5:950–1. 14593416 10.1038/ncb1103-950

[CR35] Liao Y, Cui R, Xu X, Cheng Q, Li X. Jasmonic acid- and ethylene-induced mitochondrial alternative oxidase stimulates *Marssonina brunnea* defense in poplar. Plant Cell Physiol. 2020;61:2031–42. 10.1093/pcp/pcaa117 32946565

[CR36] Liebthal M, Maynard D, Dietz KJ. Peroxiredoxins and redox signaling in plants. Antioxid Redox Signal. 2018;28:609–24. 28594234 10.1089/ars.2017.7164 PMC5806080

[CR37] Lin T, Qiu H, Cheng D, Sun Q, Liu L, Tian Z. Potato NPH3/RPT2-like (NRL) member StNRL-9 interacts with Stphots and negatively regulates late blight resistance. Physiol Plant. 2024;176:e14594. 39474667 10.1111/ppl.14594

[CR38] Liscum E, Askinosie SK, Leuchtman DL, Morrow J, Willenburg KT, Coats DR. Phototropism: growing towards an understanding of plant movement. Plant Cell. 2014;26:38–55. 24481074 10.1105/tpc.113.119727 PMC3963583

[CR39] Littlejohn GR, Breen S, Smirnoff N, Grant M. Chloroplast immunity illuminated. New Phytologist. 2021;229:3088–107. 33206379 10.1111/nph.17076

[CR40] Liu J, Gong P, Lu R, Lozano-Durán R, Zhou X, Li F. Chloroplast immunity: a cornerstone of plant defense. Mol Plant. 2024;17:686–8. 38509708 10.1016/j.molp.2024.03.012

[CR41] Livak KJ, Schmittgen TD. Analysis of relative gene expression data using real-time quantitative PCR and the 2^−ΔΔCT^ method. Methods. 2001;25:402–8. 11846609 10.1006/meth.2001.1262

[CR42] McLellan H, Boevink PC, Armstrong MR, Pritchard L, Gomez S, Morales J, et al. An RxLR effector from *Phytophthora infestans* prevents re-localisation of two plant NAC transcription factors from the endoplasmic reticulum to the nucleus. PLoS Pathog. 2013;9:e1003670. 24130484 10.1371/journal.ppat.1003670 PMC3795001

[CR43] Medina-Puche L, Tan H, Dogra V, Wu M, Rosas-Diaz T, Wang L, et al. A defense pathway linking plasma membrane and chloroplasts and co-opted by pathogens. Cell. 2020;182:1109–24. 32841601 10.1016/j.cell.2020.07.020

[CR44] Naqvi S, He Q, Trusch F, Qiu H, Pham J, Sun Q, et al. Blue-light receptor phototropin 1 suppresses immunity to promote *Phytophthora infestans* infection. New Phytol. 2022;233:2282–93. 34923631 10.1111/nph.17929 PMC9255860

[CR45] Navrot N, Rouhier N, Gelhaye E, Jacquot JP. Reactive oxygen species generation and antioxidant systems in plant mitochondria. Physiol Plant. 2007;129:185–95.

[CR46] Ng S, Ivanova A, Duncan O, Law SR, Van Aken O, De Clercq I, et al. A membrane-bound NAC transcription factor, ANAC017, mediates mitochondrial retrograde signaling in *Arabidopsis*. Plant Cell. 2013;25:3450–71. 24045017 10.1105/tpc.113.113985 PMC3809543

[CR47] Nie S, Yue H, Zhou J, Xing D. Mitochondrial-derived reactive oxygen species play a vital role in the salicylic acid signaling pathway in *Arabidopsis thaliana*. PLoS One. 2015;10:e0119853. 25811367 10.1371/journal.pone.0119853 PMC4374720

[CR48] Pfeifer A, Mathes T, Lu Y, Hegemann P, Kottke T. Blue light induces global and localized conformational changes in the kinase domain of full-length phototropin. Biochemistry. 2010;49:1024–32. 20052995 10.1021/bi9016044

[CR49] Qi Y, Wu J, Yang Z, Li H, Liu L, Wang H, et al. Chloroplast elongation factors break the growth-immunity trade-off by simultaneously promoting yield and defence. Nature Plants. 2024;10:1576–91. 39300323 10.1038/s41477-024-01793-x

[CR50] Qiu H, Wang B, Huang M, Sun X, Yu L, Cheng D, et al. A novel effector RipBT contributes to *Ralstonia solanacearum* virulence on potato. Mol Plant Pathol. 2023;24:947–60. 37154802 10.1111/mpp.13342 PMC10346376

[CR51] Rademacher EH, Offringa R. Evolutionary adaptations of plant AGC kinases: from light signaling to cell polarity regulation. Front Plant Sci. 2012;3:250. 23162562 10.3389/fpls.2012.00250 PMC3499706

[CR52] Reuter L, Schmidt T, Manishankar P, Throm C, Keicher J, Bock A, et al. Light-triggered and phosphorylation- dependent 14-3-3 association with NON-PHOTOTROPIC HYPOCOTYL 3 is required for hypocotyl phototropism. Nat Commun. 2021;12:6128. 34675219 10.1038/s41467-021-26332-6 PMC8531446

[CR53] Roberts D, Pedmale UV, Morrow J, Sachdev S, Lechner E, Tang X, et al. Modulation of phototropic responsiveness in *Arabidopsis* through ubiquitination of Phototropin 1 by the CUL3-Ring E3 ubiquitin ligase CRL3^NPH3^. Plant Cell. 2011;23:3627–40. 21990941 10.1105/tpc.111.087999 PMC3229139

[CR54] Roeber VM, Bajaj I, Rohde M, Schmülling T, Cortleven A. Light acts as a stressor and influences abiotic and biotic stress responses in plants. Plant Cell Environment. 2021;44:645–64. 33190307 10.1111/pce.13948

[CR55] Rosas-Diaz T, Zhang D, Fan P, Wang L, Ding X, Jiang Y, et al. A virus-targeted plant receptor-like kinase promotes cell-to-cell spread of RNAi. Proc Natl Acad Sci. 2018;115:1388–93. 29363594 10.1073/pnas.1715556115 PMC5819414

[CR56] Sakai T, Wada T, Ishiguro S, Okada K. RPT2: A signal transducer of the phototropic response in *Arabidopsis*. Plant Cell. 2000;12:225–36. 10662859 10.1105/tpc.12.2.225 PMC139760

[CR57] Sakai T, Kagawa T, Kasahara M, Swartz TE, Christie JM, Briggs WR, et al. *Arabidopsis* nph1 and npl1: blue light receptors that mediate both phototropism and chloroplast relocation. Proc Natl Acad Sci. 2001;98:6969–74. 11371609 10.1073/pnas.101137598 PMC34462

[CR58] Suetsugu N, Takemiya A, Kong SG, Higa T, Komatsu A, Shimazaki KI, et al. RPT2/NCH1 subfamily of NPH3-like proteins is essential for the chloroplast accumulation response in land plants. Proc Natl Acad Sci USA. 2016;113:10424–9. 27578868 10.1073/pnas.1602151113 PMC5027436

[CR59] Sullivan S, Waksman T, Paliogianni D, Henderson L, Lütkemeyer M, Suetsugu N, et al. Regulation of plant phototropic growth by NPH3/RPT2-like substrate phosphorylation and 14-3-3 binding. Nature Communications. 2021;12:6129. 34675214 10.1038/s41467-021-26333-5 PMC8531357

[CR60] Tseng TS, Briggs WR. The *Arabidopsis* rcn1-1 mutation impairs dephosphorylation of Phot2, resulting in enhanced blue light responses. Plant Cell. 2010;22:392–402. 20139163 10.1105/tpc.109.066423 PMC2845423

[CR61] Upadhyay-Tiwari N, Huang XJ, Lee YC, Singh SK, Hsu CC, Huang SS, et al. The nonphototrophic hypocotyl 3 (NPH3) domain protein NRL5 is a trafficking-associated GTPase essential for drought resistance. Sci Adv. 2024;10:eado5429. 39121213 10.1126/sciadv.ado5429 PMC11313873

[CR62] Verslues PE, Upadhyay-Tiwari N. Nonphototrophic hypocotyl 3 domain proteins: traffic directors, hitchhikers, or both? New Phytol. 2024;244:1723–31. 39425258 10.1111/nph.20211

[CR63] Waksman T, Suetsugu N, Hermanowicz P, Ronald J, Sullivan S, Łabuz J, et al. Phototropin phosphorylation of ROOT PHOTOTROPISM 2 and its role in mediating phototropism, leaf positioning, and chloroplast accumulation movement in *Arabidopsis*. Plant J. 2023;114:390–402. 36794876 10.1111/tpj.16144 PMC10953443

[CR64] Wang J, Xu G, Ning Y, Wang X, Wang GL. Mitochondrial functions in plant immunity. Trends Plant Sci. 2022;27:1063–76. 35659746 10.1016/j.tplants.2022.04.007

[CR65] Willems AR, Schwab M, Tyers M. A hitchhiker’s guide to the cullin ubiquitin ligases: SCF and its kin. Biochim Biophys Acta. 2004;1695:133–70. 15571813 10.1016/j.bbamcr.2004.09.027

[CR66] Wu L, Yang HQ. Cryptochrome 1 is implicated in promoting R protein-mediated plant resistance to *Pseudomonas syringae* in *Arabidopsis*. Mol Plant. 2010;3:539–48. 20053798 10.1093/mp/ssp107

[CR67] Xu G, Zhong X, Shi Y, Liu Z, Jiang N, Liu J, et al. A fungal effector targets a heat shock-dynamin protein complex to modulate mitochondrial dynamics and reduce plant immunity. Sci Adv. 2020;6:eabb7719. 33239288 10.1126/sciadv.abb7719 PMC7688324

[CR68] Yang L, McLellan H, Naqvi S, He Q, Boevink PC, Armstrong M, et al. Potato NPH3/RPT2-like protein StNRL1, targeted by a *Phytophthora infestans* RXLR effector, is a susceptibility factor. Plant Physiol. 2016;171:645–57. 26966171 10.1104/pp.16.00178 PMC4854710

[CR69] Yang Y, Li Y, Guang Y, Lin J, Zhou Y, Yu T, et al. Red light induces salicylic acid accumulation by activating CaHY5 to enhance pepper resistance against *Phytophthora capsici*. Hortic Res. 2023;10:uhad213. 38046851 10.1093/hr/uhad213 PMC10689078

[CR70] Yuan DP, Yang S, Feng F, Chu J, Dong H, Sun J, et al. Red-light receptor phytochrome B inhibits BZR1-NAC028-CAD8B signaling to negatively regulate rice resistance to sheath blight. Plant Cell Environ. 2023;46:1249–63. 36457051 10.1111/pce.14502

[CR71] Zhang L, Du L, Shen C, Yang Y, Poovaiah BW. Regulation of plant immunity through ubiquitin-mediated modulation of Ca^2+^-calmodulin- AtSR1/CAMTA3 signaling. Plant J. 2014;78:269–81. 24528504 10.1111/tpj.12473

[CR72] Zhang X, Wang D, Zhao P, Sun Y, Fang RX, Ye J. Near-infrared light and PIF4 promote plant antiviral defense by enhancing RNA interference. Plant Commun. 2024;5:100644. 37393430 10.1016/j.xplc.2023.100644 PMC10811336

[CR73] Zhu F, Deng XG, Xu F, Jian W, Peng XJ, Zhu T, et al. Mitochondrial alternative oxidase is involved in both compatible and incompatible host-virus combinations in *Nicotiana benthamiana*. Plant Sci. 2015;239:26–35. 26398788 10.1016/j.plantsci.2015.07.009

