# Supplementary material for: Blue light receptor phot2 collaborates with NRL-30 to negatively regulate immunity by reducing mitochondrial protein PRXIIF stability in potato
Source: Mol Hortic. 2026 Jun 2;6:44. doi: 10.1186/s43897-025-00224-5 (PMC13227800; doi:10.1186/s43897-025-00224-5)
Supplement: Supplementary file 1 — Supplementary Material 1: Fig. S1. Silencing of either Nbphot1 or Nbphot2 resulted in a significant reduction in Phytophthora infestans colonization. Fig. S2. Gene expression levels and plant phenotype of representative Stphot2-overexpression (OE) and -RNAi (Ri) transgenic potato lines. Fig. S3. Stphot1 negatively regulates potato late blight resistance. Fig. S4. Blue light negatively regulates potato late blight resistance. Fig. S5. Immunoblots showing that GFP-Stphot2 and GFP-Stphot2D764N are stably expressed in Nicotiana benthamiana leaves. Fig. S6. Split luciferase complementation assay (LCA) identified NRLs interacting with Stphot2. Fig. S7. Subcellular localization of StNRL-30. Fig. S8. Alignment of NRL-30 sequences. Fig. S9. StNRL-30 expression levels and plant growth phenotypes of virus-induced NbNRL-30-silenced N. benthamiana plants and StNRL-30-OE and -Ri potato lines. Fig. S10. St14-3-3 interacts with both StNRL-30 and Stphot2. Fig. S11. Predicted phosphorylation and 14-3-3 binding sites on StNRL-30. Fig. S12. StNRL-30 interacts with StPRXIIF. Fig. S13. StPRXIIF is located in the cytoplasm. Fig. S14. Silencing levels and plant phenotypes of virus-induced PRXIIF-silenced N. benthamiana plants. Fig. S15. Degradation of StPRXIIF mediated by StNRL-30 and Stphot2. Fig. S16. Blue light affects stability of StPRXIIF and localization of the StNRL-30–StPRXIIF complex. Fig. S17. cTP-StPRXIIF-GFP is stably expressed in N. benthamiana. [file 43897_2025_224_MOESM1_ESM.docx]

**Figure S1-17**

**
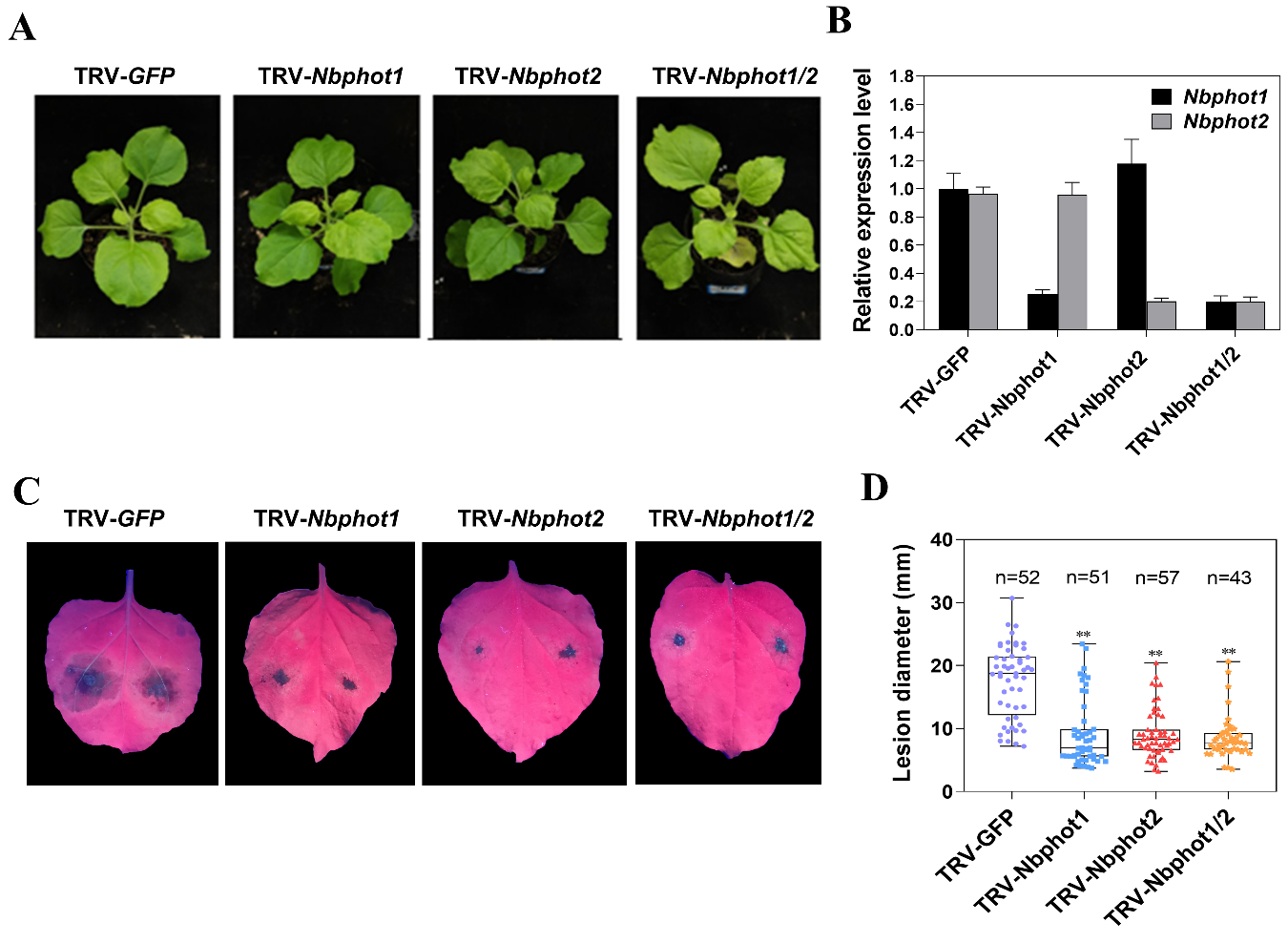
**

**Fig. S1 Silencing of either *Nbphot1* or *Nbphot2* resulted in a significant reduction in** *Phytophthora* ***infestans*** **colonization. A** Representative images of *N. benthamiana* plants expressing TRV constructs as indicated for silencing *Nbphot1*, *Nbphot2* and both *Nbphot1/2*. **B** Bar graph showing expression level of *Nbphot1* and *Nbphot2* in the VIGS plants by qRT-PCR. TRV-*GFP* plants were used as a control. The constitutive expressed *NbEF1α* was used as internal reference gene, according to the 2^−ΔΔCt^ method. Data represent means ± SEM from three independent biological repeats. **C** Representative images showing disease lesion diameters on leaves from VIGS plants at 6 days post inoculation (dpi) with *P. infestans* isolate 88069. **D** Boxplots showing lesion diameter (mm) on inoculated leaves. Dots represent individual datapoints, and horizontal lines indicate the median. Statistical analysis was performed using One-way analysis of variance (ANOVA) (**, *p* < 0.01; three independent repeats with precise n numbers indicated).


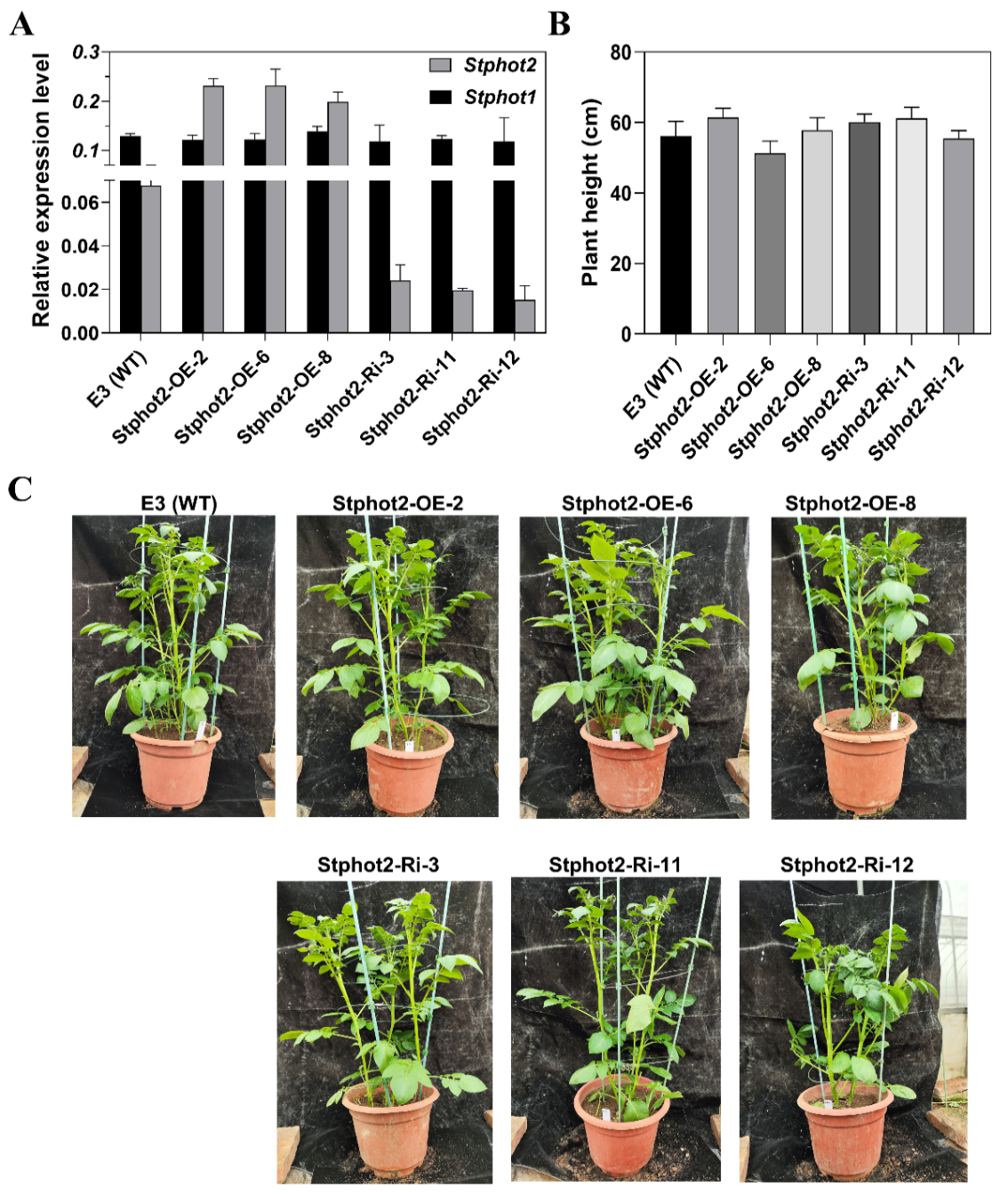


**Fig. S2 Gene expression levels and plant phenotype of representative** *Stphot2*-overexpression (OE) and -RNAi (Ri) **transgenic potato lines.** **A** Bar graph showing expression level of *Stphot1* and *Stphot2* in *Stphot2* overexpression (OE)- and RNA interference (Ri)- lines tested by qRT-PCR, with wild-type (WT) ‘E3’ serving as a control. The constitutive expressed *StEF1α* was used as internal reference gene. The expression level was calculated by the comparative Ct method. Data represent means ± SEM of three independent biological repeats. **B** Bar graph shows that plant height of *Stphot2*-OE and Ri- lines has no obvious difference compared with WT ‘E3’ control. **C** Representative plant images of *Stphot2*-OE and Ri- lines illustrating there is no adverse growth effects compared with ‘E3’ control.

**
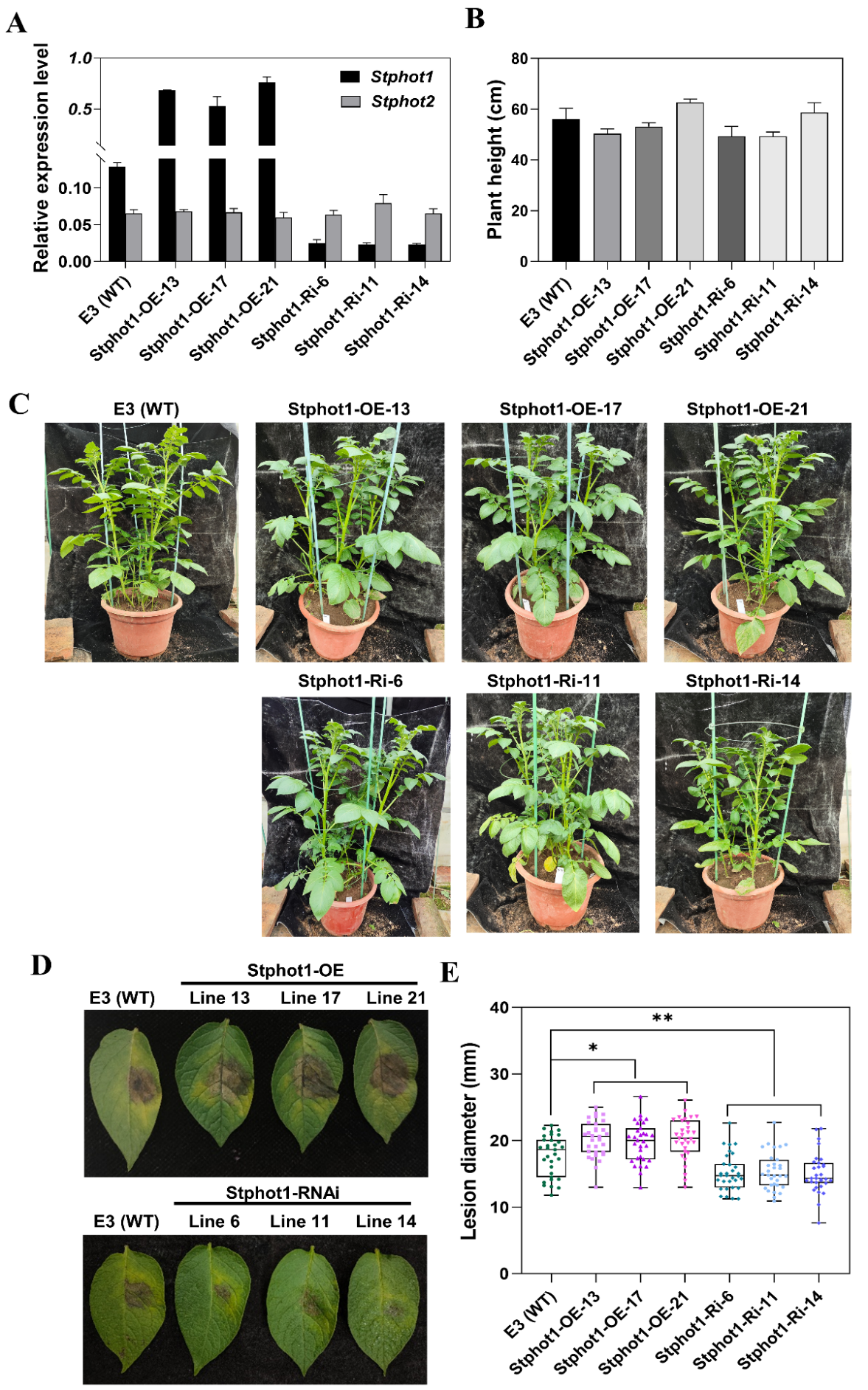
**

**Fig. S3 *Stphot1* negatively regulates potato late blight resistance.** **A** Bar graph showing *Stphot1* and *Stphot2* expression level in *Stphot1*-OE and Ri- lines tested by qRT-PCR. Wild-type (WT) ‘E3’ was used as a control. *StEF1α* was used as internal reference gene. The expression level was calculated by the comparative Ct method. Data represent means ± SEM of three independent biological repeats. **B** Bar graph shows that there was no obvious difference on plant height of *Stphot1*-OE and Ri- lines compared with the ‘E3’ control. **C** Representative plant images of *Stphot1*-OE and Ri lines. There were no adverse growth effects compared with the ‘E3’ control. **D** and **E** Representative leaf images showing disease lesion diameters on potato *Stphot1*-OE and Ri- lines at 5 dpi with *P. infestans* isolate HB09-14-2, with ‘E3’ serving as the control. Boxplots showing disease lesion diameter (mm) on inoculated leaves. Dots represent individual datapoints and horizontal lines indicate the median. One-way ANOVA was used for statistical analysis (*, *p* < 0.05; **, *p* < 0.01; three independent repeats; n = 31).


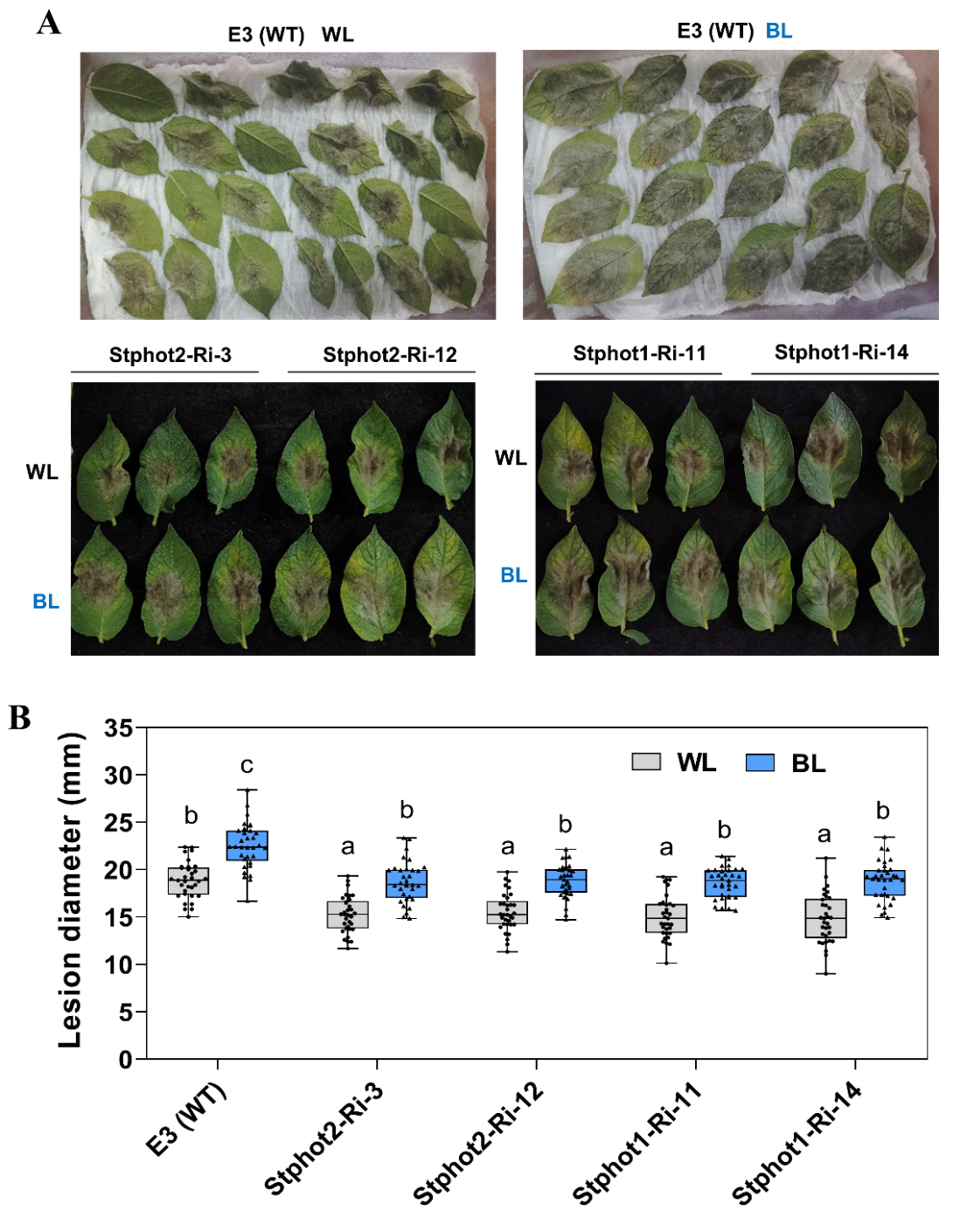


**Fig. S4 Blue light negatively regulates potato late blight resistance.** **A** Representative leaf images demonstrating disease lesions in wild-type ‘E3’ potato leaves. After inoculation with *P. infestans* on detached leaves from wild-type ‘E3’ or Ri lines, the inoculated leaves were respectively placed in incubators with 16 h BL / 8 h dark light cycle (BL) or 16 h WL / 8 h dark light cycle (WL). Pictures were taken 5 days after *P. infestans* isolate HB09-14-2 inoculation. **B** Boxplots showing disease lesion diameter (mm) on inoculated leaves. Dots represent individual datapoints and horizontal lines indicate the median. One-way ANOVA with Tukey’s HSD post-hoc test was used for statistical analysis (different letters indicate significant differences, *p* < 0.01; three independent repeats; n > 30).

**
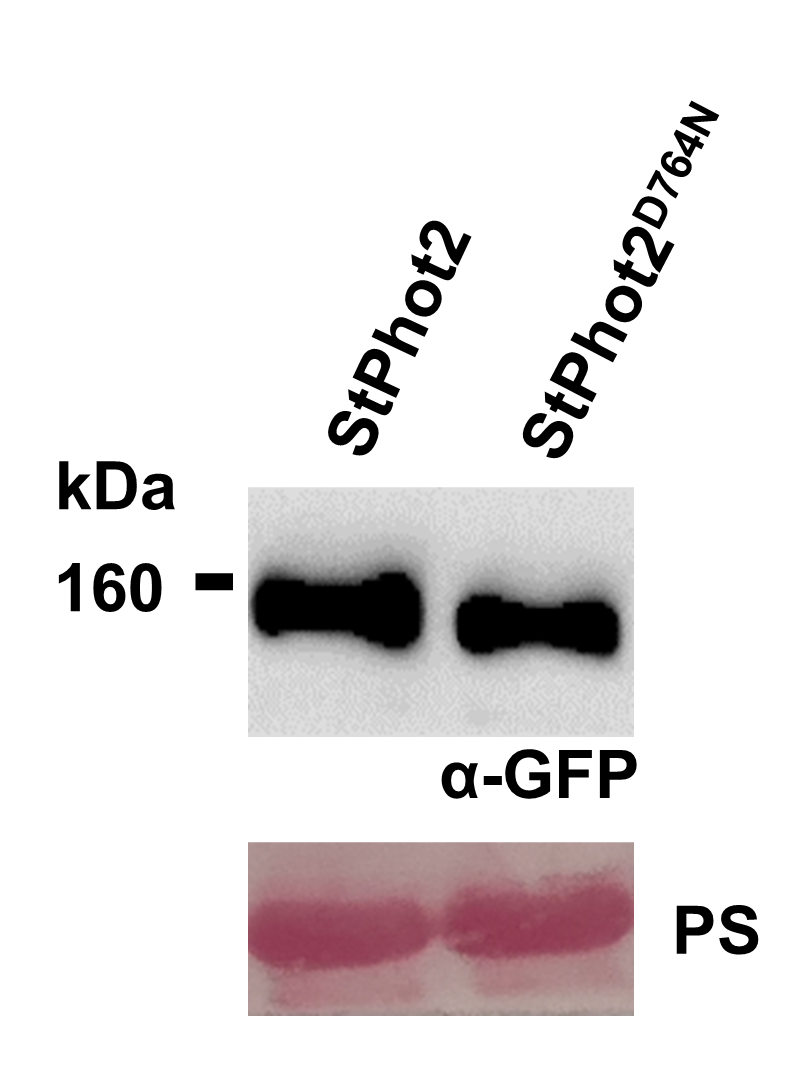
**

**Fig. S5** Immunoblots showing **that GFP-Stphot2 and GFP-Stphot2^D764N^ are stably expressed in** *Nicotiana* ***benthamiana* leaves.** Constructs were expressed in *N. benthamiana* leaves by agroinfiltration. Forty-eight hours later, agroinfiltrated leaves were collected for protein extraction and west blotting.

**
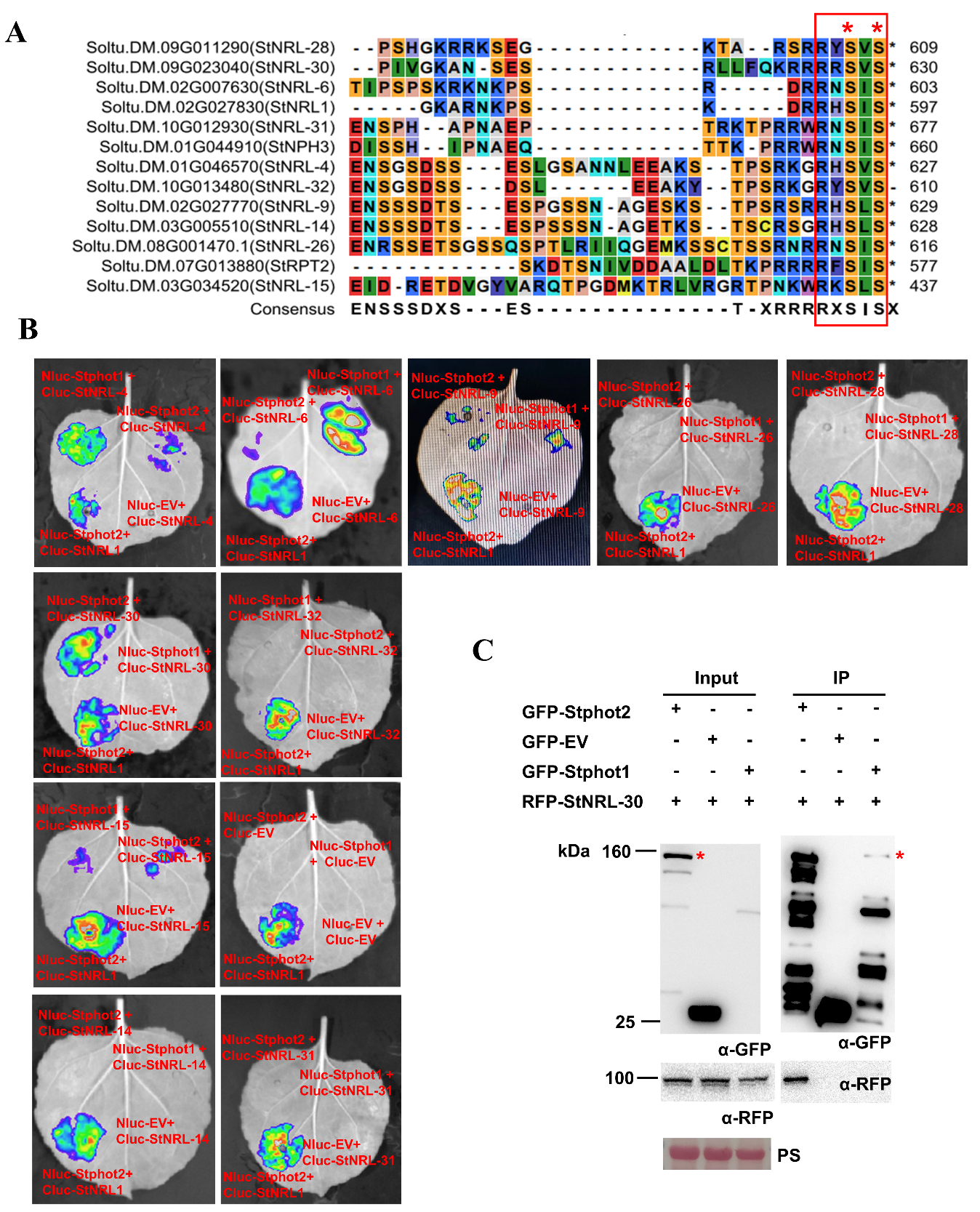
**

**Fig. S6 Split luciferase complementation assay** (**LCA)** identified **NRLs interacting with Stphot2. A** NRL family members contain RxSΦS motif. Image was generated by CLC Sequence Viewer 6. Conserved amino acids are indicated with the same color. * indicates two conserved Ser residues. **B** LCAs showing the interactions of different construct combinations. The luminescence signal was observed and imaged at 48 h post agroinfiltration (hpa). StNRL1 was used as a positive control for interaction with Stphot2. Strong luminescence signal was detected in the combination of Stphot2-nLUC + cLUC-StNRL-30. **C** Independent immunoblot replicate demonstrates that Stphot2, but not Stphot1, interacts with StNRL-30. EV-GFP was used as negative control. Constructs expression in *N. benthamiana* leaves are indicated by a plus sign (+). Protein size markers are given in kilodalton (kDa), and protein loading is shown by Ponceau stain (PS). * indicates target protein bands.


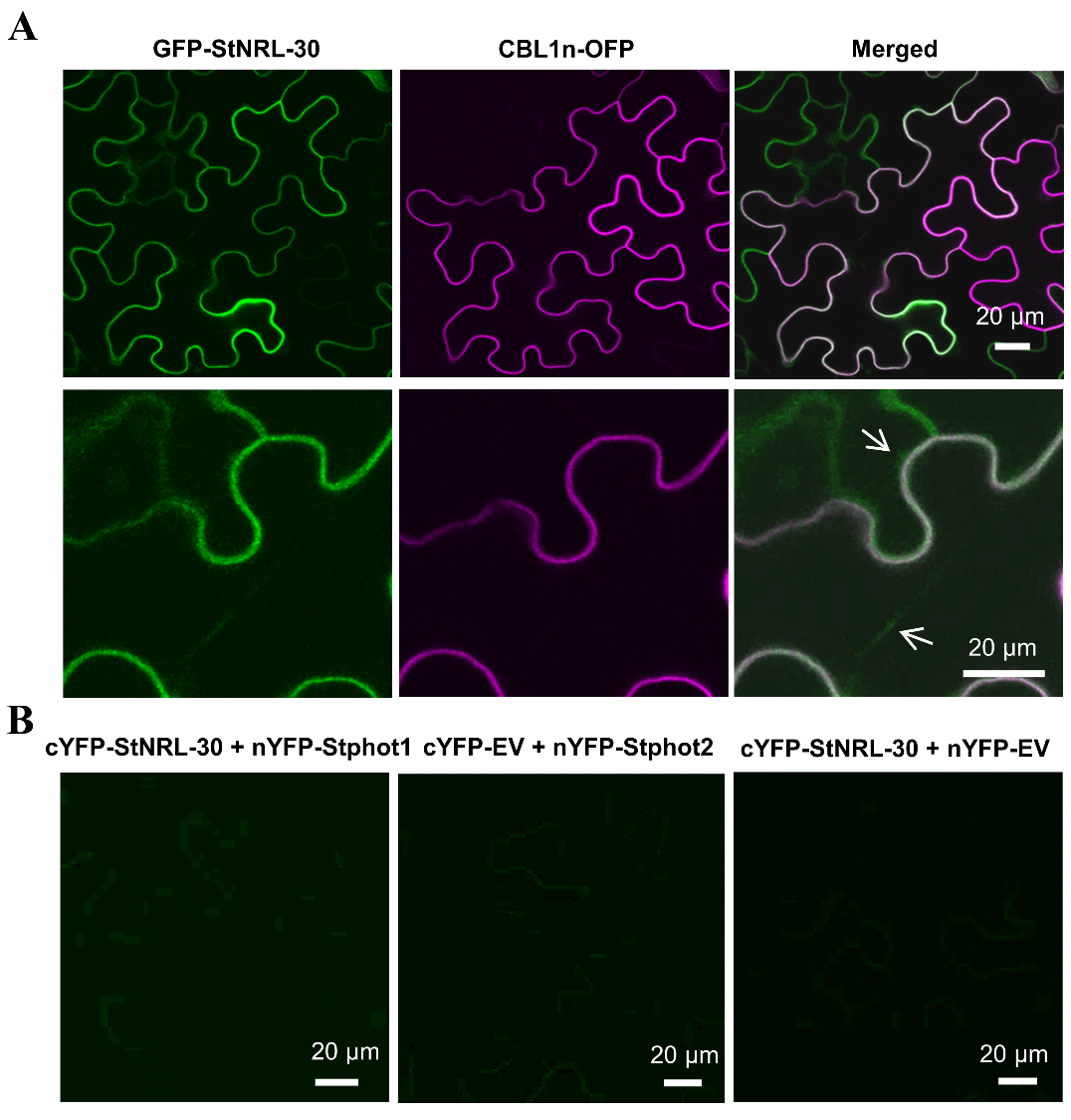


**Fig. S7 Subcellular localization of StNRL-30. A** Confocal images show that StNRL-30 was dominantly located on the PM with weak cytoplasmic background. CBL1n-OFP is a membrane maker. Arrows showing cytoplasmic signal of GFP-StNRL-30. **B** Microscopy images show that no yellow florescence signal was observed in constructs combination of cYFP-StNRL-30 + nYFP-Stphot1, cYFP-EV + nYFP-Stphot2 and cYFP-StNRL-30 + nYFP-EV. Construct combinations were expressed in *N. benthamiana* leaves by agroinfiltration.

**
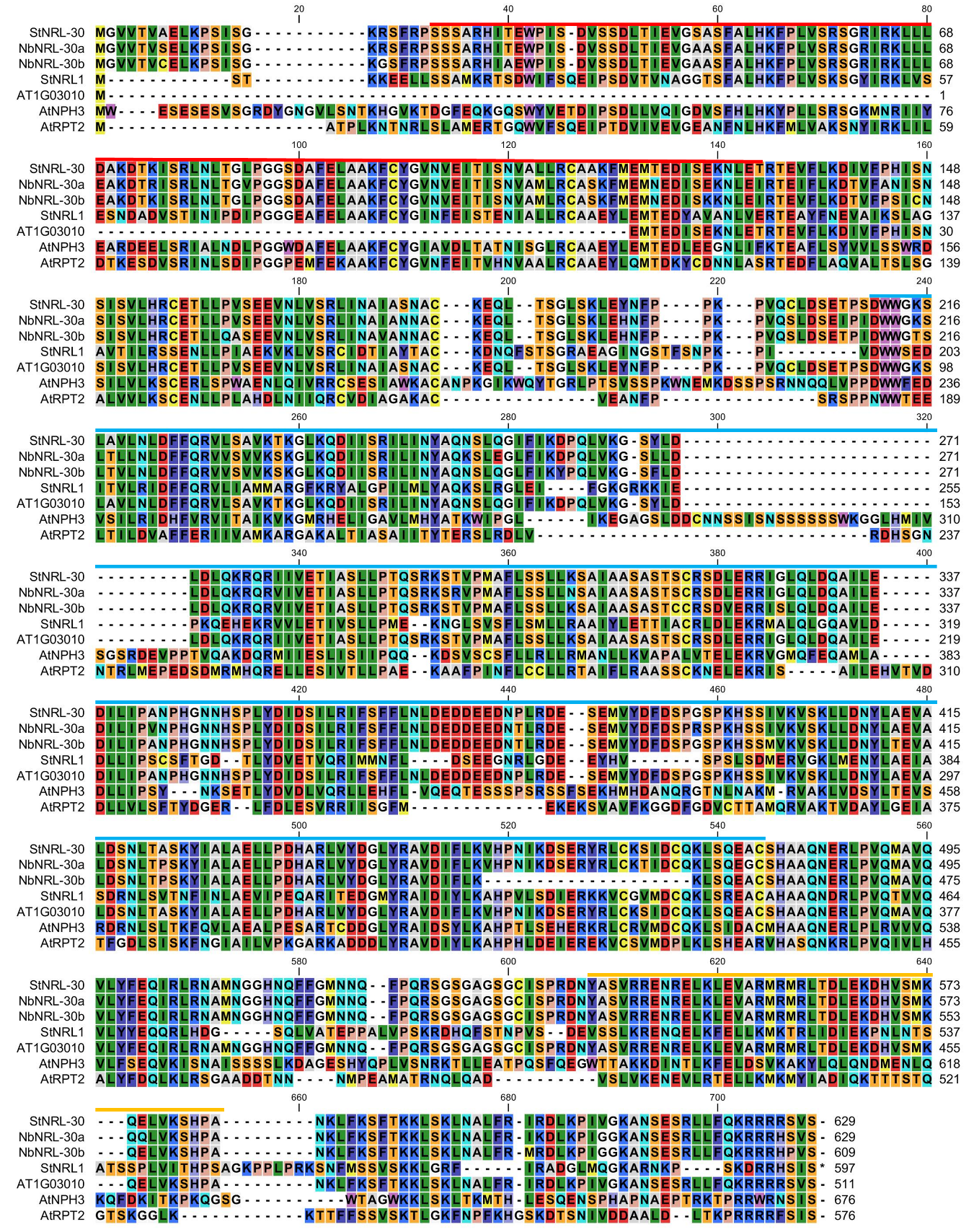
**

**Fig. S8 Alignment of NRL-30 sequences.** Amino acid alignment of NRL proteins from *Arabidopsis thaliana* (At), *N. benthamiana* (Nb) and *S. tuberosum* (St), was conducted by CLC Sequence Viewer 6. Conserved amino acids are indicated with the same color. The red line shows the BTB/POZ domain. The blue line shows the NPH3 family domain. The orange line shows the coiled-coiled domain.

**
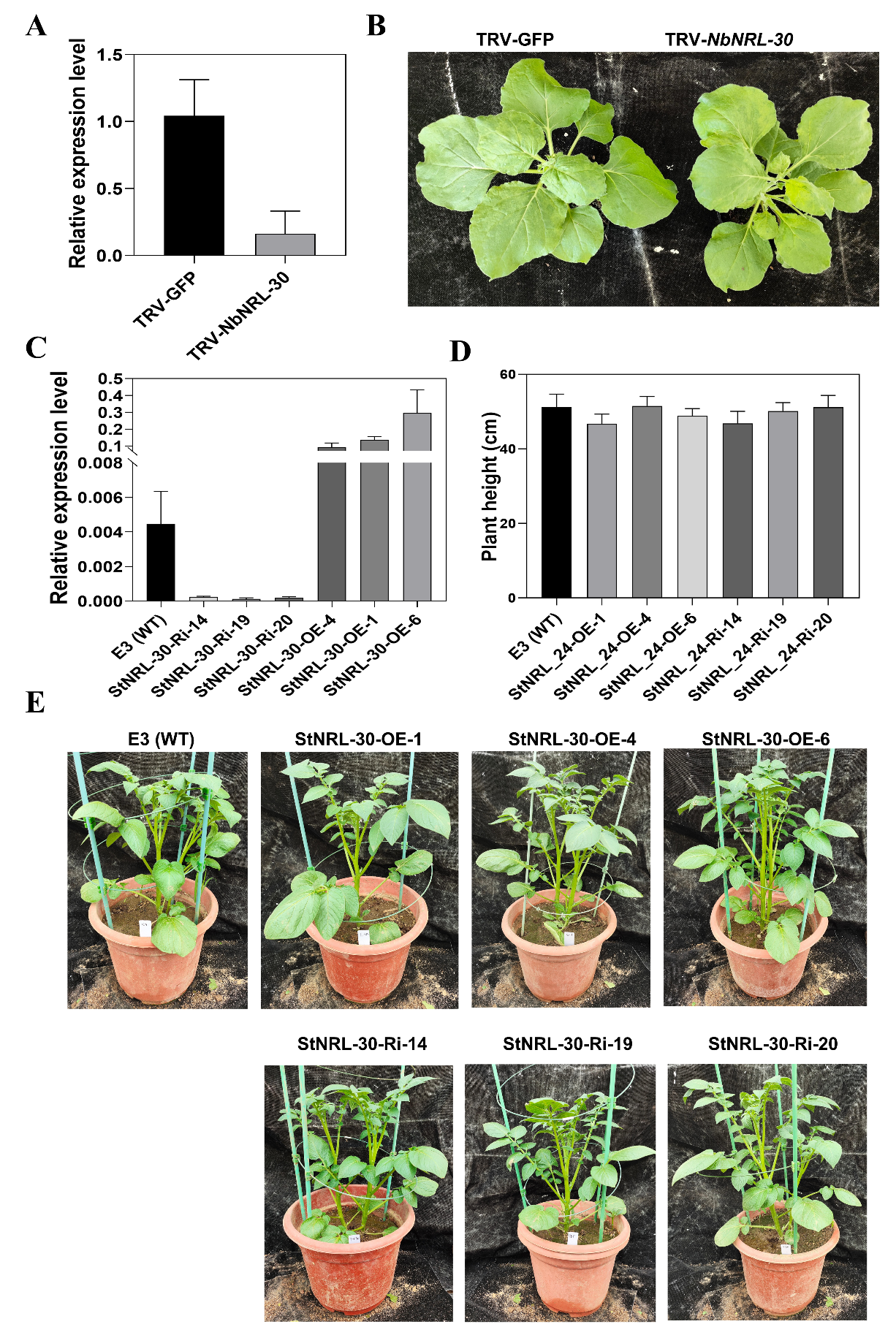
**

**Fig. S9** ***StNRL-30* expression levels and plant growth phenotypes of** virus-induced *NbNRL-30*-silenced ***N. benthamiana* plants, *StNRL-30*** **-OE and -Ri potato lines. A** Bar graph showing *NbNRL-30* expression level in its VIGS plants tested by qRT-PCR. TRV-*GFP* *N. benthamiana* plants were used as control. The constitutive expressed *NbEF1α* was used as internal reference gene. The expression level was calculated by the 2^−ΔΔCt^ method. Data represent means ± SEM from three independent biological repeats. **B** *NRL-30* VIGS plant shows slightly stunted growth compared to TRV-*GFP* plants. **C** Bar graph showing StNRL-30 expression levels in potato *StNRL-30* overexpression (OE)- and RNAi (Ri)- lines tested by qRT-PCR. Wild-type (WT) potato ‘E3’ was used as a control. *StEF1α* was used as internal reference gene. The expression level was calculated by the comparative Ct method. Data represent means ± SEM from three independent biological repeats. **D** Bar graph shows that plant height of *StNRL-30* OE- and Ri- lines has no obvious difference compared with WT ‘E3’ control. **E** Representative images of *StNRL-30* OE- and Ri- lines showing no adverse growth effects compared with WT ‘E3’ control.


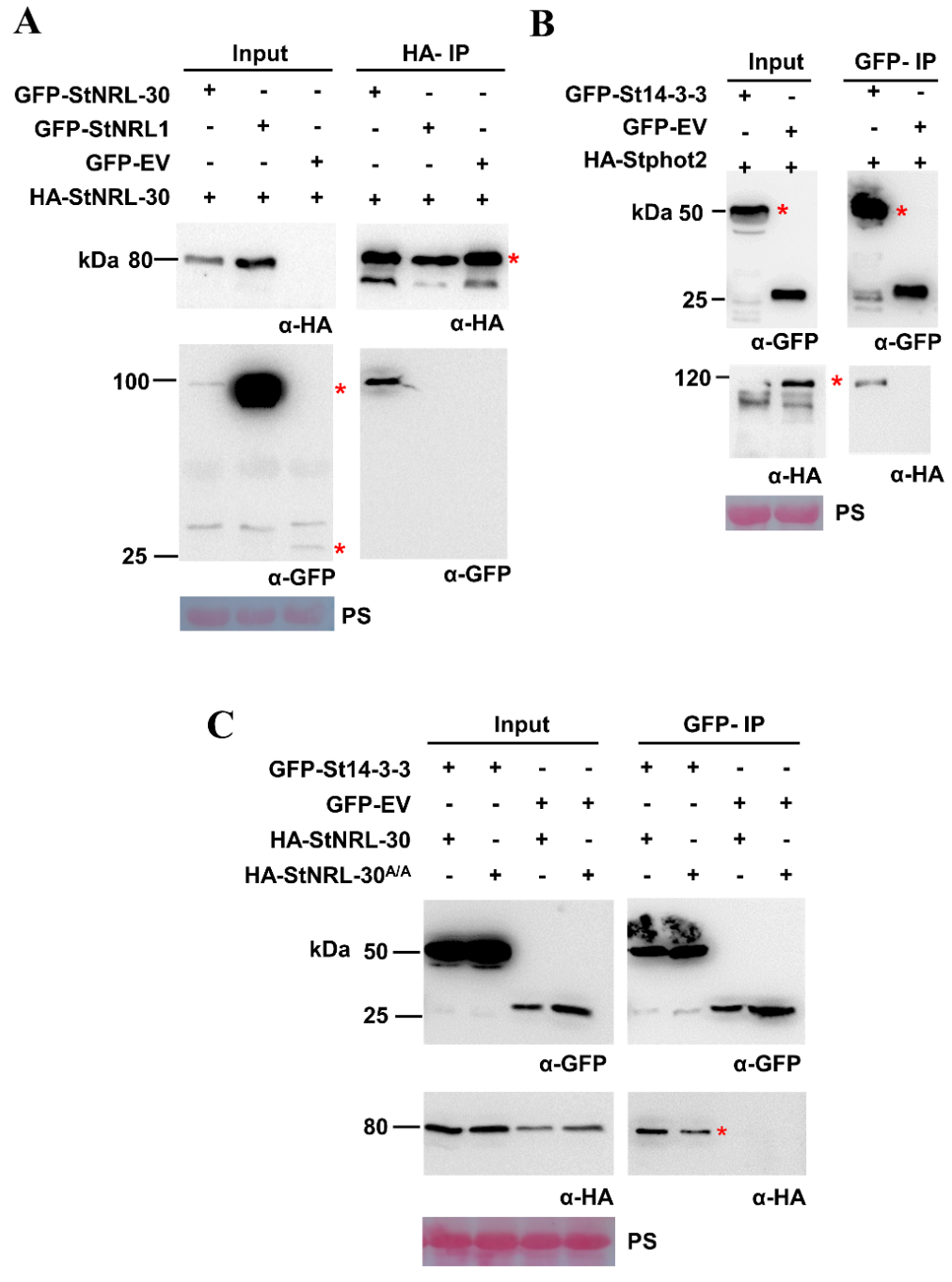


**Fig. S10 St14-3-3 interacts with both StNRL-30 and Stphot2. A** Co-IP assay shows that HA-StNRL-30 interacts with GFP-StNRL-30, but not with GFP-StNRL1. HA-agarose beads were used for immunoprecipitating leaf extract proteins. **B** Co-IP assay shows that GFP-St14-3-3 interacts with HA-Stphot2. GFP-agarose beads were used for immunoprecipitating leaf extract proteins. **C** Co-IP assay shows that GFP-St14-3-3 interacts with HA-StNRL-30. GFP-agarose beads were used for immunoprecipitating leaf extract proteins. Construct combinations were expressed in *N. benthamiana* leaves by agroinfiltration. Constructs expression in *N. benthamian*a leaves are indicated by a plus sign (+). Protein size markers are given in kilodalton (kDa), and protein loading is shown by Ponceau stain (PS). * indicates target protein bands.


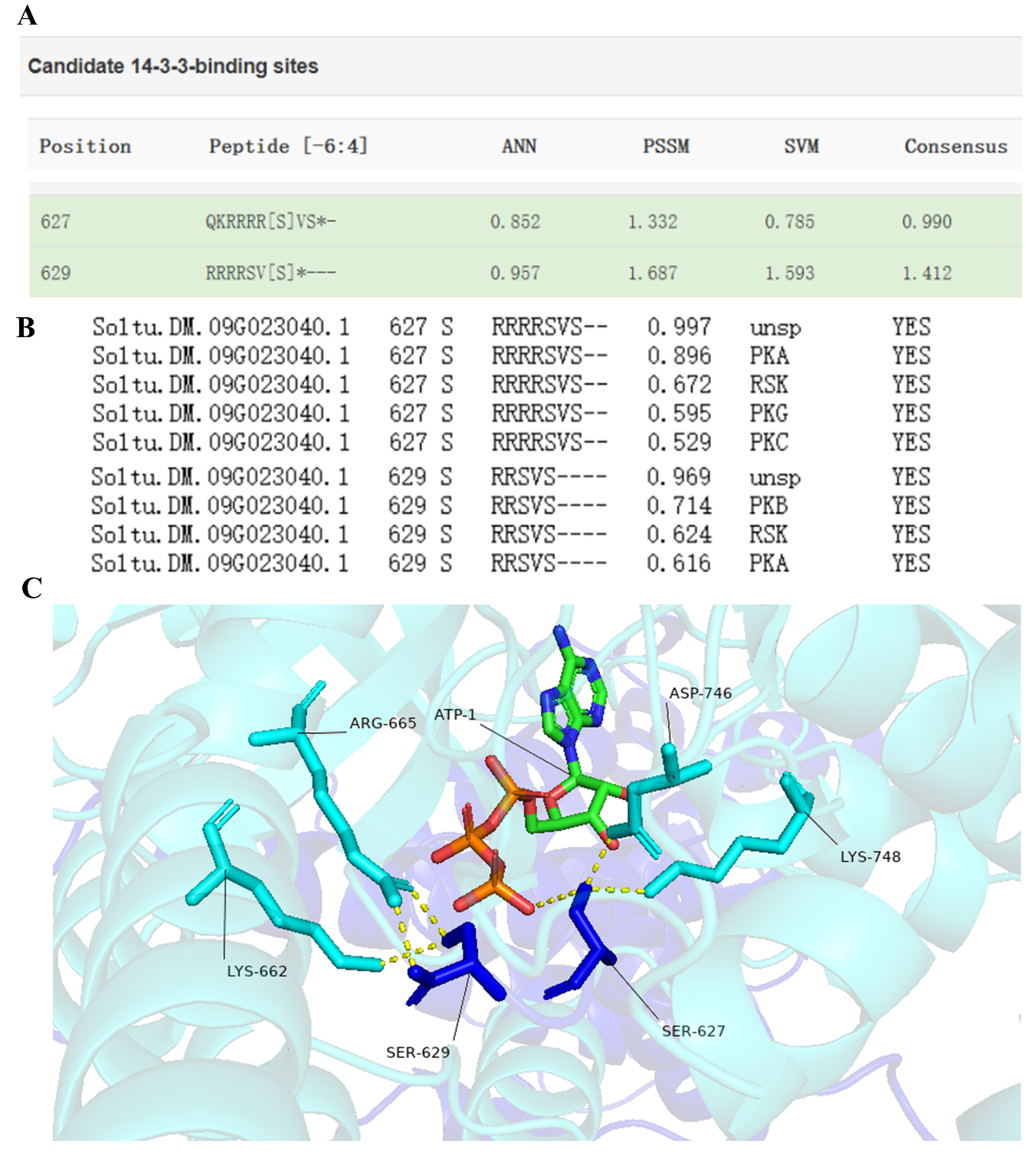


**Fig. S11** **Predicted** **phosphorylation and** **14-3-3 binding sites on** **StNRL-30. A** Phosphorylation sites of StNRL-30 predicted in the website ([https://www.cbs.dtu.dk/ services/NetPhos-3.1/](https://www.cbs.dtu.dk/%20services/NetPhos-3.1/)). **B** 14-3-3 binding sites predicted in the website (<http://www.compbio.dundee.ac.uk/1433pred>). **C** Interaction sites of StNRL-30 with 14-3-3 predicted by the Alphafold3 (<https://alphafoldserver.com/>). SER-627 and SER-629 represent two Ser residues in the RxSΦS motif of StNRL-30. Ser-627 is a potential phosphorylation site indicated by that it is an ATP binding site.

**
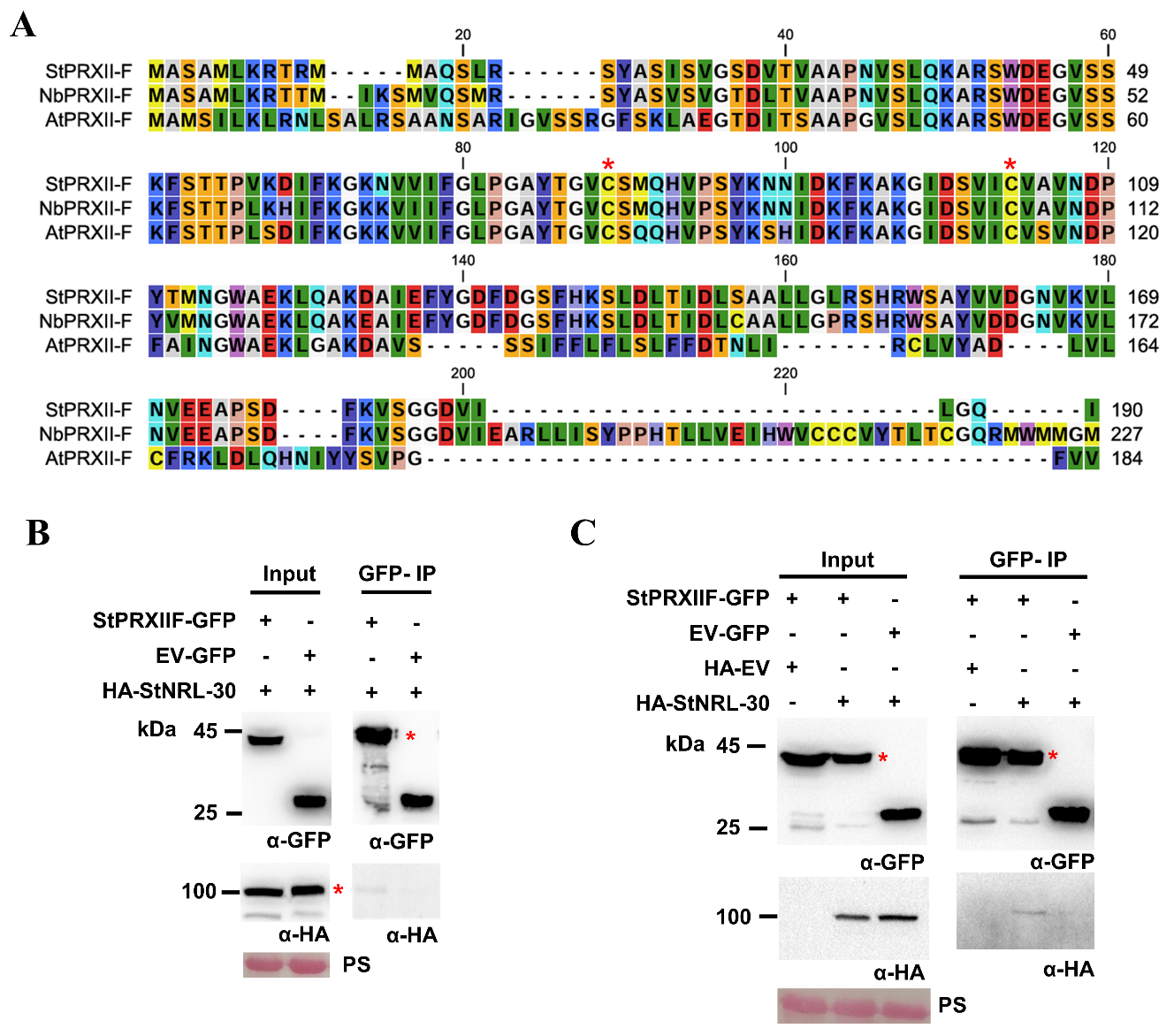
**

**Fig. S12 StNRL-30 interacts with StPRXIIF. A** Alignment of PRXIIF proteins from *A. thaliana* (At), *N. benthamiana* (Nb) and *S. tuberosum* (St) using ClastalX and showing by CLC Sequence Viewer 6. Conserved amino acids are indicated with the same color. **B** and **C** Independent immunoblot replicates demonstrate that StPRXIIF interacts with StNRL-30. EV-GFP and HA-EV were used as negative controls. GFP-agarose beads were used for immunoprecipitating leaf extractd proteins. Construct combinations were expressed in *N. benthamiana* leaves by agroinfiltration. Constructs expressed in *N. benthamiana* leaves are represented by a plus sign (+). Protein size markers are given in kilodalton (kDa), and protein loading is shown by Ponceau stain (PS). * indicates target protein bands.

**
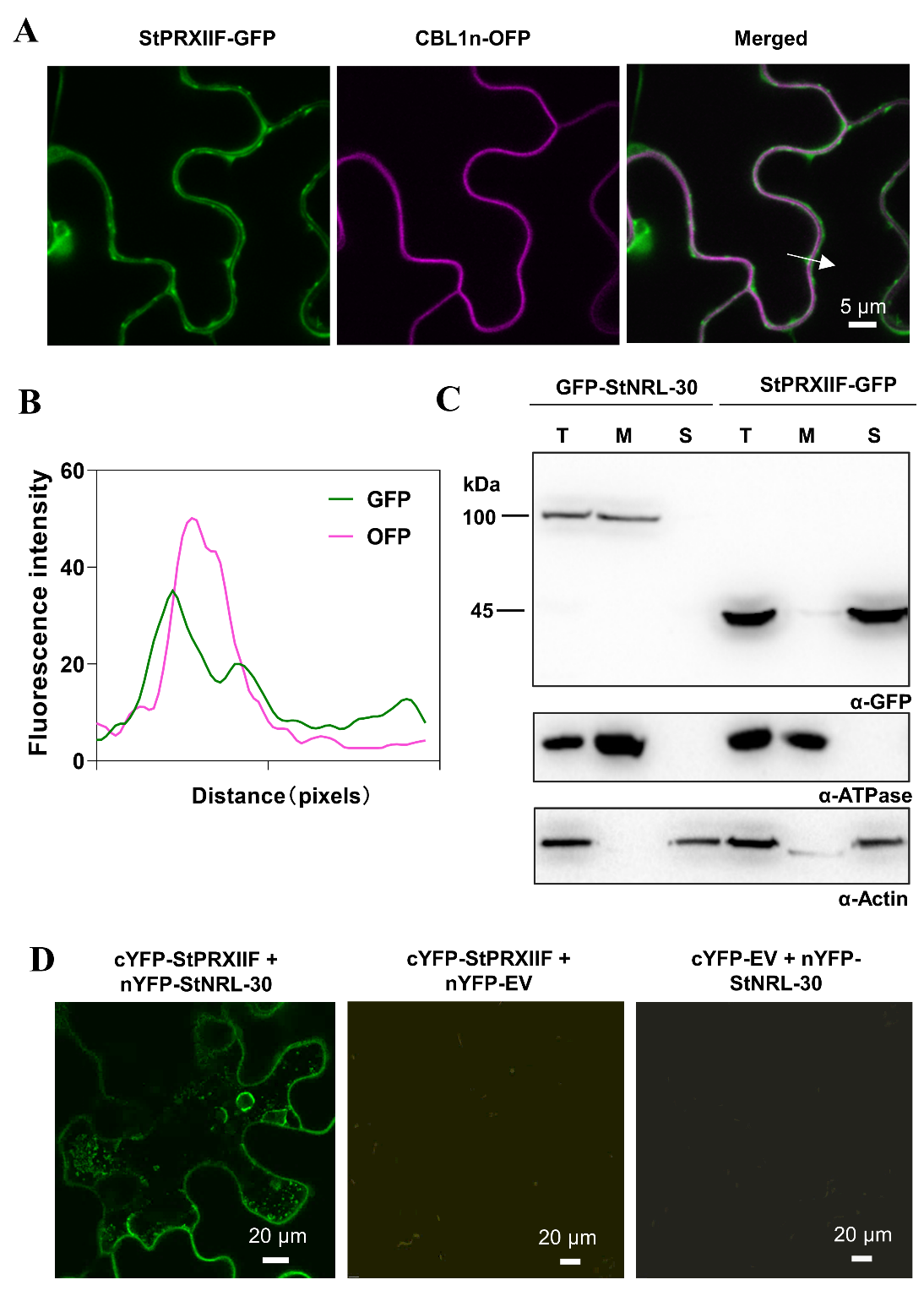
**

**Fig. S13** **StPRXIIF is located in the cytoplasm. A** Co-expression of StPRXIIF-GFP and CBL1n-OFP in *N. benthamiana* cells. Photographs were taken at 48 hpa. **B** Plots showing intensity of green fluorescence and OFP fluorescence across arrows indicates path in **A**. **C** Detection of StPRXIIF-GFP in the total proteins (T), the plasma membrane fraction (M) and the cytoplasmic solute fraction (S) by western blot. StPRXIIF-GFP and GFP-StNRL-30 were transiently expressed in *N. benthamiana* leaves. The anti-ATPase was used to indicate the membrane fraction. The anti-Actin was used to indicate the cytoplasmic solute. **D** Representative images showing proteins localization of cYFP-StPRXIIF + nYFP-StNRL-30, cYFP-StPRXIIF + nYFP-EV or cYFP-EV + nYFP-StNRL-30. Yellow fluorescence was shown in green fluorescence. Construct combinations were expressed in *N. benthamiana* leaves by agroinfiltration.


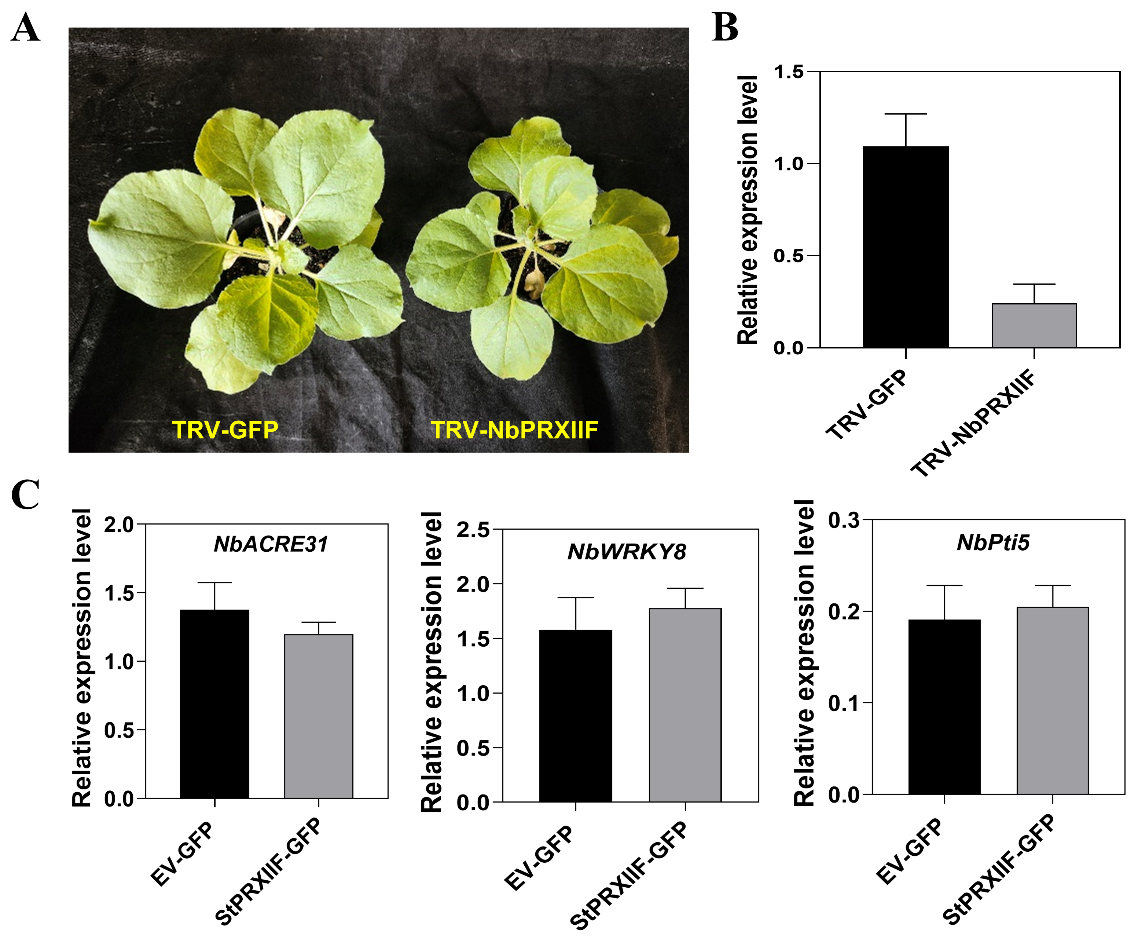


**Fig. S14 Silencing levels and plant phenotypes of** virus-induced *PRXIIF*-silenced ***N. benthamiana* plants. A** Representative image shows that TRV-*PRXIIF* plants display slightly stunted growth compared to TRV-*GFP* plants. **B** Bar graph showing *NbPRXIIF* expression level in *NbPRXIIF* VIGS plants tested by qRT-PCR. TRV-*GFP* plants were used as control. *NbEF1**α* was used as internal reference gene. The expression level was calculated by the 2^−ΔΔCt^ method. Data represent means ± SEM from three independent biological repeats. **C** Expression levels of PTI marker genes in *StPRXIIF* transient expressed *N. benthamiana* leaves tested by RT-qPCR. Leaf samples were collected at 30 min after flg22 treatment. *NbEF1α* was used as internal control. The expression level was calculated by the comparative Ct method. Results are presented as the mean ± SEM of four repeats (three leaves from different plants were collected together as one repeat).


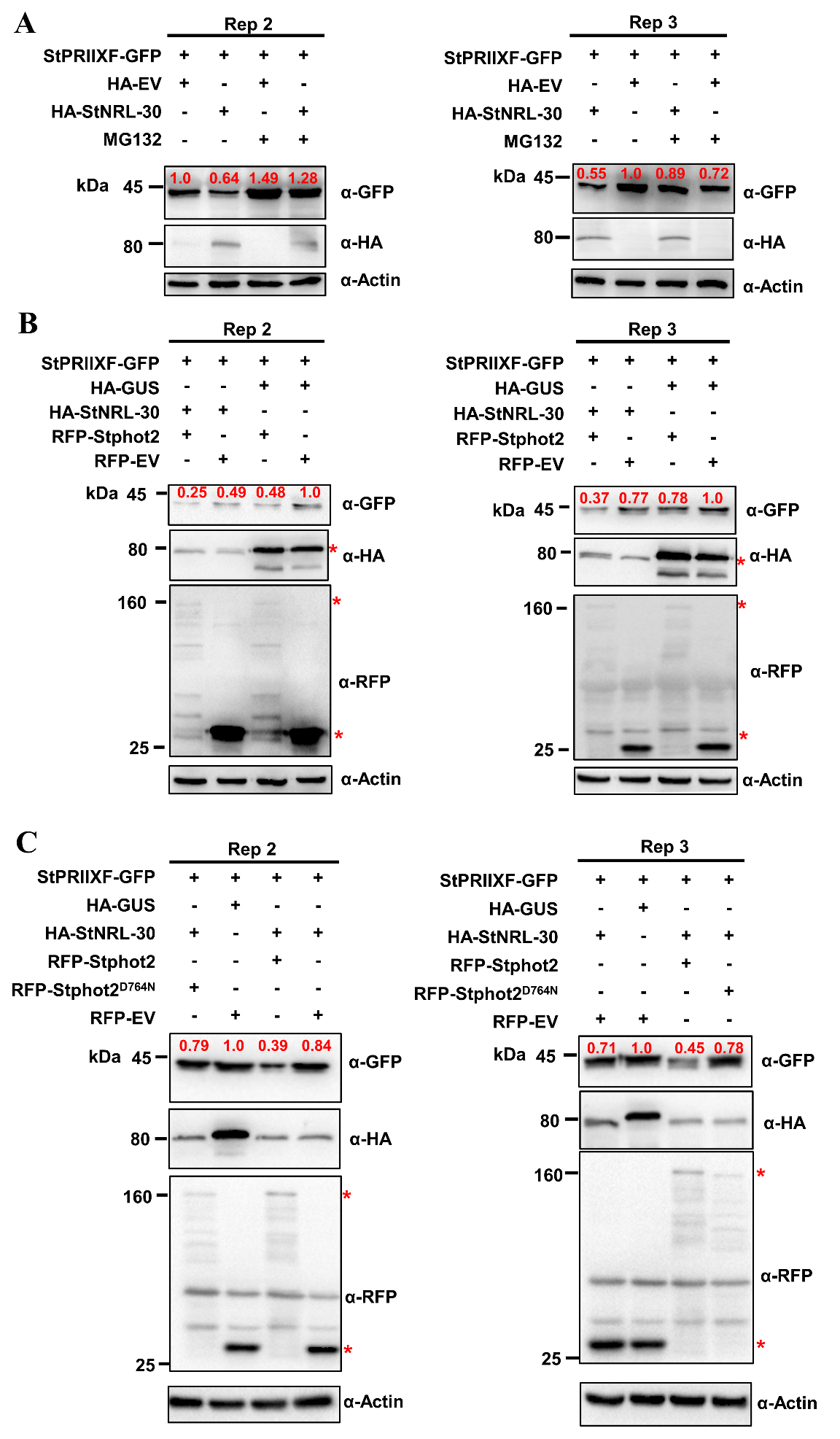


**Fig. S15 Degradation of** **StPRXIIF mediated by StNRL-30 and Stphot2. A** Two additional replicates of immunoblot show that the protein abundance of StPRXIIF-GFP was significantly reduced in the presence of HA-StNRL-30, which was partially recovered upon MG132 treatment. **B** Two additional replicates of immunoblot show that, in the presence of HA-StNRL-30, reduction of StPRXIIF-GFP abundance is accelerated by the co-expression with RFP-Stphot2. **C** Two additional replicates of immunoblot show that the reduction of StPRXIIF-GFP abundance was not accelerated by the co-expression with mutate RFP-Stphot2^D764N^. Construct combinations were expressed in *N. benthamiana* leaves by agroinfiltration. Constructs expression in *N. benthamiana* leaves are indicated by a plus sign (+). Protein sizes are represented in kilodaltons (kDa) and protein loading is shown by Ponceau staining (PS). * indicates target protein bands.


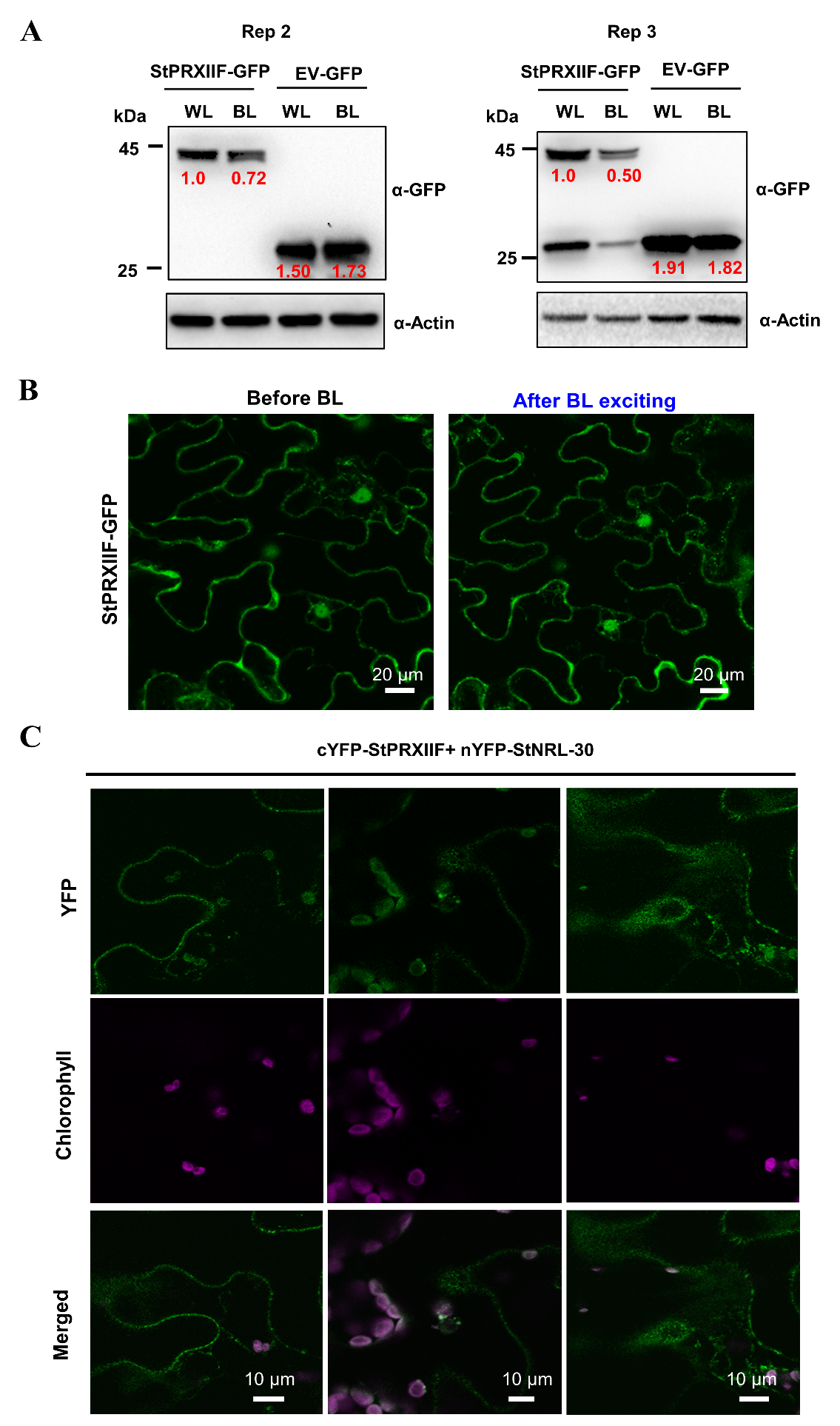


**Fig. S16 Blue light affects stability of StPRXIIF and localization of the StNRL-30**–**StPRXIIF complex. A** Two additional replicates of immunoblot show that the StPRXIIF-GFP abundance was reduced upon blue light (BL) excitation. Protein sizes are represented in kilodaltons (kDa). **B** Confocal images show that the StPRXIIF-GFP localization did not change before or after BL (488 nm laser excitation for about 6 min). **C** Confocal images showing localization of cYFP-StPRXIIF and nYFP-StNRL-30 after BL exciting. Yellow fluorescence was shown in green fluorescence.


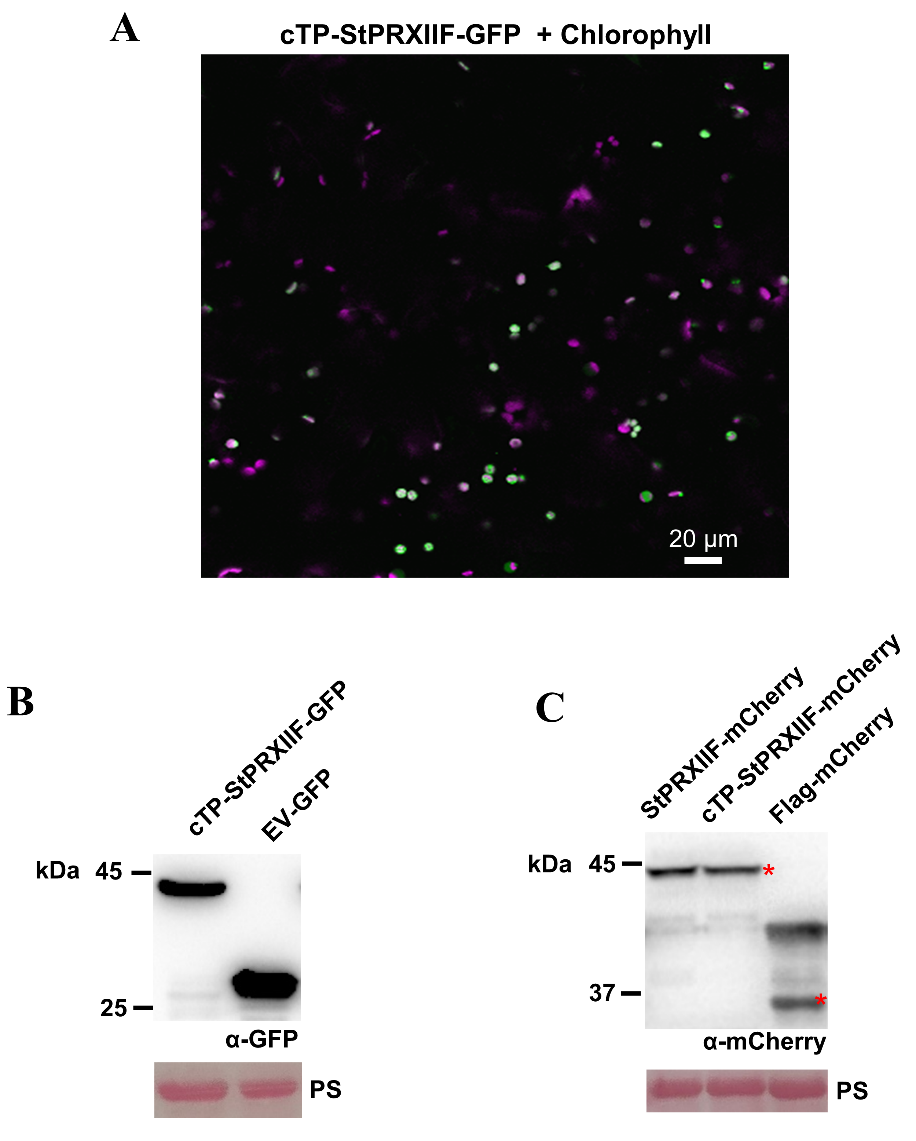


**Fig. S17 cTP-StPRXIIF-GFP is** **stably expressed in *N. benthamiana*.** **A** Merged image showing co-localization of cTP-StPRXIIF-GFP with chlorophyll signal. **B** Immunoblot shows that cTP-StPRXIIF-GFP and EV-GFP were stable when they were transiently expressed in *N. benthamiana* leaves. **C** Immunoblot shows that cTP-StPRXIIF-mCherry, StPRXIIF-mCherry and Flag-mCherry were stable when they were transiently expressed in *N. benthamiana* leaves.
