# Supplementary material for: Blue light receptor phot2 collaborates with NRL-30 to negatively regulate immunity by reducing mitochondrial protein PRXIIF stability in potato
Source: Mol Hortic. 2026 Jun 2;6:44. doi: 10.1186/s43897-025-00224-5 (PMC13227800; doi:10.1186/s43897-025-00224-5)
Supplement: Supplementary file 2 — Supplementary Material 2: Table S1. Putative interaction proteins identified by Y2H library screening. Table S2. Sequence of gene fragments used in silencing constructs. Table S3. Vectors used in this study. Table S4. Primers used in this study. [file 43897_2025_224_MOESM2_ESM.docx]

**Table S1-4**

**Table S1 Putative interaction proteins identified by Y2H library** **screening**

| **Seq No.** | **Gene ID** | **Putative Function** |
| --- | --- | --- |
| 24-4 | Soltu.DM.01G001840.1 | Lignin-forming anionic peroxidase-like |
| **24-1** | **Soltu.DM.01G024400.1** | **Peroxiredoxin IIF** |
| 24-11 | Soltu.DM.02G009450.1 | Protein of unknown function (DUF179) |
| 24-20 | Soltu.DM.03G021990.1 | ARM repeat superfamily protein |
| 24-14 | Soltu.DM.03G031150.1 | Stromal cell-derived factor 2-like protein precursor |
| 24-2 | Soltu.DM.05G020900.1 | Ethylene response factor |
| 24-19 | Soltu.DM.05G025710.1 | DNAJ homologue |
| 24-13 | Soltu.DM.06G002030.1 | Ribosomal protein l14p/L23e family protein |
| 24-22 | Soltu.DM.09G022170.1 | Ribosomal protein l14p/L23e family protein |
| 24-21 | Soltu.DM.09G027290.1 | Ribosomal protein l14p/L23e family protein |
| 24-8 | Soltu.DM.07G022590.2 | Peroxisomal (S)-2-hydroxy-acid oxidase glyoxalase 1 |
| 24-9 | Soltu.DM.08G005300.1 | NAD-dependent malic enzyme |
| 24-7 | Soltu.DM.08G014110.1 | Zinc finger CCCH domain-containing protein 20-like |
| 24-15 | Soltu.DM.11G021230.1 | Histone deacetylase 2C |

**Table S2 Sequence of gene fragments used in silencing constructs.**

| **Gene** | **ID** | **Fragment sequences** |
| --- | --- | --- |
| *Stphot1* | Soltu.DM.11G026060 | GATGAGACTGGGAAATTGCAAGGAGTTAAAGTTAGAACTTCTGGAGATGATGCGAATGGTAAAACAGAAACTTCTCGAAGAGATTCCGGCAACTCTGGTCGGAGTTCCGGCGAATTTTCTGATGATGGAGCTGGTAAAGAGAGGGGAATTCCAAGAGTTTCAGAGGATTTAAGAGATGCCTTATCAACATTTCAACAAACATTTGTGGTATCGGATGCAACCAAACCCGATTACCCGATTCTGTATGCAAGTGCTGGATTTTTCAAGATGACGG |
| *Stphot2* | Soltu.DM.01G037020 | GGATGCCTTAGCGACACTGCAACAGACATTTGTCGTATCTGATGCCACAAAACCAGATTGCCCTATTGTTTATGCTAGTAGTGGCTTCTTTAGTATGACTGGTTATTCTTCAAAAGAAATTGTTGGAAGGAATTGTCGCTTTCTACAGGGGAAAGAAACAGACCAGAAAGAAGTAGCAAAAATCCGAGATGCAGTGAAAACAGGGAAAAGCTACTGTGGGAGGCTCTTAAACTACAAGAAGAATGGCACC |
| *StNRL*-30 | Soltu.DM.09G023040 | TGGCAGAAGTTGCGCTAGATTCGAACCTAACAGCATCGAAGTATATAGCACTTGCAGAGTTACTCCCAGACCACGCACGTCTAGTTTATGATGGATTATATCGAGCTGTAGATATTTTCCTCAAGGTTCATCCAAACATTAAGGACTCTGAACGCTATAGACTCTGTAAATCTATTGACTGTCAGAAACTATCTCAAGAAGCTTGCAGTCACGCAGCTCAAAATGAACGGTTACCTGTGCAAATGGCGGTCCAAGTGCTA |
| *StPRXIIF* | Soltu.DM.01G024400 | GGCTCAAAGCCTGAGATCCTATGCTTCAATTTCAGTAGGAAGTGATGTTACTGTTGCTGCACCAAATGTTTCTCTACAAAAGGCTCGATCTTGGGATGAAGGTGTCTCCTCAAAATTCTCTACTACCCCTGTTAAAGATATCTTCAAGGGTAAAAATGTTGTAATCTTTGGCCTTCCTGGTGCGTACACTGGAGTTTGTTCAATGCAACATGTACCTAGCTACAAGAATAACATTGATAAGTTCAAGGCCAAAGGAATCGACTCTG |
| *Nbphot1* | NbS00003596g0009 | CAATCTCCTCCTCTTATACCACCACTCCCCCGAGATCCAAGAGGCTCACTAGAAGTATTCAACCCATCAACTTACTCTATTTCCCGGCCAAAAAATCCAGTTTTCCGATTATCACAGCCGTCGTGGAACAATAATTGGGCCGAGCCCGAGCTTGAGCCCATTAAAAGAAGCAGCAGCATCCCTGAAACAGAAGAAGAATCAGAGCCTATAGTATCCAATAATAATGATATTAAAGAGGAAACTATTGCCACGTCATGGATGGCAATCAAAGACCCAATTATTATTACTCCAATTTCAACGTCGCAATTGTCTTCTCCGATTACTCAGAC |
| *Nbphot2* | NbS00055894g0005 | AGGTGGAAAGTCGGCTAGTAGTAGAAATGAGGGTATTGATGTGCAAGAAGTTCCAATGAAAGGGGTGGAATCTGGGACTATGACAGGAACAAATAAGCCAATGAACAAGTGGATGGCCTTTGACCCGAATGGGAAAAAAGGAGAAGATAACGGCAATGCAAATAGTCAGATCCCTAGTGAACCCAGCATAGCAGCGAGGGCTGCAGAGTGGGGATTGACAGTGAGGACAGATGTTGGAGAAGGTAGCTTTCACGCAATTAGCAGAAGCGGAGAGAATTCATTTGCAGATGGGGAAAGAGGCAAGAATTCATTGGAG |
| *NbNRL*-30 | Niben101Scf04019g05010 Niben101Scf13748g00017 | TTACGGGTGTTCCTGGCGGATCTGATGCATTTGAACTTGCTGCCAAGTTCTGTTATGGTGTGAACGTTGAGATTACCATATCAAACGTGGCGATGCTAAGATGTGCATCCAAGTTCATGGAAATGAACGAAGACATCTCCGAGAAAAACCTGGAAATTCGTACTGAAATATTCCTTAAAGACACAGTATTCGCAAACATATCCAACTCGATATCTGTTCTTCATCGCTGTGAAACACTACTACCGG |
| *NbPRXIIF* | Niben101Scf01520g08010 | ATGCTTCTGTTTCTGTAGGTACTGATCTTACAGTAGCTGCACCAAATGTTTCACTACAAAAGGCTAGATCTTGGGATGAAGGTGTCTCCTCTAAATTCTCTACTACCCCTCTCAAACATATTTTCAAGGGTAAAAAAGTTATAATCTTTGGCCTTCCTGGTGCATACACCGGAGTTTGTTCAATGCAACATGTACCTAGCTACAAGAATAACATTGATAAGTTCAAGGCCAAAGGAATCG |

**Table S3** **Vectors used in this study.**

| **Vectors** | **Vector type** | **Purpose** |
| --- | --- | --- |
| pH7licNHA | Plant expression vector with N-terminal fusion HA fluorescent protein | Transient expression |
| pH7licNGFP | Plant expression vector with N-terminal fusion GFP fluorescent protein | Transient expression, transgenic |
| pK7WGR2 | Plant expression vector with N-terminal fusion RFP fluorescent protein | Transient expression |
| pH7licCGFP | Plant expression vector with C-terminal fusion GFP fluorescent protein | Transient expression, transgenic |
| pH7licCHA | Plant expression vector with C-terminal fusion HA fluorescent protein | Transient expression |
| pHellsgate8 | RNAi interfering vectors | Transgenic |
| nLUC  (pCAMBIA1300) | C-terminal fusion LUC expression vector | LCA |
| cLUC  (pCAMBIA1300) | N-terminal fusion LUC expression vector | LCA |
| nYFP-gene | N-terminal fusion YFP expression vector | BiFC |
| cYFP-gene | C-terminal fusion YFP expression vector | BiFC |
| PDEST32 | Bait | Y2H |
| pHellsgate8 | RNAi interfering vectors | Transgenic |
| TRV2 | Virus-mediated gene silencing vectors | VIGS |

**Table S4** **Primers used in this study.**

| Prime name | Sequence (5'-3') |
| --- | --- |
| Lic-Stphot2-F | AttacgccgaggTCatggaaaaccaaaggagatcacttg |
| Lic-Stphot2-R | Tagggaagaggctagaataagtccattggatgagcaag |
| Lic-Stphot1-F | AttacgccgaggTCatggaagaagaaaacaagcaatctcc |
| Lic-Stphot1-R | Tagggaagaggttagaaaacatttgtttgtaaatcctccatctc |
| Lic-StNRL-30-F | AttacgccgaggTCATGGGAGTTGTCACTGTTGCTG |
| Lic-StNRL-30-R | TagggaagaggTCAAGACACTGAACGACGCC |
| Lic-CGFP-StPRXIIF-F | tgagCCACCATGGCTGGATCCATGGCCTCAGCAATGTTAAAGAG |
| Lic-CGFP-StPRXIIF-R | cttgctcaccatccgCTCGAGGATCTGTCCTAGGATAACATCTCCG |
| Lic-CHA-StPRXIIF-F | AttacgccgaggTCATGGCCTCAGCAATGTTAAAGAG |
| Lic-CHA-StPRXIIF-R | TagggaagaggGATCTGTCCTAGGATAACATCTCCG |
| pK7WGR2--Stphot2-F | cggcgccgatatcacaagtttgtacatggaaaaccaaaggagatcacttg |
| pK7WGR2--Stphot2-R | gccgcgggatatcaccactttgtacctagaataagtccattggatgagcaag |
| pK7WGR2-StNRL-30-F | cggcgccgatatcacaagtttgtacATGGGAGTTGTCACTGTTGCTG |
| pK7WGR2-StNRL-30-R | gccgcgggatatcaccactttgtacTCAAGACACTGAACGACGCC |
| pGATE8-Xho1-Stphot2-F | TTTGGAGAGGACACGCTCGAGggatgccttagcgacactg |
| pGATE8-Xho1-Stphot2-R | TGGGGTACCGAATTCCTCGAGggtgccattcttcttgtagtttaag |
| pGATE8-Xba1-Stphot2-F | TCATTAAAGCAGGACTCTAGAggtgccattcttcttgtagtttaag |
| pGATE8-Xba1-Stphot2-R | GATAAGCTTGGATCCTCTAGAggatgccttagcgacactg |
| pGATE8-Xho1-StNRL-30-F | TTTGGAGAGGACACGCTCGAGTGGCAGAAGTTGCGCTAGAT |
| pGATE8-Xho1-StNRL-30-R | TGGGGTACCGAATTCCTCGAGTAGCACTTGGACCGCCATTT |
| pGATE8-Xba1-StNRL-30-F | TCATTAAAGCAGGACTCTAGATAGCACTTGGACCGCCATTT |
| pGATE8-Xba1-StNRL-30-R | GATAAGCTTGGATCCTCTAGATGGCAGAAGTTGCGCTAGAT |
| pGATE8-Xho1-Stphot1-F | TTTGGAGAGGACACGCTCGAGgatgagactgggaaattgcaag |
| pGATE8-Xho1-Stphot1-R | TGGGGTACCGAATTCCTCGAGccgtcatcttgaaaaatccagc |
| pGATE8-Xba1-Stphot1-F | TCATTAAAGCAGGACTCTAGAccgtcatcttgaaaaatccagc |
| pGATE8-Xba1-Stphot1-R | GATAAGCTTGGATCCTCTAGAgatgagactgggaaattgcaag |
| pGATE8-Xho1-StPRXIIF-F | TTTGGAGAGGACACGCTCGAGGGCTCAAAGCCTGAGATCCT |
| pGATE8-Xho1-StPRXIIF-R | TGGGGTACCGAATTCCTCGAGCAGAGTCGATTCCTTTGGCCT |
| pGATE8-Xba1-StPRXIIF-F | TCATTAAAGCAGGACTCTAGACAGAGTCGATTCCTTTGGCCT |
| pGATE8-Xba1-StPRXIIF-R | GATAAGCTTGGATCCTCTAGAGGCTCAAAGCCTGAGATCCT |
| Nluc-HA-F | GACGAGCTCGGTACCatggcttcgcgttatcctt |
| Cluc-HA-F | TCCCGGGGCGGTACCatggcttcgcgttatcctt |
| Cluc-GFP-F | TCCCGGGGCGGTACCatggtgagcaagggcgag |
| Nluc-Stphot2-R | CGAGATCTGGTCGACgaataagtccattggatgagcaagtac |
| Nluc-Stphot1-R | CGAGATCTGGTCGACgaaaacatttgtttgtaaatcctccatctc |
| Nluc-StPRXIIF-R | CGAGATCTGGTCGACGATCTGTCCTAGGATAACATCTCCG |
| Cluc-StNRL-4-R | gctctgcaggtcgacCTAAGAAACTGAATGCCTTCCTTTTCTTGAAGG |
| Cluc-StNRL-9-R | GCTCTGCAGGTCGACTTAAGACAGGGAATGCCTCCTACTT |
| Cluc-StNRL-14-R | GCTCTGCAGGTCGACCTAAGAGAGGGAATGCCTCCC |
| Cluc-StNRL-26-R | gctctgcagGTCGACTTATGAAATGGAATTCCTCCTATTTCTTGAAG |
| Cluc-StNRL-28-R | gctCTGCAGGTCGACTTAGGAAACAGAATACCTCCTACTCCTAG |
| Cluc-StNRL-30-R | GCTCTGCAGGTCGACTCAAGACACTGAACGACGCC |
| Cluc-StNRL-31-R | GCTCTGCAGGTCGACTCAGGAAATGGAATTTCTCCATCTTC |
| Cluc-StNRL-32-R | gcctgcagGTCGACTCAAGAAACTGAATATCTTCCTTTTCTTGAAG |
| Cluc-StNRL-15-R | GCTCTGCAGGTCGACTCAGGACAAAGATTTCCTCCACTTG |
| Cluc-StNRL-6-R | GCTCTGCAGGTCGAC |
| Cluc-StNRL1-R | GCTCTGCAGGTCGACTCACGATATGGAATGACGTCTATCT |
| YC-StNRL-30-F | TTCCAGATTACGCTGGATCCATGGGAGTTGTCACTGTTGCTG |
| YC-StNRL-30-R | CCGAATTCACTAGTGTCGACTCAAGACACTGAACGACGCC |
| YN-Stphot2-F | CTGAGGAGGATCTTGGATCCatggaaaaccaaaggagatcacttg |
| YN-Stphot2-R | CCGAATTCACTAGTGTCGACctagaataagtccattggatgagcaag |
| YN-Stphot1-F | CTGAGGAGGATCTTGGATCCatggaagaagaaaacaagcaatctcc |
| YN-Stphot1-R | CCGAATTCACTAGTGTCGACttagaaaacatttgtttgtaaatcctccatctc |
| YN-StPRXIIF-F | AGGAGGATCTTGGATCCATGGCCTCAGCAATGTTAAAGAG |
| YN-StPRXIIF-R | ccgaattcactagtgTCGACCTAGATCTGTCCTAGGATAACATCTCC |
| PDEST32-StNRL-30-F | gctgaaCGAGAAACGGTCGACATGGGAGTTGTCACTGTTGCTG |
| PDEST32-StNRL-30-F | GCCGTTACTTACTTAGAGCTCTCAAGACACTGAACGACGCC |
| TRV-Nbphot1-F | aaggttaccgaattcCAATCTCCTCCTCTTATACCACCA |
| TRV-Nbphot1-R | ctcggtaccggatccGTCTGAGTAATCGGAGAAGACAAT |
| TRV-Nbphot2-F | aaggttaccgaattcAGGTGGAAAGTCGGCTAGTAG |
| TRV-Nbphot2-R | ctcggtaccggatccCTCCAATGAATTCTTGCCTCTTTCC |
| TRV-NbNRL-30-F | aaggttaccgaattcTTACGGGTGTTCCTGGCG |
| TRV-NbNRL-30-R | ctcggtaccggatccCCGGTAGTAGTGTTTCACAGC |
| TRV-NbPRXIIF-F | aaggttaccgaattcATGCTTCTGTTTCTGTAGGTACTG |
| TRV-NbPRXIIF-R | ctcggtaccggatccCGATTCCTTTGGCCTTGAAC |
| qPCR-NbEF1a-F | CCTCAAGAAGGTTGGATACAAC |
| qPCR-NbEF1a-R | TCTTGGGCTCATTAATCTGGTC |
| qPCR-StEF1a-F | ATTGGAAACGGATATGCTCCA |
| qPCR-StEF1a-R | TCCTTACCTGAACGCCTGTCA |
| qPCR-Actin-F | CAGAAAGGACCTCTACGGTAACAT |
| qPCR-Actin-R | TCTGTGGACGATGGACGGAC |
| qPCR-StWRKY7-F | CCAACTGGAAGCAACAACAA |
| qPCR-StWRKY7-R | CCTGATTAGAATGATTAGCCAACA |
| qPCR-StWRKY8-F | CCTACTGTGACATCTCATCAATCC |
| qPCR-StWRKY8-R | GGGTGCTCCCATTTCAGAC |
| qPCR-Stphot1-F | ATGGAAAAATTGGACCGGCG |
| qPCR-Stphot1-R | TGCCGCCTTCGAATTAACCT |
| qPCR-Stphot2-F | TTTCGATGAAAGGGGCGGAA |
| qPCR-Stphot2-R | TGCAGCCCTCTCTGATATGC |
| qPCR-StNRL-30-F | TCGGATCAGCCAGTTTTGCT |
| qPCR-StNRL-30-R | TCCATGAACTTGGCTGCACA |
| qPCR-StPRXIIF-F | TCAAGGCCAAAGGAATCGAC |
| qPCR-StPRXIIF-R | CATACGCCGACCACCTATGA |
| qPCR-Nbphot1-F | AGATGCCTTATCAACATT |
| qPCR-Nbphot1-R | CCAATTCCTGCCTATAAC |
| qPCR-Nbphot2-F | GAAGGCTCTTGAACTACA |
| qPCR-Nbphot2-R | GCATTCCTATAAACTTGATTG |
| qPCR-NbNRL-30-F | GCAGCTAGATCAGGCAATTC |
| qPCR-NbNRL-30-R | TCAAAGTCGTAAACCATTTCGC |
| qPCR-NbPRXIIF-F | ATGAATGGTTGGGCAGAAAAGC |
| qPCR-NbPRXIIF-R | GCCTCTATAACGTCTCCGCC |
